# Therapeutic targeting of endothelial calcium signaling accelerates the resolution of lung injury

**DOI:** 10.1038/s41392-025-02461-y

**Published:** 2025-11-18

**Authors:** Wan Ching Chan, Man Long Kwok, Xinyan Qu, Hazem Abdelkarim, Jonathan Le, Deying Yang, Avik Banerjee, Shuangping Zhao, Jacob Class, Marlen Gonzalez, Harry Hailemeskel, Raman Ghotra Singh, Ricardo Gallardo-Macias, Vadim J. Gurvich, Mark Maienschein-Cline, Matthew Lindeblad, Kasim Kabirov, Alexander V. Lyubimov, Patrick Belvitch, Justin Richner, Vadim Gaponenko, Yulia A. Komarova

**Affiliations:** 1 Department of Pharmacology and Regenerative Medicine, University of Illinois at Chicago, Chicago, IL USA; 2 Department of Biochemistry and Molecular Genetics, University of Illinois at Chicago, Chicago, IL USA; 3 Department of Microbiology and Immunology, University of Illinois at Chicago, Chicago, IL USA; 4 Institute for Therapeutics Discovery and Development, University of Minnesota, Minneapolis, MN USA; 5 Research Informatics Core, University of Illinois at Chicago, Chicago, IL USA; 6 Toxicology Research Laboratory, University of Illinois at Chicago, Chicago, IL USA; 7 Department of Medicine, Division of Pulmonary, Critical Care, Sleep and Allergy, University of Illinois at Chicago, Chicago, IL USA

**Keywords:** Drug discovery, Cell biology

## Abstract

Acute respiratory distress syndrome (ARDS) is a severe pulmonary disease characterized by acute, noncardiogenic pulmonary edema and hypoxemia leading to respiratory failure. It is induced by a diverse array of etiologies, including recent SARS-CoV-2 infection. The current standard of care for ARDS remains predominantly supportive, underscoring the urgent need for targeted pharmacological interventions. To address this critical gap, we developed an inhibitor of the microtubule accessory factor end-binding protein 3 (EB3), a key mediator of pathological calcium signaling in endothelial cells. During injury, EB3 facilitates inositol 1,4,5-trisphosphate receptor 3 (IP_3_R3) clustering on the endoplasmic reticulum membrane, activating widespread calcium release from intracellular stores and leading to endothelial barrier disruption. Using nuclear magnetic resonance (NMR)-guided approaches, we designed and optimized a synthetic EB3 inhibitor, termed vascular therapeutics (VT)-109, with enhanced physicochemical properties. We evaluated the therapeutic potential of VT-109 across a wide range of preclinical models in which pathogenic insults target epithelial or endothelial barriers. Treatment with VT-109 promptly restored the tissue‒fluid balance in the injured lung by inducing the reannealing of VE-cadherin junctions and restoring the endothelial barrier. In addition to vascular protection, VT-109 improved lung architecture and function, normalized immune responses, and significantly reduced both morbidity and mortality in ARDS models. At the molecular level, VT-109 blocks inflammatory NFAT and NF*κ*B signaling while concurrently activating FOXM1-dependent endothelial regeneration. These findings support EB3 inhibition as a promising therapeutic strategy for ARDS and highlight VT-109 as a versatile drug candidate capable of addressing the multifaceted pathophysiology of this disease.

## Introduction

Acute lung injury (ALI) and its more severe clinical manifestation, acute respiratory distress syndrome (ARDS), are among the leading causes of acute respiratory failure and are associated with high morbidity and mortality. These lung disease states, characterized by the rapid onset of noncardiogenic pulmonary edema, arterial hypoxemia, and lung inflammation, can be triggered by various conditions, ranging from sepsis to bacterial or viral pneumonia, including the more recent SARS-CoV-2 infection. Despite recent advances, ARDS mortality remains unacceptably high, with COVID-19-associated ARDS having worse outcomes than ARDS of other etiologies^1^. A crucial component in the pathogenesis of ARDS is the breakdown of the endothelial barrier and the associated interstitial and alveolar edema^2^. Our recent studies highlight the importance of the pulmonary endothelial barrier in both maintaining the lung tissue–fluid balance and supporting the bactericidal activity of recruited immune cells in inflammatory lung conditions^3^. We also showed that pharmacological and genetic strategies targeting vascular leakage can not only promptly restore the tissue‒fluid balance but also induce phenotypic changes in infiltrating neutrophils, enhancing their host defense functions^3^. The pulmonary endothelial barrier is maintained by interendothelial adherens junctions (AJs), which are composed of vascular endothelial (VE)-cadherin and associated catenin protein complexes^4^. These structures limit the passage of plasma proteins and circulating immune cells across the pulmonary endothelial barrier^4^. VE-cadherin junctions are highly dynamic, even at steady state^5^. VE-cadherin undergoes conformational changes and continuous remodeling of adhesion bonds at AJs and is also dynamically recycled between junctional and cytosolic pools^6^. The steady-state balance between VE-cadherin recruitment and internalization determines the density, stability, and restrictiveness of AJs to plasma proteins^7^.

Interestingly, some circulating factors, including proinflammatory cytokines, shift this balance toward VE-cadherin internalization, thereby increasing endothelial barrier permeability^8^. The molecular mechanisms underlying the disassembly of VE-cadherin complexes are multifaceted^9^. Some factors, which act through tyrosine kinase receptors such as VEGFR2, destabilize VE-cadherin interactions with associated p120- and β-catenin proteins in a phosphorylation-dependent manner^8^. Others mechanically disrupt VE-cadherin junctions by increasing actomyosin tension across AJs, pulling them apart^10^. This latter process is driven primarily by G protein-coupled receptor (GPCR) signaling, which activates Gαq/11, leading to the sequential production of inositol 1,4,5-trisphosphate (IP_3_) by phospholipase C and the consequent release of calcium from IP_3_-sensitive stores^11^. In this respect, our work, along with that of others, highlights the key role of the microtubule cytoskeleton in regulating pathological calcium release from endoplasmic reticulum (ER) stores within endothelial cells in response to proinflammatory cues^11–13^. The microtubule-associated factor end-binding protein 3 (EB3) amplifies calcium release from IP_3_-sensitive stores through its specific interaction with inositol 1,4,5-trisphosphate receptor 3 (IP_3_R3) on the ER membrane^11^. Importantly, disrupting this key interaction via a single-point mutation within the binding domain of IP_3_R3^11^ or by targeting the interaction with a cognate peptide^12,13^ prevents pathological calcium release in response to various proinflammatory mediators. This mechanism of vascular leakage appears to be conserved in endothelial cells from diverse tissue types (lung, skin, and eye) and across mammalian species^11–13^. A previous study from our laboratory demonstrated that the interaction between EB3 and IP_3_R3 is further induced in the lung during systemic inflammatory responses to endotoxins^12^. Furthermore, pretreatment of mice with a cognate peptide that disrupts this interaction confers therapeutic benefits by preventing pulmonary microvascular leakage and reducing lung inflammation and injury^11,12^. This intervention also mitigates maladaptive endothelial responses to environmental stresses, such as hypoxia, by normalizing mitochondrial function and enhancing the regenerative capacity of endothelial cells^13^. Thus, targeting pathological calcium signaling by disrupting the EB3-IP_3_R3 interaction has been shown to reduce pulmonary edema and attenuate lung injury^12^.

Previous work has indicated that the resolution of endothelial vascular injury relies on intrinsic mechanisms that allow for the reannealing of VE-cadherin junctions and the restoration of tissue‒fluid balance^14^. One of these mechanisms involves the transcription factor forkhead box protein M1 (FOXM1)^15^. The FOXM1 reparative program promotes both endothelial cell proliferation^16^ and the reannealing of VE-cadherin junctions^17^, facilitating vascular repair^18^. This program, however, is significantly suppressed in elderly patients and COVID-19 ARDS patients^19^, partly because of the failure of transcriptional upregulation of FOXM1 following injury. While delivery of exogenous FOXM1 has demonstrated efficacy in rodent models of ARDS^19^, translating this approach into clinical practice presents significant challenges.

In this study, via the use of a drug-like molecule with improved physicochemical properties, VT-109, a synthetic allosteric inhibitor of EB3, revealed the complex relationship between lung vascular injury and repair. We demonstrated that pathological calcium signaling activates inflammatory pathways while blocking FOXM1-mediated reparative programs in endothelial cells through yet unknown signaling mechanisms. Using multiple preclinical models of ARDS, including polymicrobial sepsis and SARS-CoV-2-induced pneumonia, we showed that blocking pathological calcium release from ER stores with VT-109 promptly restores the tissue‒fluid balance and accelerates endothelial barrier repair by activating the FOXM1 program in endothelial cells. Furthermore, VT-109 was effective regardless of the severity of the injury or the time of treatment, suggesting a promising avenue for future clinical studies.

## Results

### Structure-based design of EB3 inhibitors

Using the parent linear peptide EBIN^13^, we aimed to design drug-like compounds with enhanced physicochemical and biochemical properties, such as solubility, stability, and membrane permeability. The optimizations were made through protection of the C-terminus with an amine^20^; introduction of natural amino acids in D-configurations in either the N- or C-terminal regions; addition of an amide group to the C-terminus; the use of nonproteinogenic (synthetic) amino acids^21^ within the core binding domain; replacement of amino acids with a rigid linker; and rigidification by “stapling” or backbone cyclization. We have also designed 6- and 5-mer truncated linear intermediates for ‘stapling’. In total, we designed and tested thirty-nine linear, twelve cyclic, and ten stapled compounds (Supplementary Table 1).

The solubilities of the synthesized compounds were determined experimentally (Supplementary Table 2). Most compounds were highly soluble at a 1 mM concentration; however, some compounds tended to form aggregates. These compounds were excluded from further studies. In addition, we experimentally determined the lipophilicity of each compound, $\log P({oct}/{wat})=\log (\frac{\left[{solute}\right]{oct}}{\left[{solute}\right]{wat}}$), which is the partition coefficient of a compound between aqueous (water) and lipophilic (octanol) phases (Supplementary Table 2). Our results demonstrated that the lipophilicity values ranged from +0.73 to −1.0, suggesting that none of these compounds were able to penetrate the central nervous system or induce systemic toxicity. Interestingly, some compounds had *logP* values comparable to those of myristoylated (Myr)-EBIN, suggesting that they might have penetrated the plasma membrane without requiring a lipid carrier.

**Fig. 1:**
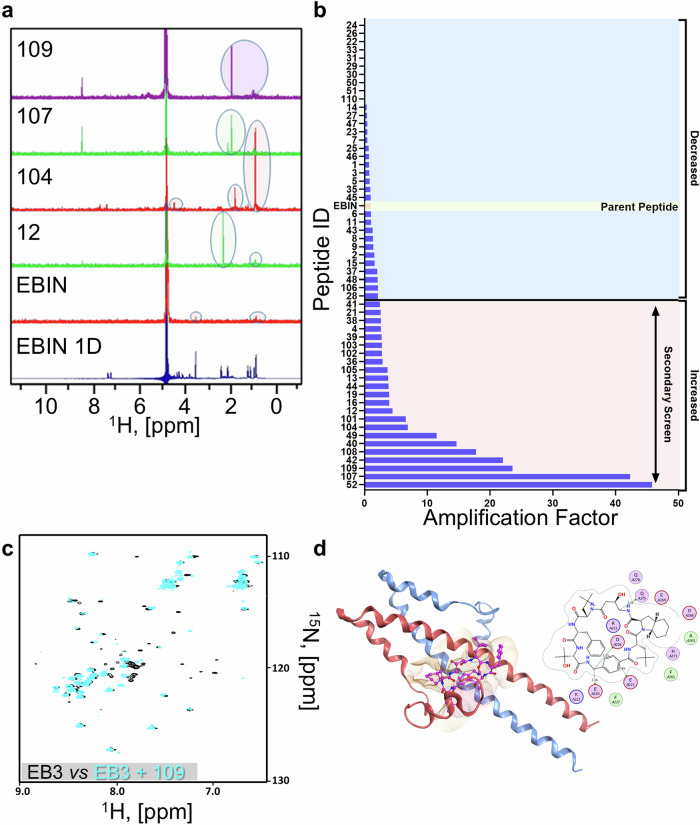
NMR-based characterization of EB3 inhibitors. **a** The saturation transfer difference (STD) NMR approach was used to profile peptide binding to EB3. Examples of STD spectra are shown for peptides 109, 107, 104, 012, and the parental peptide EBIN. The 1D spectrum of EBIN shows the ^1^H signals of the peptide alone. **b** Determination of the top EB3-binding peptides on the basis of the STD amplification ratio plotted *versus* the compound ID. The amplification ratio was calculated as follows: AMP_STD_ = I_STD_/I_NO SAT_ x {L}, where I_STD_ is the highest STD signal intensity measured with saturation; I_NO SAT_ is the intensity measured for the same signal without saturation; and {L} is the peptide concentration. **c** ^1^H-^15^N HSQC spectrum of ^15^N-enriched EB3_200-281_ alone (black) and in the presence of a 1:1 molar ratio of 109 (cyan). Significant spectral changes were observed in the presence of 109. **d** Docking model of EB3 and 109, selected on the basis of alignment between the binding interface and NMR spectral changes. The EB3 dimer is shown as blue and red ribbons representing the two chains of the dimer. Peptide 109 is shown in magenta in the “stick and ball” representation. MOE rendering of the EB3:109 interaction is shown on the right. See also Supplementary Tables 1–5

To screen these drug-like compounds for binding to full-length EB3, we employed the saturation transfer difference (STD)-NMR approach as a cell-free throughput screening platform^22^. We were unable to use other methods, such as isothermal titration calorimetry, because of the nature of the EB3 dimer, which dynamically exchanges chains within the dimer—a process influenced by the binding of the C-terminus to its partners^23^, such as the tested peptides. To analyze the STD-NMR data quantitatively, we established the amplification factor (AMP_STD_), a change in signal intensity upon saturation that is normalized to ligand abundance (Fig. 1a), as a measure of the binding affinity of the compounds to the target protein EB3. Twenty-three out of the sixty-one compounds presented more than 2.5 times greater binding than the parent molecule did (Fig. 1b and Supplementary Table 2). These compounds were subsequently selected for in vitro screening experiments.

Our results suggested that the substitution of the first amino acid in EBIN with D-phenylalanine was well tolerated and even improved binding to EB3 by approximately 2.5-fold. In contrast, either introducing D-isoleucine at the C-terminus or truncating the terminal isoleucine completely abolishes binding (Supplementary Table 2). These findings agree with our structural modeling, which indicates that the C-terminal isoleucine forms critical hydrophobic interactions with EB3^13^. C-terminal amidation of EBIN was also well tolerated, serving to protect the linear parent peptide from enzymatic degradation. Furthermore, simultaneous substitution of two key residues within the EB-binding motif (*TxIP*) with synthetic, protein-like chains (herein, synthetic tool peptides) yields a 4-fold increase in binding affinity to EB3. Notably, replacement of six out of seven amino acids in the linear sequence of EBIN with these protein-like elements still improved binding by 2- to 6-fold. Head-to-tail cyclization of EBIN (compound 012), as well as of linear synthetic tool compounds (e.g., compound 101, the linear precursor to VT-109), improves EB3 binding by approximately 4-fold, highlighting the role of backbone rigidity in increasing binding affinity. We also found that incorporating a short linker to enforce conformational rigidity, referred to here as “stapling”, markedly improved EB3 binding affinity, increasing it by 20- to 40-fold. Importantly, the best-performing compounds consistently adopted more compact and potentially more rigid conformations, which appeared to maximize the binding interface with EB3. Among the various stapling strategies tested, linking the second and last amino acids in the 6-mer synthetic tool compound was the most effective.

We tested the efficacy of twenty-three compounds in inhibiting EB3 interactions with IP_3_R3-GFP via an in vitro approach developed by us^12,13^. Consistent with the STD-NMR findings, all the compounds appeared to show superior efficacy to the parent compound (Supplementary Table 2). These compounds partially inhibited the EB3-IP_3_R3 interaction at a concentration of 250 nM, whereas the parent compound EBIN did not. Furthermore, two-dimensional NMR studies were performed to characterize the binding interface between EB3 and peptide 109 (Fig. 1c). In agreement with the results of the STD studies, peptide 109 caused significant spectral changes (Fig. 1c). Guided by NMR studies, we predicted the binding pose of 109 to EB3 via AutoDock Vina^13,24,25^. NMR-guided docking studies suggested that 109 bound at the protein dimerization interface (Fig. 1d) and displayed a binding pattern similar to that of the parent peptide EBIN^13^.

### Cell-penetrating and endothelial barrier-protective properties of lead and backup inhibitors

**Fig. 2:**
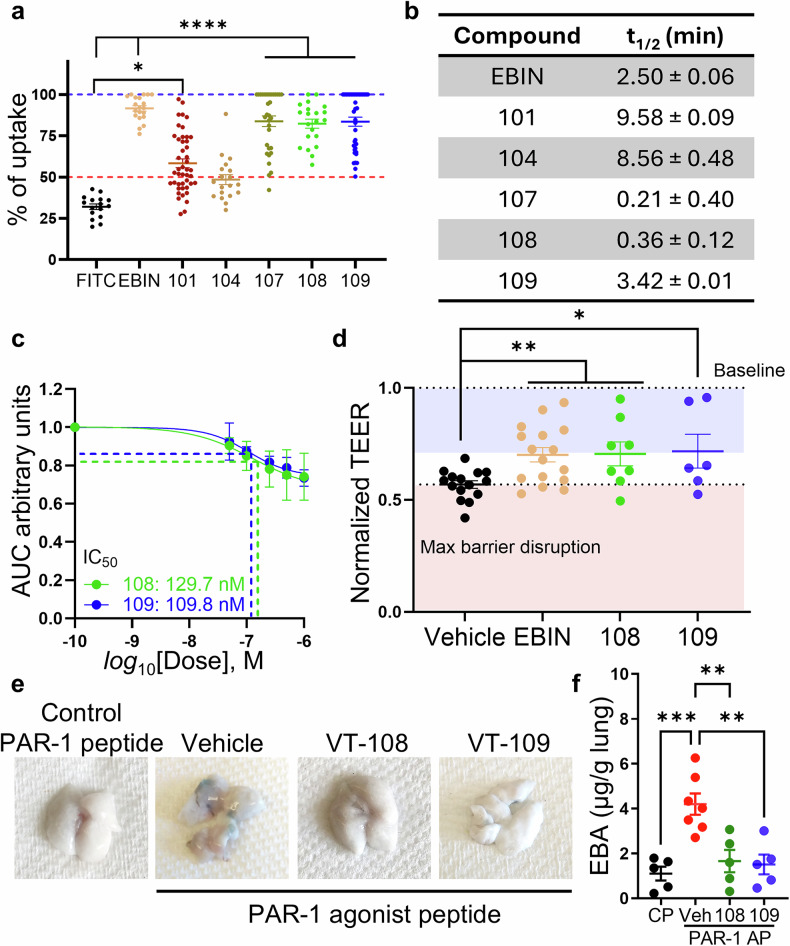
Screening for optimal therapeutic effects via cell culture and murine models. **a** Uptake of selected EB3 inhibitors by pulmonary microvascular endothelial cells, expressed as a percentage of the maximal values obtained following saponin treatment. EBIN, positive control; FITC, negative control. (*n* = 16–45 cells per group). *, *P* < 0.05 and ****, *P* < 0.0001 according to the Kruskal‒Wallis test with Dunn’s multiple comparison test. **b** A table showing the half-time uptake of selected EB3 inhibitors. (*n* = 3 independent experiments). **c** Concentration-dependent curve of intracellular calcium changes induced by α-thrombin *versus* concentrations of the EB3 inhibitors 108 and 109, plotted on a logarithmic scale; 108, green; 109, blue. (*n* = 3 independent experiments). Notably, these experiments were performed in a nominal calcium-free extracellular environment, and the changes in intracellular calcium resulted from ER store deletion, a process that was blocked by EB3 inhibitors in a concentration-dependent manner. **d** Changes in the transendothelial electrical resistance (TEER) of endothelial monolayers pretreated with selected EB3 inhibitors following α-thrombin stimulation. EBIN, positive control; vehicle, negative control. (*n* = 6–16 per group). *, *P* < 0.05 and **, *P* < 0.01 compared with the vehicle control group, as determined via two-tailed t tests. **e**, **f** Representative images (e) and quantification (f) of EBAE in mice challenged with the PAR-1 agonist peptide (AP) following pretreatment with VT-108 or VT-109. Mice treated with the PAR-1 control peptide (FTLLRNPNDK-NH_2_) served as the control group (CP). (*n* = 5–7 mice per group). **, *P* < 0.01 and ***, *P* < 0.001 were determined via one-way ANOVA with Tukey’s post hoc test. Data are presented as the means ± SEMs. See also Supplementary Fig. 1 and Supplementary Tables 1–5

We next analyzed the cell-penetrating properties of the selected compounds, hereafter referred to as vascular therapeutics (VT). The rate of uptake of 5’6-FAM (fluorescein)-labeled compounds by human primary microvascular lung endothelial cells (HLMVECs) was determined via confocal fluorescence live-cell imaging. FAM-labeled Myr-EBIN^13^ and fluorescein alone were used as positive and negative controls, respectively. The t½ uptake and maximal uptake were determined from changes in the integrated fluorescence intensity over a 30 min period (Fig. 2a and Supplementary Fig. 1a). Our results revealed that one linear synthetic compound (VT-101), two stapled synthetic compounds (VT-107 and -108), and one cyclic synthetic compound (VT-109) were able to penetrate the cell membrane without the addition of a cell internalization signal (Fig. 2a). Like Myr-EBIN, compounds VT-107, -108 and -109 presented the highest uptake rates of 92.5 ± 5.0%, 82.5 ± 2.8% and 94.7 ± 4.9%, respectively, whereas the linear compound VT-101 was taken up less efficiently, with an average uptake of 58.4 ± 2.7% (Fig. 2a). Interestingly, these former cell-penetrating compounds had positive *logP* values, indicating a good correlation between cell permeability and lipophilicity (Supplementary Fig. 1b). The t½ uptake, however, varied between the tested compounds (Fig. 2b). The stapled compounds presented the shortest t½ uptake, whereas the linear compounds presented the longest t½ (Fig. 2b). Since the compositions of the linear and cyclic compounds were identical, we concluded that cyclization of the linear compounds significantly improved the t½ uptake. These data demonstrated that rigidification of linear compounds by stapling or cyclization significantly improved the rate of uptake.

We also analyzed the stability of all four compounds, which demonstrated cell-penetrating properties, in human, rat, mouse, and dog blood plasma (Supplementary Tables 3 and 4). Compared with stapled peptides, the linear peptide and its cyclic form, VT-109, were more stable, which was similar to the parent peptide EBIN. Interestingly, the plasma stability data also correlated well with the storage stability, as VT-101, −108, and −109 were stable under various storage and temperature conditions (Supplementary Table 5). Thus, on the basis of their superior cell-penetration properties, blood plasma stability, and storage stability, VT-109 and VT-108 were selected as the lead and backup compounds, respectively, for further analysis in cell culture systems.

To determine the half-maximal inhibitory concentrations (IC_50_) of the two selected compounds, we utilized a Fluo-4 live-cell fluorescence imaging approach with a robotically integrated platform for high-content screening^26^. Fluo-4, a cell-permeant calcium indicator that increases fluorescence upon binding free calcium, was used to measure calcium release from ER stores in primary HLMVECs after treatment with 50 nM human serine protease α-thrombin. These experiments were carried out in a nominal calcium-free environment to prevent store-operated calcium entry following calcium (Ca^2+^) release from the ER stores^27^. VT-108 and −109 demonstrated IC_50_ values of 129.7 ± 0.33 and 109.8 ± 0.26 nM, respectively (Fig. 2c). This effect was specific to endothelial cells, as VT-109 had no effect on the amplitude of ATP-stimulated Ca^2+^ release in mouse alveolar type 2 (AT2) epithelial cells (Supplementary Fig. 1c, d). We also determined the barrier-protective effects of these inhibitors by assessing changes in transendothelial electrical resistance (TEER), a measure of endothelial barrier function^28^, after challenging HLMVECs with the barrier-disruptive proinflammatory mediator α-thrombin. Consistent with our previous findings^11^, both compounds, at a 1 µM concentration, attenuated α-thrombin-induced changes in TEER to the same extent as EBIN did (Fig. 2d).

Furthermore, building on our previous work demonstrating that deletion of EB3 gene (*Mapre3*) in endothelial cells prevents vascular leakage associated with the activation of protease-activated receptor 1 (PAR1)^11^, we measured lung vascular permeability to albumin in mice challenged with a PAR-1 agonist peptide (AP; TFLLRNPNDK-NH_2_), which is known to induce capillary hyperpermeability in the lung (Fig. 2e, f)^11^. Both the lead (VT-109) and the backup (VT-108) compounds, administered intravenously (i.v.) at 1 µmol/kg body weight (bw), a dose selected on the basis of allometric scaling of the in vitro IC_50_, completely blocked PAR-1-mediated capillary leakage in the lung (Fig. 2f). The levels of EBA extravasation in the lungs of mice challenged with PAR-1 AP and treated with these compounds were similar to those observed in control mice treated with a control PAR-1 AP (FTLLRNPNDK-NH_2_), in which the positions of the first two amino acids were reversed.

### VT-109 treats pulmonary edema by inhibiting the inflammatory NFAT and NFκB pathways in endothelial cells

Considering the diverse etiological factors contributing to the pathogenesis of ARDS^29^, we first assessed the therapeutic benefits of VT-109 in treating pulmonary vascular leakage in a murine model of endotoxemia-induced hyperinflammatory ARDS^30^. We induce a systemic inflammatory response by administering lipopolysaccharide (LPS), an endotoxin of the outer membrane of gram-negative bacteria^31^, which elicits strong immune responses in animals^32^, causing endothelial barrier breakdown and associated pulmonary edema, lung injury, morbidity, and mortality^33^. A sublethal dose of LPS, 4 mg/kg body weight (bw), administered intraperitoneally (i.p.) to corn oil-preloaded mice resulted in vascular leakage, peaking on day 3 post-LPS challenge, followed by a resolution phase, with leakage gradually returning to baseline levels by day 15 (Supplementary Fig. 1e, f). Compared with the commonly used model in which the mice were not preloaded with oil, this model exhibited an extended duration of both the injury and resolution phases, enabling the evaluation of a broader therapeutic window.

**Fig. 3:**
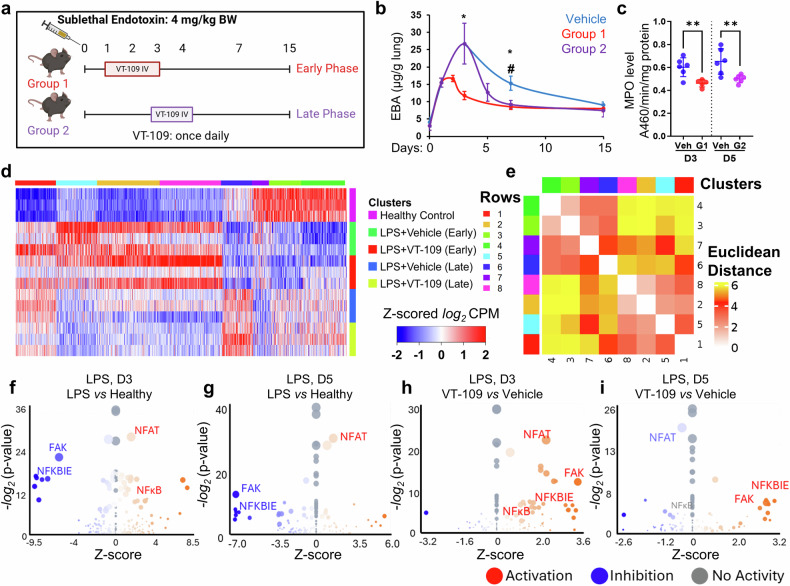
VT-109 reduces vascular leakage by blocking inflammatory pathways in the pulmonary endothelium. **a** Schedule of the treatment of CD-1 mice preloaded with oil and challenged with endotoxin. Treatment was initiated at the onset of lung injury (Group 1) or during the later phase (Group 2). **b** Graph showing EBA extravasation in Group 1 (red) and Group 2 (purple). Vehicle-treated mice served as the control treatment group (blue). (*n* = 5–6 mice per group). * and #, *P* < 0.05 according to two-tailed *t* tests when comparing Groups 1 and 2 against the vehicle, respectively. A smooth line function was used to plot the curve. **c** Myeloperoxidase (MPO) activity in the mouse lung tissue of Group 1 (red) and Group 2 (purple) (*n* = 5–6 mice per group). **, *P* < 0.01 according to two-tailed t tests. **d** Gene expression changes in pulmonary endothelial cells. The experimental groups are shown in Fig. 3a. Each row represents a gene. The color key shows upregulated (red) and downregulated (blue) genes via Z scored log2 counts per million reads mapped (CPM). (*n* = 3 mice per group). **e** Euclidean distance was used to measure the differences in gene expression changes across groups, as shown in (**d**). **f–i** Volcano plots showing the pathways affected by LPS on days 3 (**f**) and 5 (**g**) (LPS *vs* healthy naïve mice) and pathways altered by VT-109 treatment on days 3 (**h**) and 5 (**i**) following LPS challenge (VT-109 *vs* vehicle). Red, activated pathways; blue, inhibited pathways. The bubble size corresponds to the number of genes within each pathway. Statistics were computed with a two-tailed *t* test. (*n* = 3–4 mice per group). Data are presented as the means ± SEMs. See also Supplementary Fig. 1–3

Using this model, we implemented two different i.v. treatment protocols with VT-109 (Fig. 3a, b). In cohort 1, mice received three daily doses of 1 µmol/kg bw VT-109 starting 24 hours after LPS challenge at the onset of lung injury. In Cohort 2, mice received two daily doses, starting 72 hours after LPS challenge, at which time the maximum vascular leakage of plasma proteins was observed in untreated mice challenged with LPS (Fig. 3a, b and Supplementary Fig. 1e, f). Cohort 3 mice received vehicle daily starting at 24 hours of LPS challenge. Our results revealed that treatment of mice with VT-109 promptly blocked the development of pulmonary edema when it was administered at the onset of inflammatory responses on day one but also accelerated the resolution of pulmonary edema when it was administered at the late phase of lung injury (Fig. 3b), indicating that VT-109 has a relatively broad therapeutic window due to its unique ability to accelerate vascular repair. Importantly, regardless of the treatment initiation time, VT-109 consistently lowered the levels of myeloperoxidase (MPO), a marker of neutrophils, in the lung tissue (Fig. 3c). This inhibitory effect of VT-109 on neutrophil recruitment, a hallmark of lung inflammation and poor clinical prognosis^34^, was consistent with our previous work^3^. Overall, our findings indicate that treating pulmonary vascular leakage reduces lung inflammation, in part, by restricting neutrophil transmigration through the endothelial barrier.

To understand how VT-109 restores tissue‒fluid balance at different phases of lung injury, we analyzed transcriptomic changes in freshly isolated pulmonary endothelial cells (CD31^+^/CD45^−^) from mice challenged with a sublethal dose of LPS (as shown in Fig. 3a) via bulk RNA sequencing. Three cohorts of mice were tested. The control group received vehicle treatment, whereas the other two groups received i.v. VT-109 treatment either on day one (the onset of lung injury) or day three (late phase of lung injury) post-LPS challenge (as shown in Fig. 3a). Lung tissues were collected on days three and five post-LPS challenge for Cohorts 1 and 2, respectively. Healthy mice (not challenged with LPS) were used as a baseline control group. The analysis of differentially expressed genes (DEGs) in pulmonary endothelial cells from LPS-challenged mice compared with healthy naïve controls revealed significant (*q* < 0.05) upregulation of 492 and 108 genes and downregulation of 644 and 233 genes on days three and five post-endotoxin challenge, respectively (Fig. 3d, e). Analysis with QIAGEN’s Ingenuity Pathway Analysis (IPA) platform revealed that endotoxin significantly upregulated inflammatory pathways, such as the nuclear factor of activated T cells (NFAT) and nuclear factor kappa B (NFκB) signaling pathways, at both the onset and late phases of lung injury (Fig. 3f, g, and Supplementary Fig. 2a, b). Endotoxin also upregulated the T helper 17 (Th17) activation pathway, which induces endothelial cell senescence (Supplementary Fig. 2a, b)^35^. Furthermore, we observed downregulation of focal adhesion kinase (FAK) signaling, which is responsible for endothelial barrier integrity and cell survival^36^, as well as NFκB inhibitor epsilon (NFKBIE) signaling, an inhibitory pathway of NFκB^37^ (Fig. 3f, g). Overall, these findings suggest that systemic LPS activates a range of converging pathways associated with vascular inflammation and injury.

Compared with vehicle treatment, treatment of LPS-challenged mice with VT-109 led to differential expression changes in 253 and 171 genes (*p* < 0.05) during the onset and late phases of lung injury, respectively (Fig. 3d). VT-109 upregulated both the FAK and NFKBIE signaling pathways regardless of the phase of injury. We also observed that VT-109 inhibited both the NFAT and NFκB inflammatory pathways on day five post-LPS challenge (Fig. 3h, i and Supplementary Fig. 2c, d). These effects were accompanied by downregulation of the farnesoid X receptor/retinoid X receptor (FXR/RXR) activation pathway (Supplementary Fig. 2c, d), a pathway that inhibits angiogenesis, suggesting that VT-109 activated endothelial cell repair^38^. Furthermore, treatment with VT-109 restored the expression levels of 95 genes (*q* < 0.25) to those of the baseline healthy group (Supplementary Fig. 3a). The majority of these genes are associated with the cell cycle and metabolism pathways, indicating that VT-109 elicits its therapeutic effects, in part, through the restoration of cellular functions.

To understand the mechanism by which VT-109 activated reparative and regenerative pathways in endothelial cells during both the onset and late phases of lung injury, we focused on FOXM1 signaling, which was predicted to be highly activated (*p* value = 3.31E−14, activation z score = 5.02) in both the vehicle and VT-109 groups. Consistent with previous work^18^, the activation of the FOXM1 pathway coincided with its transcriptional upregulation in the vehicle-treated groups (Supplementary Fig. 3b). However, the FOXM1 transcript was not significantly upregulated in the endothelial cells of the mice treated with VT-109 compared with those of the healthy control group (Supplementary Fig. 3b), suggesting that VT-109 may activate the FOXM1 transcriptional program independently of its transcriptional regulation. Furthermore, some differences in FOXM1 transcriptional signaling were detected between the vehicle and VT-109 groups (Supplementary Fig. 3c). VT-109 reversed the expression of 32 and 45 FOXM1 target genes during the onset and late phases of lung injury, respectively, restoring them to the levels observed in healthy mice (Supplementary Fig. 3c). These data suggest that pathological calcium signaling is predicted to directly or indirectly suppress FOXM1 transcriptional activity. While the precise relationship between calcium and FOXM1 signaling remains to be fully elucidated, our findings indicate that VT-109 preserves FOXM1 transcriptional activity in endothelial cells during the acute injury phase, thereby counteracting inflammatory signaling and facilitating rapid repair.

### VT-109 resolves pulmonary edema in sepsis by activating the FOXM1 regenerative program

**Fig. 4:**
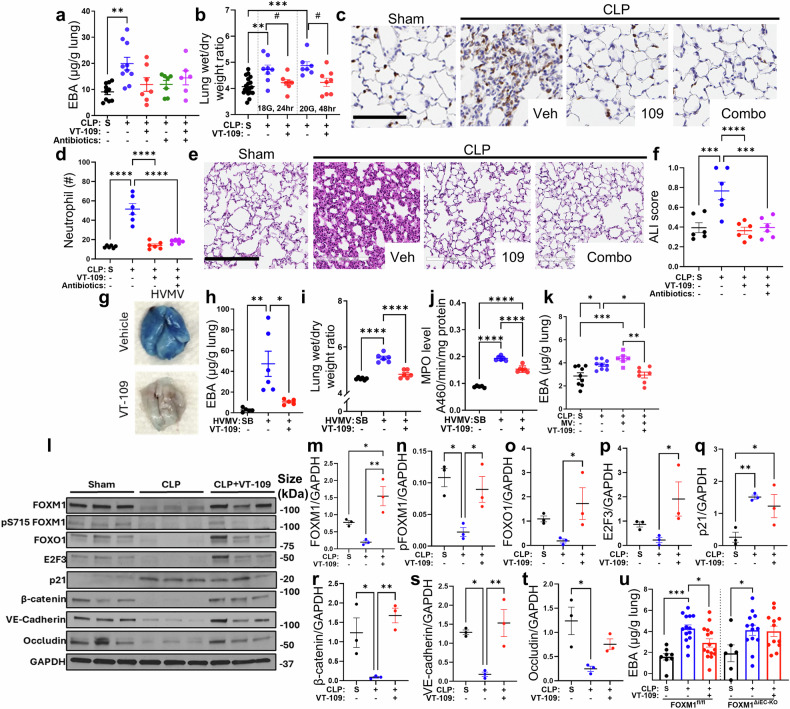
VT-109 restores the lung fluid balance in sepsis by activating the FOXM1 transcriptional program. **a** EBA extravasation in the lung tissue of CD-1 mice 24 hours after CLP surgery. The mice received a single i.v. dose of vehicle (blue), VT-109 monotherapy at 2 µmol/kg bw (red), antibiotic monotherapy (green), or combination therapy with VT-109 and antibiotics (magenta) 5 hours after surgery. The mice that underwent sham surgery (S) served as healthy baseline controls (black). (*n* = 6–10 mice per group). **b** Lung wet‒dry weight ratios of the mice that underwent CLP surgery with either an 18-gauge or a 20-gauge needle were measured at 24 and 48 hours post-surgery, respectively. The mice received treatment as described in (**a**). #, *P* < 0.05, as calculated via two-tailed t tests. (*n* = 7–19 mice per group). **c**, **d** Representative images of mouse lungs stained for lymphocyte antigen 6 family member G (Ly6G) (**c**) and the number of Ly6G-positive cells in the lung tissue (**d**). The mice received treatment as described in (**a**). Scale bar, 200 µm. (*n* = 2 lung lobes per mouse; 3 mice per group). H&E images of lung tissue (**e**) and acute lung injury scores (**f**) from mice subjected to CLP and receiving either VT-109 monotherapy or a combination of VT-109 and antibiotics. Scale bar, 200 µm. (*n* = two lung lobes per mouse; 3 mice per group). Representative images (**g**) and quantification (**h**) of EBA extravasation in the lung tissues of CD-1 mice subjected to mechanical ventilation. The mice received an i.v. injection of either vehicle (blue) or 2 µmol/kg bw VT-109 (red) 30 min prior to ventilation. Spontaneous breathing (SB) mice served as healthy baseline controls (black) (*n* = 5–6 mice per group). **i** Lung wet‒dry weight ratios of CD-1 mice subjected to mechanical ventilation and treated as described in (**g**, **h**) (*n* = 6 mice per group). **j** MPO activity in the lung tissues of CD-1 mice challenged as described in (**g**, **h**) (*n* = 5–6 mice per group). **k** EBA extravasation in the lung tissue of CD-1 mice 25 hours after CLP surgery with a 20-gauge needle. One cohort was placed on normal tidal volume mechanical ventilation for the final 5 hours. The mice received an i.v. injection of either vehicle (blue or magenta) or 2 µmol/kg bw VT-109 (red) at the onset of ventilation. The mice that underwent sham surgery (S) served as healthy baseline controls (black). (*n* = 6–9 mice per group). Western blot analysis of the lung tissues for FOXM1, pFOXM1, FOXO1, E2F3, p21, β-catenin, VE-cadherin, and occludin (**l**) expression 24 hours after CLP with an 18-gauge needle and quantification of protein expression as indicated (**m**–**t**) (*n* = 3 mice per group). *, *P* < 0.05 and **, *P* < 0.01 was calculated via one-way ANOVA with Fisher’s LSD comparison test. **u** EBA extravasation in the lung tissue of FOXM1^fl/fl^ and FOXM1^ΔiEC^ (*Cdh5*-Cre-ERT2) mice 24 hours after CLP surgery via an 18-gauge needle. The treatment groups were as described (*n* = 6–15 mice per group). Data are presented as the means ± SEMs. *P < 0.05, **P < 0.01, ***P < 0.001, and ****P < 0.0001 were calculated via one-way ANOVA with Tukey’s post hoc test unless otherwise indicated. A sham control was used in (**a**, **b**, **h**–**o**). Spontaneous breathing (SB) control was used in (**f**, **g**). A vehicle control was used where VT-109 was administered. See also Supplementary Fig. 4

We next tested the therapeutic benefits of VT-109 in treating vascular leakage and inflammation in polymicrobial sepsis^39^. In these experiments, sepsis was induced via cecal ligation and puncture (CLP) surgery. The CLP model induces polymicrobial sepsis through perforation of the cecum, generating an inoculum derived from native cecal contents, which are produced by the natural microbial flora, including Gram-negative and Gram-positive bacteria, as well as anaerobic, facultative anaerobic, and aerobic organisms^40^. As reported previously^41^, the five most abundant bacterial phyla in the cecal contents and peritoneal cavity following CLP surgery are Firmicutes, Bacteroidetes, Proteobacteria, Tenericutes, and Actinobacteria, enhancing the clinical relevance of this model. In our study, all the animals received two punctures of the cecum via an 18- or 20-gauge needle to induce severe systemic bacteremia. Sham laparotomy mice were used as a baseline control group. Both procedures with either the 18- or 20-gauge needles resulted in significant pulmonary edema at 24 or 48 hours post-surgery, respectively (Fig. 4a, b). In this set of studies, treatment interventions consisted of i.v. VT-109 monotherapy at a dose of 2 µmol/kg bw, antibiotic monotherapy, or a combination of both. Intriguingly, VT-109 monotherapy completely blocked vascular leakage, returning it to the baseline level observed in sham-treated mice, as evidenced by reductions in both Evans blue-labeled albumin extravasation and the wet‒dry lung weight ratio (Fig. 4a, b). The same effects were observed with antibiotic monotreatment or combination therapy (Fig. 4a). We did not, however, observe a synergistic effect of combined therapy on vascular leakage, indicating that both treatment mechanisms were efficient at the dose level used. Furthermore, VT-109 monotherapy markedly suppressed neutrophil recruitment into lung tissue, as evidenced by immunofluorescence staining for the neutrophil-specific marker lymphocyte antigen 6 family member G (Ly6G) (Fig. 4c, d), and prevented the development of acute lung injury (Fig. 4e, f). These effects mirrored those of combination therapy (Fig. 4c–f), suggesting that VT-109 restores both the tissue‒fluid balance and immune homeostasis in the lung. Consistent with these observations, compared with vehicle control treatment, VT-109 monotherapy markedly reduced bacterial colony counts in bronchoalveolar lavage fluid (BALF) (Supplementary Fig. 4a). This may reflect the reduced translocation of bacteria from the systemic circulation into the lung when the endothelial barrier is preserved or potentially enhanced neutrophil-mediated bacterial clearance. However, unlike antibiotics, VT-109 did not affect bacterial counts in the blood or peritoneal fluid (Supplementary Fig. 4b, c), suggesting that its efficacy is not due to direct bactericidal activity but rather to restoring endothelial barrier integrity and improving the immune function of resident and recruited immune cells.

Severely ill ARDS patients undergo intubation and are placed on a mechanical ventilator to maintain adequate oxygen levels in the body^42^. This supportive intervention can have detrimental effects on the lungs, leading to diffuse ventilator-induced lung injury (VILI) in the regions exposed to the highest pressure^43^. Furthermore, VILI primarily targets alveolar epithelial cells^44^, in contrast to indirect models of ARDS, such as endotoxemia and sepsis, where systemic inflammation predominantly injures the pulmonary endothelial barrier^45^. Thus, as a part of a safety study, we investigated the effects of VT-109 on the pathogenesis of VILI. Mice that received vehicle or VT-109 at the most efficacious dose of 2 µmol/kg bw were subjected to high-volume mechanical ventilation (HVMV) at 40 mL/kg bw for 2 hours, and albumin extravasation was measured to determine VILI-induced vascular leakage (Fig. 4g, h). Whereas the vehicle-treated group developed severe vascular albumin leakage, which is consistent with VILI, the mice that received i.v. VT-109 had markedly reduced leakage (Fig. 4g, h). Moreover, VT-109 prevented the development of pulmonary edema and recruitment of neutrophils, as assessed through the wet‒dry lung weight ratio and MPO levels in the lung tissues, respectively (Fig. 4i, j), indicating that VT-109 can also alleviate the hyperinflammatory injuries associated with mechanical ventilation.

Next, to mimic the clinical situation, we induced lung injury by combining CLP surgery with prolonged exposure to a normal tidal volume ventilation of 10 mL/kg bw. In this set of experiments, the mice underwent CLP surgery with a 20-gauge needle, and 20 hours later, they were placed on a ventilator for 5 hours. Whereas CLP alone induced pulmonary vascular leakage, mice subjected to both CLP and ventilation showed exacerbated pulmonary vascular leakage (Fig. 4k). Treatment with i.v. VT-109 at a dose of 1 µmol/kg bw markedly reduced vascular leakage in mice subjected to both exposures, restoring tissue‒fluid homeostasis to baseline levels (Fig. 4k). These findings suggest that VT-109 may be a viable therapeutic option for ARDS patients who have already developed lung injury and are receiving invasive oxygen support. Since some ARDS patients develop acidosis^46^, VT-109 treatment may increase the ventilation volume to improve oxygenation.

We next sought to investigate the effects of VT-109 on activating the FOXM1 regenerative program in endothelial cells. We analyzed the protein expression levels of FOXM1 and its downstream targets, forkhead box protein O1 (FOXO1), cyclin-dependent kinase inhibitor 1 (p21), and E2F transcription factor 3 (E2F3), at 24 hours after CLP with an 18-gauge needle (Fig. 4l–t). We observed that sepsis decreased the levels of FOXM1 and its downstream targets, FOXO1, E2F3, and β-catenin but upregulated p21 in lung tissue (Fig. 4m–r). We also observed downregulation of both VE-cadherin and occludin, which are markers of adherens and tight junctions^47^, respectively (Fig. 4s, t). VT-109 treatment reversed these changes, including those of FOXM1, in addition to p21, suggesting the restoration of FOXM1 transcriptional activity in endothelial cells (Fig. 4m–r). Activation of the FOXM1-driven reparative program was also associated with increased phosphorylation of FOXM1 at Ser715 (Fig. 4n), a posttranslational modification known to increase its transcriptional activity^15^. Furthermore, VT-109 treatment restored both VE-cadherin and occludin expression to the baseline levels observed in sham mice (Fig. 4s, t), supporting the conclusion that VT-109 accelerates vascular repair by promoting the reassembly of adherens and tight junctions. To further validate the effects of VT-109 on vascular repair through a FOXM1-dependent mechanism, we compared the therapeutic benefits of VT-109 in control FOXM1^fl/fl^ and inducible endothelial deletion FOXM1^ΔiEC^ mice. These latter transgenic mice, which carry a *Foxm1* gene with exons 4–7 flanked by loxP sites^48^, were crossed with *Cdh5*-Cre-ERT2 mice. In control mice treated with vehicle, CLP surgery performed with an 18-gauge needle induced a significant increase in vascular permeability to albumin, which was markedly blocked by VT-109 treatment at 2 µmol/kg bw (Fig. 4u). However, VT-109 did not reduce vascular leakage in FOXM1^ΔiEC^ mice. Both vehicle- and VT-109-treated FOXM1^ΔiEC^ mice presented a significant increase in vascular leakage 24 hours after CLP surgery (Fig. 4u). These findings reinforce our conclusion that VT-109 promotes endothelial barrier repair by preserving the FOXM1-driven reparative program during the acute phase of lung injury. Our data indicate that VT-109 reduces vascular leakage, at least in part, by promoting a reparative transcriptional response in endothelial cells to acute stresses. This mechanism appears to be central to the therapeutic effect of VT-109, enabling a shift in cellular signaling from injury-associated pathways toward reparative pathways, thereby mitigating or shortening the duration of the injury state.

### VT-109 markedly reduces morbidity and mortality from septic shock

**Fig. 5:**
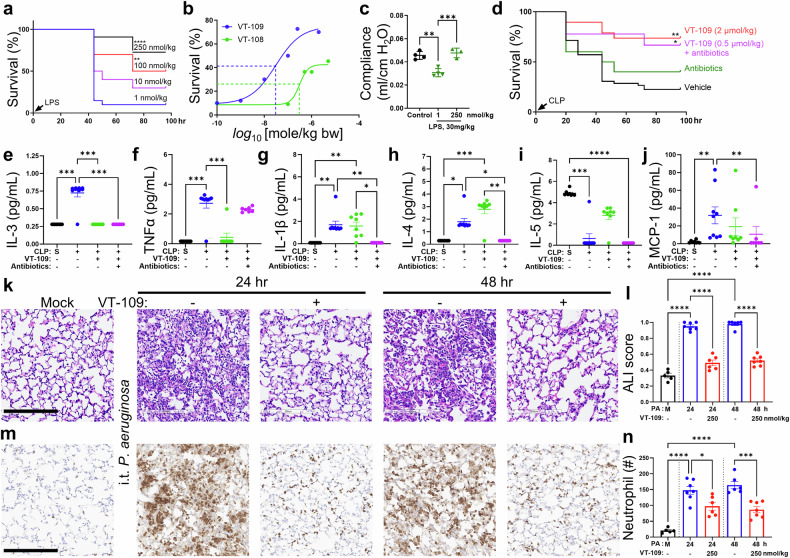
VT-109 improves clinical outcomes in mice with septic shock and bacterial pneumonia. **a** Survival rates of CD-1 mice challenged with a lethal dose 90 (LD_90_) of endotoxin. The mice received the indicated treatments 20 hours post-LPS challenge. (*n* = 10–20 mice per group). **, *P* < 0.01 and ****, *P* < 0.0001 according to the Mantel‒Cox test. **b** Survival curve showing the survival rate as a function of inhibitor concentration; VT-108 (green) and VT-109 (blue) were plotted on a logarithmic scale. (*n* = 10 mice per group). **c** Lung compliance was measured in mice challenged with an LD_90_ dose of endotoxin and treated with either 1 or 250 nmol/kg bw VT-109, as indicated. Control group, healthy mice. (*n* = 3‒4 mice per group) **, *P* < 0.01 and ***, *P* < 0.001 were determined via one-way ANOVA with Tukey’s post hoc test. **d** Survival rate of CD-1 mice that underwent CLP surgery via an 18-gauge needle. The mice received three doses of i.v. vehicle (black) or 2 µmol/kg bw VT-109 (red), 3 doses of the s.c. antibiotic enrofloxacin (5 mg/kg bw; green), or a combination of 0.5 µmol/kg bw VT-109 and enrofloxacin (magenta). (*n* = 10–20 mice per group). *, *P* < 0.05 and **, *P* < 0.01 according to the Mantel‒Cox test. Levels of the inflammatory cytokines IL-3 (**e**), TNFα (**f**), IL-1β (**g**), IL-4 (**h**), IL-5 (**i**), and MCP-1 (**j**) in the BALF of mice 24 hours after sham (black) or CLP surgery. The mice received i.v. vehicle control (blue), 2 µmol/kg bw VT-109 (green), or a combination of antibiotics and VT-109 (magenta) at 5 hours post-surgery. (*n* = 8–10 mice per group). H&E images of lung tissues (**k**) and acute lung injury scores (**l**) of mice infected with intratracheal *Pseudomonas aeruginosa* at 24 or 48 hours as indicated and treated with either vehicle (blue) or VT-109 (red). (*n* = 5–7 mice per group). ****, *P* < 0.0001 was computed via one-way ANOVA with Tukey’s multiple comparisons test. **m**, **n** Representative images of mouse lungs stained for Ly6G (**m**) and the number of Ly6G-positive cells (**n**) found in the lung tissue. Mouse treatment was performed as described in (**k**). Scale bar, 200 µm. (*n* = 5–7 mice per group). *, *P* < 0.05 and ****, *P* < 0.0001 was computed via one-way ANOVA with Tukey’s multiple comparisons test. The data are presented as the means ± SEMs. *, *P* < 0.05; **, *P* < 0.01; ***, *P* < 0.001; and ****, *P* < 0.0001 according to the Kruskal‒Wallis test with Dunn’s multiple comparison test unless otherwise indicated. See also Supplementary Fig. 4 and Supplementary Tables 6 and 7

To establish the effect of VT-109 on clinically translatable outcomes, we investigated its dose-dependent effect on survival rates in a murine model of septic shock (Fig. 5a, d). In experiments involving endotoxin-induced septic shock, mice were injected intraperitoneally with a lethal dose (LD) of LPS at 30 mg/kg bw, which caused 90% mortality. The treatment cohort of mice received i.v. VT-109 at doses ranging from 1 nmol/kg to 2 µmol/kg bw, which were administered once daily for a total of three doses. All the mice also received fluid resuscitation once a day for three days. VT-109 treatment was initiated 20 hours after LPS challenge, at a time when all the mice exhibited comparable levels of illness and clinical signs of systemic inflammation, including diarrhea, labored breathing, and morbidity. While 90% of the mice treated with the lowest dose of VT-109 died within 48 hours post-LPS challenge, the mice treated with efficacious doses of VT-109, starting at 100 nmol/kg bw or higher, presented reduced mortality (Fig. 5a). The half maximal effective concentration (EC_50_) was determined to be 30 nmol/kg bw (equivalent to 2.2 µg/kg bw for humans) on the basis of the therapeutic effects on the survival rate (Fig. 5b). Notably, our backup compound VT-108 also markedly improved survival rates but was less potent and efficacious, with an EC_50_ of 308 nmol/kg bw (Fig. 5b).

We next examined the effect of VT-109 on lung function at 250 nmol/kg bw, the dose that produced the greatest improvement in survival rates (Fig. 5a). Lung function was assessed by measuring lung compliance in mice challenged with a lethal dose of LPS (Fig. 5c). For the control, we used a non-effective dose of VT-109 at 1 nmol/kg bw, as 90% of the mice died before reaching 48 hours post-LPS treatment if left untreated. The 1 nmol/kg bw dose was not efficacious in our mortality studies but did delay death over a 48-hour period. Static lung compliance was measured at 10 cmH_2_O on the expiratory limb of a P‒V loop via the Salazar–Knowles equation^49^. We found that LPS significantly reduced lung compliance to 0.032 ± 0.001 mL/cm H₂O, which is consistent with inflammatory lung injury, whereas treatment with efficacious doses of VT-109 restored lung compliance to 0.043 ± 0.001 ml/cm H₂O (Fig. 5c). These data suggest that VT-109 restores pulmonary function at a therapeutic dose, which is consistent with its ability to reduce pulmonary vascular leakage and promote the resolution of lung injury.

VT-109 also improved the survival rate of mice subjected to CLP surgery when treatment was initiated 5 hours post-surgery (Fig. 5d). In this study, all the animals received two punctures of the cecum via an 18-gauge needle. Notably, a single i.v. dose of VT-109 monotherapy at the most efficacious dose of 2 µmol/kg bw (equivalent to 146 µg/kg bw for humans) or an intermediate dose of 0.5 µmol/kg bw (equivalent to 36.6 µg/kg bw for humans) administered in combination with enrofloxacin, a broad-spectrum veterinary fluoroquinolone commonly used in mice^50^, significantly improved the survival rate compared with that of the controls (Fig. 5d). While neither antibiotics nor VT-109 monotherapy (0.5 µmol/kg bw) significantly improved survival in mice subjected to CLP surgery, combined treatment resulted in a marked increase in survival, suggesting additive or synergistic effects of the dual intervention. Although we recognize that enrofloxacin is approved only for veterinary use and that the effects of VT-109 in combination with clinically used antibiotics remain unknown, we believe VT-109 is likely compatible with other classes of broad-spectrum antibiotics. These findings indicate that lower doses of VT-109 may be effectively used in combination with antibiotics to treat sepsis, whereas higher doses may be necessary in cases involving antibiotic-resistant infections.

Consistent with the therapeutic effects of VT-109 monotherapy on survival, we observed reduced levels of alanine aminotransferase (ALT) and blood urea nitrogen (BUN) (Supplementary Fig. 4d, e), which are markers of liver^51^ and kidney^52^ damage, respectively. In addition, VT-109 monotherapy significantly reduced interleukin (IL)-3 and tumor necrosis factor alpha (TNFα) levels in BALF (Fig. 5e, f), with no significant changes in the other cytokines or chemokines included in this analysis (Supplementary Table 6). Importantly, VT-109 treatment did not affect any of the tested cytokines in the peritoneal fluid, the site of primary infection (Supplementary Table 7), nor did it directly alter cytokine production by immune cells in vitro (Supplementary Fig. 4f–i).

The therapeutic effects of VT-109 were further enhanced when it was combined with antibiotics. In this setting, we observed a significant reduction in the levels of multiple proinflammatory cytokines, including IL-1β, IL-3, IL-4, and IL-5, as well as monocyte chemoattractant protein-1 (MCP-1) (Fig. 5e–j, Supplementary Table 6). These data, along with the improved survival of the mice (Fig. 5d), consistently demonstrated that combination therapy provided additional benefits in mitigating the cytokine storm and reducing mortality. We posit that VT-109 mitigated systemic inflammatory responses indirectly by dampening the activation of endothelial cells in response to endotoxins and cytokines, thereby restoring barrier integrity. Our results indicated that VT-109 effectively treated pulmonary edema, reduced both lung and systemic inflammation and ultimately prevented multiorgan failure and mortality in mice with septic shock.

### VT-109 effectively treats diffuse alveolar damage in both bacterial and viral pneumonia

Most ARDS cases arise secondary to pneumonia, in which infectious agents directly target the alveolar epithelium^53^. Damage to these epithelial cells disrupts the integrity of the alveolar–capillary barrier, leading to protein-rich pulmonary edema and widespread inflammation, two hallmark features of diffuse alveolar damage^53^. Because endothelial barrier disruption is a downstream consequence in this context, we also evaluated the benefits of VT-109 treatment in a model of *Pseudomonas aeruginosa*–induced lung injury^54^, the most commonly acquired hospital-associated pneumonia^55^. Mice were infected with a sublethal intratracheal dose of 5 × 10^5^ CFU *Pseudomonas aeruginosa* and treated with a moderately efficacious dose of VT-109 (250 nmol/kg bw) at 5 hours post-challenge. The lung tissues were collected at 24 and 48 hours for histopathological analysis of acute lung injury, neutrophil influx, and bacterial counts in BALF (Fig. 5k–n and Supplementary Fig. 4j). In this study, vehicle-treated control mice challenged with the pathogen exhibited massive neutrophil recruitment into the alveolar space by 24 hours (Fig. 5k–n), which progressed to diffuse alveolar damage, interstitial pneumonia, and tissue consolidation by 48 hours (Fig. 5k–n). In contrast, mice treated with VT-109 presented far fewer neutrophils in the lung tissues that localized primarily to the perivascular space and interstitium, and the lungs appeared uninjured (Fig. 5k–n). Lung pathology did not worsen between 24 and 48 hours in VT-109–treated animals (Fig. 5k–n), suggesting that VT-109 mitigated alveolar damage by blocking massive neutrophil infiltration. We also observed a modest reduction in bacterial counts in VT-109-treated mice at both time points, although this difference did not reach statistical significance (Supplementary Fig. 4j). Given that the number of neutrophils was significantly lower in the lungs of mice treated with VT-109, these data suggest that reannealing VE-cadherin junctions in the acute setting of infection may enhance the antimicrobial function of immune cells, as we previously reported^3^. Encouraged by these results, we further assessed the therapeutic benefits of VT-109 in diffuse alveolar damage caused by viral infection.

**Fig. 6:**
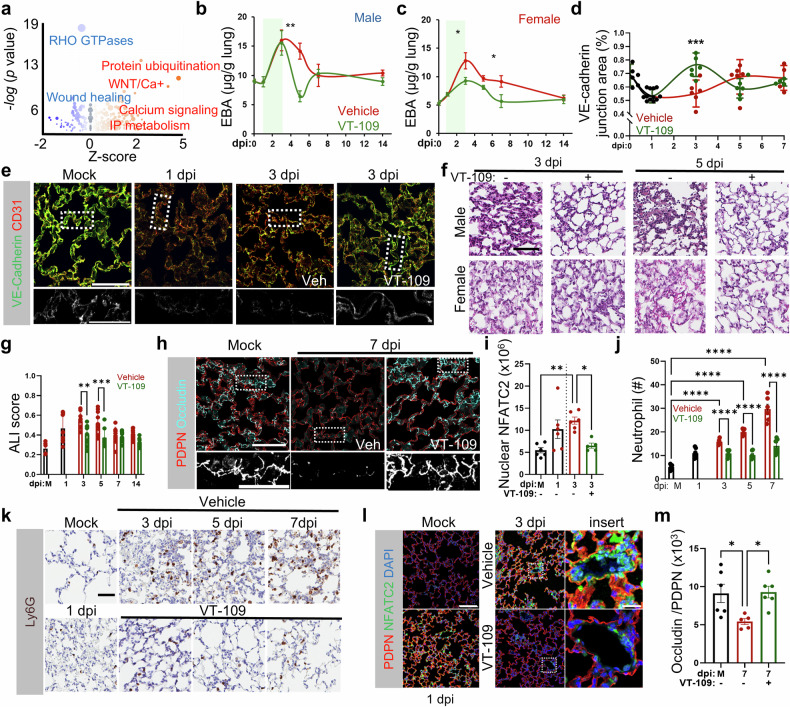
VT-109 promotes the resolution of lung injury following SARS-CoV-2 MA10-induced pneumonia. **a** Analysis using QIAGEN’s IPA of pulmonary endothelial cells from COVID-19 patients compared with those from healthy controls, based on data from Melms et al. (see Supplementary Materials for Methods). Red, activated pathways; blue, inhibited pathways. The bubble size corresponds to the number of genes within each pathway. Statistics were computed with a two-tailed *t* test. EBA extravasation in the lung tissue of male (**b**) and female (**c**) BALB/c mice infected via intranasal (i.n.) inoculation with 1 × 10^4^ PFU of the MA10 virus and treated with either vehicle (red) or VT-109 (green). The mice received 3 daily doses of treatment starting on day 1 post infection. (*n* = 5–6 mice per group). The treatment duration is indicated by green shading. A smooth line function was used to plot the curve. *, *P* < 0.05 and **, *P* < 0.01 were computed via two-tailed *t* test between VT-109 and vehicle groups on their respective days. Quantification of the VE-cadherin-PECAM1 overlapping junction area (**d**) and associated representative images of lung tissues stained for VE-cadherin (green) and CD31 (red) (**e**). The insert shows VE-cadherin in grayscale. BALB/c mice were infected and treated as described in (**b**, **c**). A smooth line function was used to plot the curve. Scale bar, 50 µm. Insert, 20 µm (*n* = 6–10 mice per group). ***, *P* < 0.001 was computed via two-tailed *t* test between VT-109 and vehicle groups on their respective days. H&E images of the lung tissues (**f**) and acute lung injury scores (**g**) of BALB/c mice infected and treated as in (**b**, **c**) (*n* = 15–20 fields per mouse, 3–5 mice per sex per group). Scale bar, 60 µm. **, *P* < 0.01 and ***, *P* < 0.001 were determined via two-way ANOVA with Sidak’s multiple comparison test. Images of lung tissues stained for PDPN (red) and occludin (cyan) (**h**) and quantification of the occludin-positive signal within epithelial cells (**i**). The insert shows occludin in grayscale. Scale bar, 50 µm. Insert, 20 µm (*n* = 6 mice per group). Quantification of Ly6G-positive cells at the indicated times following infection (**j**) and the associated representative images of lungs from BALB/c mice stained for Ly6G (**k**). Infection and treatment conditions are described in (**b**, **c**). The vehicle group, red; the VT-109 group, green. Scale bar, 50 µm. (*n* = 15 fields per mouse, 6 mice per group) ****, *P* < 0.0001 was computed via two-way ANOVA with Tukey’s multiple comparison test. **l**, **m** Representative images of mouse lungs stained for PDPN (red), NFATC2 (green) and nuclei (DAPI, blue) (**l**) and quantification of NFATC2 nuclear accumulation at the indicated days post-infection (**m**). Scale bar, 100 µm. Insert, 20 µm. (*n* = 5 fields per mouse, 5–6 mice per group). Data are presented as the means ± SEMs. *, *P* < 0.05 and **, *P* < 0.01 were determined via one-way ANOVA with Tukey’s post hoc test unless otherwise indicated. See also Supplementary Figs. 5, 6

In SARS-CoV-2-induced ARDS, the initial injury also primarily affects alveolar epithelial cells, particularly type II pneumocytes expressing ACE2^56^, with subsequent inflammation and barrier dysfunction contributing to secondary endothelial injury^57^. Analysis of a public scRNA-seq dataset^58^ via QIAGEN’s IPA platform revealed upregulation of calcium signaling and ubiquitination pathways in lung endothelial cells from COVID-19 patients compared with those from healthy individuals (Fig. 6a). Given the critical role of calcium signaling in COVID-related lung pathology, we next evaluated the therapeutic potential of VT-109 in BALB/c mice infected with the mouse-adapted SARS-CoV-2 MA10 virus^59^. This model was characterized by a significant increase in viral genome levels within lung tissues starting on day 1 post-infection, followed by a gradual decline by day 14 (Supplementary Fig. 5a**)**^59^. However, very low levels of virus were detected in the brain, heart, and kidney (Supplementary Fig. 5b–d), suggesting that the virus was contained in the lung. The treatment of infected mice starting on day 1 post-infection with three daily i.v. doses of VT-109 (250 nmol/kg bw), identified as the optimal dose for improving survival in LPS-challenged mice (Fig. 5a), did not alter SARS-CoV-2-MA10 genomic RNA levels (Supplementary Fig. 5a–d) or the number of infectious viral particles (Supplementary Fig. 5e, f). Consistent with these results, VT-109 treatment did not change the ACE2 expression levels in the lungs (Supplementary Fig. 5g, h). These findings suggested that VT-109 did not alter viral replication or cellular invasion.

Infection with the MA10 virus caused significant pulmonary vascular leakage in both sexes, peaking at 3 days post-infection (dpi) and returning to baseline levels between 7 and 14 dpi (Fig. 6b, c). The mice that received i.v. treatment with VT-109 (as above) presented a reduction in vascular leakage on day 5 for males (Fig. 6b) and days 3 and 7 for females (Fig. 6c). The resolution of vascular leakage coincided with the reannealing of VE-cadherin junctions at 3 dpi in VT-109-treated mice, as evidenced by increased VE-cadherin and PECAM1 double-positive areas marking interendothelial adherens junctions (Fig. 6d, e**)**.

Histopathological analysis of the lung tissues revealed interstitial edema and hemorrhage at 1 dpi, which progressed to lung consolidation, alveolar wall thickening, hyaline membrane formation, and infiltration of neutrophils and other immune cells into the interstitial and alveolar spaces by days 3 and 5 (Supplementary Fig. 5i). Interestingly, VT-109-treated mice rapidly resolved interstitial edema, reduced alveolar wall thickening, diminished lung consolidation by day 3, and restored normal lung architecture by day 5 (Fig. 6f, g), characteristics of lung repair. Consistent with enhanced tissue regeneration, VT-109 treatment promptly suppressed endothelial cell death (Supplementary Fig. 6a, b) and increased the number of Ki-67-positive endothelial and surfactant protein C (SPC)-positive epithelial cells, indicating active proliferation of both alveolar endothelial and epithelial cells during the recovery phase (Supplementary Fig. 6c–f**)**. These effects were more pronounced at 3 dpi than in the vehicle-treated controls, demonstrating that VT-109 not only mitigated injury but also accelerated repair.

Further analysis revealed that VT-109 accelerated the ‘productive’ transdifferentiation of alveolar type 2 (AT2) to type 1 (AT1) cells. Keratin-8 (KRT8) staining, a marker of AT2 to AT1 transition, revealed a transient surge in transitioning cells at 3 dpi, with the completion of transdifferentiation by day 5 in the VT-109 group but not in the vehicle group (Supplementary Fig. 6g, h). As a result, occludin tight junction reannealing in AT1 cells was observed at 7 dpi in treated lungs (Fig. 6h, i), indicating successful restoration of the alveolar-capillary barrier. We conclude that VT-109 not only restores tissue-fluid homeostasis but also enhances AT2 → AT1 transdifferentiation, an often-impaired process in COVID-19 patients, where cells remain trapped in a transitional state^60^. Overall, these data underscore the potency of VT-109 against SARS-CoV-2–associated ARDS.

We next explored how VT-109 mitigates lung inflammation and restores immune homeostasis. In vehicle-treated mice, Ly6G staining revealed a progressive increase in neutrophil influx into the interstitial and alveolar spaces over 7 days post-infection (Fig. 6j, k). In contrast, in VT-109–treated mice, neutrophil numbers recruited by day 1 did not further increase after treatment initiation. (Fig. 6j, k). Given that VT-109 mitigates NFAT signaling in endothelial cells in mice challenged with LPS (Fig. 3d–i) and that SARS-CoV-2 nonstructural protein 1 (Nsp1) induces a proinflammatory milieu by activating the calcium-calcineurin-NFAT axis^56^, we assessed NFATC2 nuclear localization, a prerequisite for pathway activation^61^. SARS-CoV-2 infection triggered NFATC2 translocation into the nuclei of endothelial and other cells by day 1, persisting through day 3 post-infection (Fig. 6l, m). VT-109 treatment, however, reduced the level of nuclear NFATC2 to mock-infected levels (Fig. 6l, m), indicating that VT-109 suppresses endothelial NFAT signaling. Together, these findings suggest that VT-109 restores both tissue-fluid and immune balance by strengthening endothelial junctions and blocking NFAT-driven endothelial activation to limit neutrophil extravasation.

Furthermore, we sought to test the effects of VT-109 in treating more severe forms of pulmonary injury induced by the Washington strain of SARS-CoV-2 (WA1/2020). In this model, transgenic mice expressing human ACE2 in AT2 cells driven by the K18 promoter (K18-hACE2)^62^ were inoculated intranasally with 1 × 10^4^ PFU of WA1/2020 SARS-CoV-2. In contrast to the MA10 model, these K18-hACE2 mice presented a sustained increase in SARS-CoV-2 genome levels until they reached the humane end point between 6 and 7 dpi (Supplementary Fig. 7a). Consistent with previous reports^62^, these mice infected with WA1/2020 SARS-CoV-2 developed severe neurological pathology and brain damage associated with the neurodissemination of the virus^63^. We confirmed the presence of the virus in the brain tissue of these mice, while tests on the heart and kidneys revealed few to no detectable virus (Supplementary Fig. 7b–d).

**Fig. 7:**
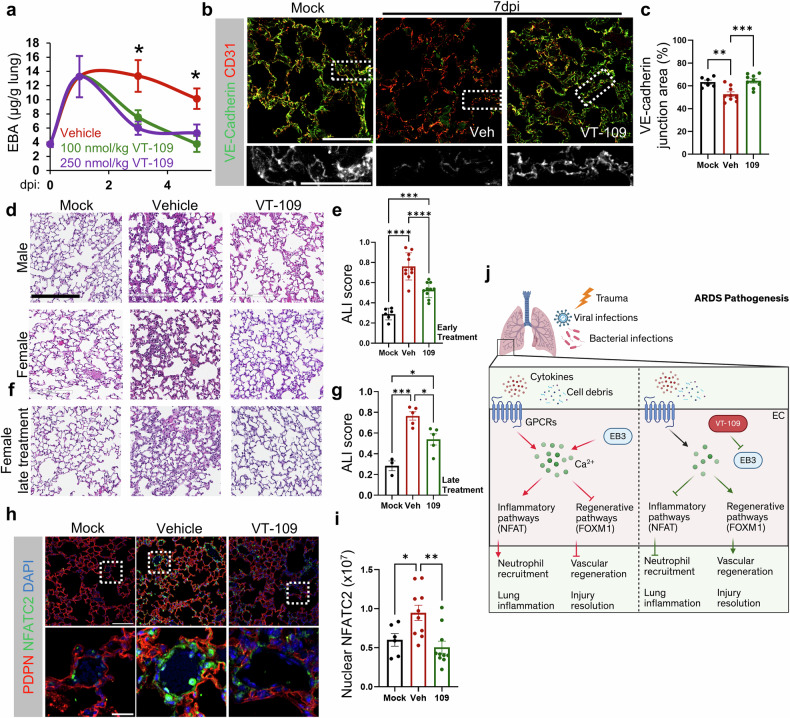
VT-109 treats severe form of lung injury in hACE2 transgenic mice infected with the Washington strain of SARS-CoV-2. **a** EBA extravasation in male K18-hACE2 mice infected via i.n. inoculation with saline (mock) or 1 × 10^4^ PFU of the Washington strain of SARS-CoV-2, as assessed at the indicated days post-infection (dpi). Treatment groups: i.v. vehicle control (red), daily dose of 100 nmol/kg bw (green) or 250 nmol/kg bw (magenta) VT-109 was administered starting at 1 dpi for 3 consecutive days. (*n* = 5–12 mice per group) *, *P* < 0.05 according to two-tailed t tests comparing the VT-109 and vehicle groups on their respective days. A smooth line function was used to plot the curve. **b**, **c** Representative images of mouse lungs at 7 dpi stained for VE-cadherin (green) and CD31 (red) (**b**) and quantification of the VE-cadherin-PECAM1 overlapping junction area (**c**). The insert shows VE-cadherin in grayscale. The treatment groups, vehicle control (red) and 250 nmol/kg bw VT-109 (green), are shown. Scale bar, 50 µm. Insert, 20 µm (*n* = 5 fields per mouse, 6–10 mice per group). Representative H&E-stained lung images (**d**) and quantification of the ALI score (**e**) in K18-hACE2 mice at 7 dpi. The treatment groups are the same as those in (**b**). Scale bar, 200 µm. (*n* = 15–20 fields per mouse, 6–10 mice per sex per group). Representative H&E-stained lung images (**f**) and quantification of the ALI score (**g**) at 7 dpi in female mice infected with SARS-CoV-2 and treated with either vehicle (red) or 250 nmol/kg bw VT-109 (green) starting at 3 dpi. (*n* = 15–20 fields per mouse, 3–5 mice per group). Representative images of lungs stained for PDPN (red), NFATC2 (green) and nuclei (DAPI, blue) (**h**) and quantification of the nuclear accumulation of NFATC2 (**i**) in the lungs of infected mice treated with vehicle (red) or 250 nmol/kg bw VT-109 (green) starting at 1 dpi. Scale bar, 100 µm. Insert, 20 µm. (*n* = 5 fields per mouse, 6–10 mice per group). **j** Model describing the therapeutic effects of VT-109 in treating common clinical manifestations of ARDS. This figure panel was created via BioRender.com. Data are presented as the means ± SEMs. *P < 0.05, **P < 0.01, ***P < 0.001, and ****P < 0.0001 were computed via one-way ANOVA with Tukey’s post hoc test unless otherwise indicated. See also Supplementary Fig. 7

SARS-CoV-2 infection induced a continuous increase in microvascular permeability throughout the study, beginning on day 1 in vehicle-treated K18-hACE2 mice (Fig. 7a). However, the pulmonary vascular permeability of the mice that received three daily i.v. injections of VT-109 starting at 1 dpi at efficacious doses of 100 or 250 nmol/kg bw was similar to that before infection at 5 and 7 dpi (Fig. 7a), indicating the resolution of pulmonary edema. Like in the MA10 model, pulmonary vascular leakage in the vehicle-treated group was associated with the loss of VE-cadherin junctions, whereas VT-109-treated mice had reannealed junctions at 7 dpi (Fig. 7b, c).

Furthermore, SARS-CoV-2 caused sustained lung injury (Supplementary Fig. 7e). However, VT-109 treatment promoted the resolution of lung injury by day 7 (Fig. 7d, e), highlighting its efficacy in treating lung injuries caused by WA/2020 SAS-CoV-2. Interestingly, initiating VT-109 treatment at 3 dpi produced similar beneficial effects on lung repair (Fig. 7f, g), indicating a broad therapeutic window for VT-109. Compared with vehicle, VT-109 also reduced NFATC2 nuclear accumulation in endothelial and immune cells at 7 dpi (Fig. 7h, i), suggesting that VT-109 has the potential to alleviate inflammation in COVID-19-induced pneumonia. Furthermore, analysis of immune cells in the BALF of SARS-CoV-2-infected mice revealed that treatment with the high efficacious dose of VT-109 (2 µmol/kg bw), which was selected to assess the maximal therapeutic effect, significantly reduced the number of infiltrating monocytes and neutrophils while preserving alveolar macrophages (Supplementary Fig. 7f–h). These findings are consistent with the proposed role of VT-109 in maintaining immune cell homeostasis within the injured lung (Figs. 3c, 4c, d, 5m, n, and 6j, k).

We also evaluated the effects of VT-109 on vascular leakage in the cerebral cortex of SARS-CoV-2-infected K18-hACE2 mice, a model known to exhibit blood-brain barrier (BBB) disruption^63^. Our data indicate that i.v. treatment with 2 µmol/kg bw VT-109 restored BBB integrity, as evidenced by reduced extravasation of fibronectin within the vascular wall (Supplementary Fig. 7i, j). Thus, VT-109 is anticipated to extend its benefits beyond the lung, offering multi-organ protection against SARS-CoV-2–induced injury.

### VT-109 has a broad safety margin, favorable pharmacokinetics, and efficient lung deposition

A non-GLP dose-escalation study was conducted to determine the maximum tolerated dose (MTD) of VT-109 following a single intravenous (IV) bolus administration in Crl:CD (SD) rats. Male rats (*n* = 3 per group) received escalating doses, with at least 48 hours between cohorts. All the animals were euthanized on day 3 post dose. At doses of 10 and 15 mg/kg, the rats presented transient clinical signs, including labored breathing, decreased activity, and piloerection; one animal in the 15 mg/kg group also exhibited a hunched posture (Supplementary Table 8). These symptoms were resolved by day 2. Lung gross histopathology at 15 mg/kg revealed intra-alveolar hemorrhage and pigment deposition, whereas the kidney showed discoloration in two animals. These observations, however, were not associated with histological abnormalities. Hematological analysis revealed a dose-dependent reduction in platelet and reticulocyte counts, with statistically significant changes at 10 and 15 mg/kg (Supplementary Table 8). Clinical chemistry revealed elevated blood urea nitrogen (BUN) and creatinine levels, which were significant at 15 mg/kg, although without corresponding renal pathology (Supplementary Table 9). Coagulation parameters remained within normal limits (Supplementary Table 10). On the basis of these findings, the MTD was identified as 15 mg/kg (equivalent to ~2.43 mg/kg in humans), the lowest observed adverse effect level (LOAEL) was 10 mg/kg (~1.62 mg/kg HED), and the no observed adverse effect level (NOAEL) was 5 mg/kg (~0.81 mg/kg HED). Based on the efficacy doses observed in mice ranging from 0.1–2 µmol/kg bw (0.0902–1.804 mg/kg bw), the projected human equivalent doses are approximately 0.007–0.15 mg/kg bw, indicating a broad safety margin for VT-109.

Pharmacokinetic studies following a single intravenous injection of VT-109 in male rats revealed rapid plasma distribution, with peak concentrations (Cmax) observed within 2 minutes of dosing **(**Supplementary Tables 11, 12). Plasma pharmacokinetics revealed a Cmax of 40,548 ng/mL at 0.033 hr and an AUC₀–∞ of 27,908 hr·ng/mL **(**Supplementary Table 12**)**. In lung tissue, VT‑109 reached a Cmax of 3606 ng/g at 2 hours and an AUC₀–∞ of 12,406 hr·ng/g **(**Supplementary Table 13). Notably, substantial amounts of VT-109 persisted in the lung for at least 24 hours post dose, and a considerable proportion of the peak levels were retained. These findings indicate that VT-109 displays rapid systemic distribution, sustained pulmonary exposure, and pharmacokinetic properties that support its further development as a lung-targeted therapy.

## Discussion

In this study, through a series of iterative optimizations, we designed a synthetic cyclic inhibitor of EB3 with drug-like characteristics and improved physicochemical and biochemical properties. These optimizations, guided by NMR, were performed through the substitution of proteinogenic (natural) to nonproteinogenic (synthetic) amino acids and rigidification by backbone cyclization. In our preclinical studies, VT-109 showed marked stability and was effective in blocking pulmonary vascular leakage associated with a wide range of pathophysiological conditions, including polymicrobial sepsis and SARS-CoV-2 infection. Mechanistically, VT-109 blocked the NFAT and NFκB inflammatory pathways while simultaneously activating the FOXM1 regenerative program in endothelial cells. This shift from an inflammatory lung environment to a reparative state led to the prompt reannealing of VE-cadherin junctions, restoring tissue–fluid and immune homeostasis as well as repairing the lung architecture and function. The multifaceted therapeutic effects of VT-109 underscore its broad-spectrum therapeutic potential, addressing the urgent unmet need for effective interventions in ARDS (Fig. 7j).

The hallmark feature of ARDS is disruption of the endothelial barrier, which causes pulmonary edema^64^. The findings presented here further emphasize the pivotal role of the microtubule cytoskeleton in endothelial cells as a major regulator of pulmonary vascular leakage in ARDS^65^. The causal relationship between the microtubule cytoskeleton and endothelial dysfunction has previously been highlighted in the context of redox signaling^66^. In response to pathogens, microtubule-dependent activation of Rho, p38, and NF-κB signaling ultimately leads to vascular inflammation and barrier breakdown^67^. Furthermore, studies have shown that reorganization of the microtubule cytoskeleton is the major event leading to lung inflammation and injury caused by LPS or bacterial infection^68^ and that microtubule destabilizing agents such as sabizabulin reduce mortality from COVID-19^69^. While sabizabulin crosslinks α- and β-tubulin subunits, broadly inhibiting microtubule polymerization in multiple cell types^69^, VT-109 selectively targets microtubule-dependent pathological calcium signaling in activated endothelial cells without disrupting the microtubule cytoskeleton itself^13^.

Pulmonary microvascular endothelial cells predominantly express IP₃R3, with relatively lower levels of IP₃R2 and minimal to no IP₃R1^11^. This expression profile differs from that of peripheral endothelial cells, where the other isoforms are more prevalent depending on the vascular bed and species^70–74^. Compared with IP₃R1 and IP₃R2, IP₃R3 is also less sensitive to IP₃^75–77^ and appears to preferentially engage under proinflammatory conditions, where high levels of ligands are produced^11,12,78,79^. EB3, which is localized at the growing microtubule plus ends, establishes transient interactions with IP₃R3 and, as a result, promotes IP_3_R3 clustering^11^. We showed that targeting this process with a cognate peptide inhibits IP_3_R3 clustering and the resulting calcium release from IP_3_R3 stores^11–13^. By precisely targeting a disease-specific process, calcium release from endoplasmic reticulum stores, which occurs during endothelial inflammation and injury^11,12^, VT-109 achieves exceptional specificity with minimal toxicity. Our data show that VT-109 shifts endothelial injury toward endothelial repair by disrupting EB3 interactions with IP_3_R3, thereby blocking IP_3_R3 receptor clustering and limiting pathological calcium signaling. These effects of VT-109 are highly specific to endothelial cells, as the therapeutic benefits are lost in the absence of the endothelial transcription factor FOXM1. These findings indicate that VT-109 acts primarily through activation of the FOXM1-driven regenerative program. On the basis of the data presented here and building upon our previous work^12,13^, the use of VT-109 might offer a synergistic and comprehensive approach to address the complex pathophysiology of ARDS when supplemented with antibiotics, antivirals, or anti-inflammatory agents.

ARDS is a multifarious syndrome driven by uncontrolled inflammation and tissue damage^1^. It may result from direct insults to the alveolar epithelium, such as ventilator-induced lung injury or viral and bacterial infections, or from indirect mechanisms, such as systemic inflammatory responses occurring in sepsis, which lead to pulmonary endothelial barrier damage. Our findings highlight a central role for pulmonary edema in the pathogenesis of ARDS, irrespective of the initial cause of injury. VT-109 effectively mitigates vascular leakage and restores lung function by accelerating lung regeneration, underscoring its therapeutic potential. Importantly, our data suggest that VT-109 does not directly modulate immune cell responses. Instead, its beneficial effects likely stem from restoring endothelial barrier integrity, thereby reducing vascular inflammation and reestablishing tissue‒fluid homeostasis. The resolution of lung and systemic inflammation appears to be a secondary effect of these endothelium-specific actions. While the recruitment of immune cells into infected lungs aims to clear pathogens^80^, the uncontrolled infiltration of neutrophils can further exacerbate injury through the excessive production of reactive oxygen species^81^ and inflammatory cytokines^82^, and the formation of neutrophil extracellular traps^83^. Our previous study revealed that the restrictive endothelial barrier itself serves as a regulatory checkpoint of neutrophil infiltration^3^. It not only limits the number of transmigrating neutrophils into the lung tissue^3^ but also induces transcriptomic changes in neutrophils during the transmigration process, enhancing their bactericidal function^3^. Our data support these findings. We showed that VT-109 restores VE-cadherin junctions and limits neutrophil recruitment into lung tissue without compromising antimicrobial defense. Furthermore, we demonstrated that the most efficacious dose of VT-109 (2 µmol/kg bw) reduced bacterial counts in the lungs but not in the blood or peritoneal fluid of septic mice. While VT-109 does not possess direct bactericidal properties, its effects are likely mediated through the restoration of the endothelial barrier and normalization of the immune responses of resident cells and neutrophils. These findings suggest that VT-109 may indirectly modulate immune defense responses, thereby contributing to the reestablishment of immune homeostasis in the lung.

The resolution of vascular injury in the lungs is critically dependent on the transcription factor FOXM1, which promotes both cell cycle progression and reannealing of VE-cadherin junctions through the upregulation of β-catenin^17^. During the repair phase, FOXM1 expression is induced at the transcriptional level^18^. Our study demonstrated that VT-109 activated the FOXM1-driven reparative program in pulmonary endothelial cells without altering FOXM1 mRNA levels, suggesting a posttranscriptional mechanism of activation. Consistently, VT-109 was ineffective in mice lacking endothelial *Foxm1*, confirming the requirement of this pathway for its therapeutic benefit.

FOXM1 transcriptional activity is regulated by polo-like kinase 1 (PLK1) through the phosphorylation of Ser715^84^. The findings presented in this work suggest that calcium signaling may interfere with PLK1-mediated FOXM1 activation. Specifically, phosphorylation of FOXM1 at Ser715 was markedly reduced in the lungs of septic mice, and these changes were reversed by VT-109 treatment. In support of this mechanism, calcium-dependent calcium- and integrin-binding protein 1 (CIB1) forms a complex with PLK1 in response to elevated intracellular calcium, potentially sequestering PLK1 and inhibiting FOXM1 phosphorylation under inflammatory conditions^84^. By disrupting aberrant calcium signaling, VT-109 likely promotes PLK1-dependent FOXM1 phosphorylation, enabling the expression of downstream genes critical for endothelial repair. This notion is further supported by the sustained expression of FOXM1 target genes and active proliferation of endothelial cells during peak injury in VT-109–treated animals, indicating that a reparative program is maintained despite ongoing inflammatory stress. Although the effects of VT-109 were not specifically evaluated across different age groups, a limitation of the study, we posit that its efficacy should not vary significantly with age, as VT-109 targets FOXM1 activity through posttranslational modification rather than regulation of the *Foxm1* gene, which decreases with age owing to changes in chromosome structure^85^. Collectively, these findings suggest that VT-109 reduces vascular leakage by preserving FOXM1 transcriptional activity in endothelial cells. Through this endothelial-specific mechanism, VT-109 shifts the balance from injury-driven signaling to regenerative responses, thereby accelerating the resolution of lung injury in ARDS.

Interestingly, our findings revealed that VT-109 effectively restored lung architecture and function. Previous studies have elucidated the intricate crosstalk between the pulmonary endothelium and epithelium, which plays a significant role in lung repair^86,87^. Notably, the pulmonary capillary endothelium acts as a supporting niche that promotes epithelial regeneration by secreting humoral factors, bioactive lipids, and matrix metallopeptidases. Specifically, matrix metallopeptidase 14 (MMP14) facilitates the generation of epidermal growth factor (EGF)-like fragments crucial for epithelial cell uptake and subsequent alveolar regeneration^88^. The autocrine signaling from endothelial cells via vascular endothelial growth factors (VEGFs) and fibroblast growth factors (FGFs) triggers MMP14 production^88^, and these growth factors are also taken up by epithelial cells, inducing the transdifferentiation of AT2 to AT1 cells^89^. Furthermore, sphingosine-1-phosphate (S1P) released from pulmonary endothelial cells after *Pseudomonas aeruginosa* injury is taken up by S1PR2 in AT2 cells to promote AT2 to AT1 cell transition^87^. These “transitioning AT2” cells, which play a vital role in epithelial barrier repair, are referred to by various authors as prealveolar type-1 transitional cell state (PATS)^90^, alveolar differentiation intermediate cells (ADI)^60^, damage-associated transient progenitors (DATP)^91^, or alveolar type 0 cells (AT0)^92^. Interestingly, the FOXM1 reparative program directly promotes the expression of VEGF by the endothelium^93^. Consistent with these findings, our data indicate that the benefits of VT-109 on alveolar epithelial repair are largely indirect. While VT-109 does not alter calcium signaling in AT2 cells, its stabilization of the endothelial barrier creates a supportive microenvironment that promotes epithelial cell survival and regeneration. Our data unequivocally demonstrate that the restoration of tissue‒fluid homeostasis precedes and accelerates lung regeneration by promoting efficient transdifferentiation of AT2 cells into AT1 cells. In essence, our findings highlight the pivotal role of the endothelial barrier in priming the intricate cellular processes that lead to accelerated recovery from acute lung injury.

While our findings underscore the therapeutic potential of VT-109 across multiple preclinical models of ARDS, several limitations warrant consideration. The murine ARDS models used in this study, although mechanistically informative, do not fully recapitulate the complexity and heterogeneity of human ARDS. To enhance the translational relevance of this work, future studies in large animal models, such as nonhuman primates, could offer a more robust basis for predicting clinical outcomes. A key difference between human ARDS caused by viral pneumonia and the current mouse models lies in the presence of viremia, which has been reported in approximately 34% of severely and critically ill COVID-19 patients^94^. In contrast, viremia is rarely observed in widely used murine models, including K18-hACE2 mice infected with the SARS-CoV-2 WA1/2020 strain and in the MA10 model. As such, our study could not address whether VT-109 has any impact on viral dissemination in the bloodstream, an important consideration in the context of human disease. Furthermore, aside from the brain in the K18-hACE2 mice, viral dissemination to peripheral organs was not observed in our models. This finding is consistent with prior findings that the SARS-CoV-2 WA1/2020 strain can access the central nervous system via direct neuronal entry in this transgenic model^63^. However, such neurotropism may not fully reflect the viral dissemination patterns observed in patients.

As we did not observe any effect of VT-109 treatment on viral mRNA levels in brain tissue or other organs across the viral pneumonia models used, we acknowledge that species-specific differences in viral dynamics between mice and humans may limit the translatability of our findings. These results may seem counterintuitive given that calcium signaling pathways, particularly those mediated by the TPC2, TRPML2, TRPML3, and Orai1 channels, have been implicated in SARS-CoV-2 endocytosis in endothelial cells^95^. However, these calcium entry routes are primarily activated through mechanisms that are independent of IP_3_R3-mediated calcium release, the pathway specifically targeted by VT-109. Therefore, the absence of detectable viremia or systemic viral dissemination in murine models may not accurately represent the clinical progression of viral infections such as severe COVID-19 in humans. If VT-109 is to be evaluated in the context of COVID-19 or other viral pneumonias, careful quantification of the viral load in BALF and blood will be necessary to assess any potential effects on viral dissemination and clearance.

The second limitation pertains to the indirect and largely unexplored effects of VT-109 on various nonendothelial cell types in the lung. While the impact of VT-109 on endothelial cells has been thoroughly investigated, its influence on other critical pulmonary cell populations, such as epithelial cells, pericytes, fibroblasts, alveolar macrophages, and neutrophils, remains largely unknown. Future studies utilizing advanced methodologies such as single-cell transcriptomics or multiomics approaches will be instrumental in delineating the broader mechanism of action and cellular specificity of VT-109 within the lung microenvironment. Two key mechanistic points are important to highlight in this respect. First, VT-109 selectively targets pathological calcium release mediated by IP_3_R3-regulated intracellular stores. This specificity distinguishes it from agents that affect calcium influx or release via other isoforms, such as IP_3_R1 or alternative calcium channels, as previously reported^11^. Second, IP_3_R3 is the isoform that is least sensitive to IP_3_ stimulation, allowing VT-109 to block aberrant calcium signaling without disrupting the physiological calcium handling required for normal endothelial cell function.

The third limitation of our study is that VT-109 was not tested in models of Gram-positive bacterial pneumonia, such as *Streptococcus pneumoniae* and *Staphylococcus aureus*^96^. These pathogens are among the most common causes of community-acquired pneumonia and disrupt the alveolar‒capillary barrier through pore-forming toxins, such as pneumolysin, which promote Ca^2+^ influx via a mechanism independent of EB3-IP_3_R3-mediated signaling^97,98^. Whether VT-109 can mitigate vascular leakage in this context remains unknown, and future studies will be needed to determine its efficacy against these alternative lung injury pathways.

While our study established a broad safety margin for VT-109 in escalating-dose toxicity studies in rats, a comprehensive toxicological evaluation remains necessary. In the non-GLP single-dose study reported here, the maximum tolerated dose (MTD) was 15 mg/kg bw (equivalent to 30 mg/kg bw in mice), and the no observed adverse effect level (NOAEL) was 5 mg/kg bw (equivalent to 10 mg/kg bw in mice). All therapeutic doses used in all our efficacy studies fell well below this threshold, supporting a favorable therapeutic window. However, these preliminary studies do not address potential off-target effects, repeat-dose safety, or tissue-specific toxicity. These limitations highlight the need for future GLP-compliant safety studies to fully establish the toxicological profile of VT-109 prior to clinical translation.

In summary, our preclinical studies established compelling evidence for the therapeutic efficacy of VT-109, a synthetic cyclic EB3 inhibitor, in murine models of ARDS. VT-109 showed significant therapeutic effects in diverse ARDS models induced by endotoxin, polymicrobial sepsis, mechanical ventilation, *P. aeruginosa*, and SARS-CoV-2 infection. The effects of VT-109 on inflamed lung tissues are multifaced. The data presented here strongly suggest that VT-109 shifts the balance from injury-driven inflammation to endothelial repair by stabilizing the vascular barrier and activating the FOXM1 reparative program in endothelial cells. As a result, VT-109 effectively promotes reannealing of VE-cadherin junctions, restores tissue‒fluid homeostasis, and limits excessive neutrophil-driven inflammation, supporting the recovery of lung architecture and function. Ultimately, VT-109 holds substantial promise as a broad-spectrum therapeutic intervention against vascular leakage, making it a strong candidate for further clinical testing.

## Materials and methods

### Experimental design

This study aimed to develop an EB3 inhibitor with drug-like physicochemical properties for the treatment of ARDS. A total of 61 compounds, including linear, cyclic, and stapled analogs, were designed and synthesized based on the parent compound EBIN. STD-NMR and HSQC-NMR were employed as primary screening methods to measure each compound’s binding affinity to EB3. The solubility, lipophilicity, aggregation potential, and inhibitory ability of each compound were also determined. Compounds with promising profiles were further characterized in cell culture models. Fluorescence live-cell imaging using a FAM-labeled analog was performed to evaluate the cell-penetrating ability of the peptides. Additionally, stability in human, dog, rat, and mouse plasma, along with storage stability, was determined prior to selecting the lead and backup compounds VT-109 and VT-108, respectively. The selected compounds were tested for their IC_50_ values in inhibiting calcium release from ER stores via Fluo-4 live-cell fluorescence imaging. Their effects on endothelial barrier function were further evaluated by measuring the TEER and vascular leakage of plasma proteins in response to PAR-1 activation. Furthermore, the efficacy of VT-109 was evaluated in multiple models of ARDS, including systemic endotoxemia induced by systemic LPS exposure, polymicrobial sepsis induced via CLP surgery, VILI, a two-hit model involving CLP followed by mechanical ventilation, and *Pseudomonas aeruginosa*-induced pneumonia.

The efficacy of VT-109 in treating diffuse alveolar damage was also evaluated in two models of SARS-CoV-2-induced pneumonia: K18-hACE2 transgenic mice infected with the Washington strain of SARS-CoV-2 and BALB/c mice infected with the mouse-adapted SARS-CoV-2-MA10 virus. Pulmonary vascular permeability was measured via the EBAE assay, whereas pulmonary edema was determined via the lung wet‒dry weight ratio. The VE-cadherin junctions were evaluated via co-immunofluorescence staining with PECAM-1 and analyzed as overlapping junctional areas as we previously published^99^. Lung inflammation was assessed by measuring MPO levels, scoring the number of neutrophils in lung tissues via histological staining with an anti-Ly6G antibody, analyzing immune cells in BALF via cell smears stained with Diff-Quik, quantifying proinflammatory cytokines in BALF, and assessing NFAT nuclear localization.

Lung injury was evaluated via histopathological analysis and ALI scoring, whereas lung function was assessed by measuring lung compliance. Multiorgan function was assessed through a blood chemistry panel and 7-day survival rates. Immunostaining for AT2-AT1 transition markers was performed to determine the impact of VT-109 on epithelial cell repair. To delineate the mechanism of action of VT-109, bulk RNA-seq was conducted on isolated pulmonary endothelial cells, and gene expression and pathway alterations were analyzed via bioinformatic approaches.

Western blotting was performed to determine changes in the protein expression levels of FOXM1 target genes. Additionally, an endothelial cell-specific deletion of the *foxm1* gene was used to evaluate whether VT-109 can prevent vascular leakage in mice lacking FOXM1. Safety assessments included measurements of bacterial burden in the CLP model and i.t. model of *Pseudomonas aeruginosa*-induced pneumonia, as well as viral replication in the SARS-CoV-2 model, via PCR and plaque assays. All immunostaining, microscopy, imaging, quantification, and histology scoring procedures were performed in a blinded manner to the experimental group and genotype, where applicable. A minimum of three biological replicates was included for each experiment.

### Mice

FOXM1^ΔiEC^ mice were generated by crossing FOXM1^fl/fl^ mice with Cdh5-Cre-ERT2 mice expressing inducible Cre under the Cdh5 (VE-cadherin) promoter. CD1 mice (Crl:CD1, #022, Charles River) were used in experiments involving the PAR-1 agonist peptide, endotoxin, CLP, HVMV, i.t. *P. aeruginosa*, and the two-hit model. Transgenic K18-hACE2 mice (B6.Cg-Tg(K18-ACE2)2Prlmn/J, #034860, The Jackson Laboratory) were used in the SARS-CoV-2-induced ARDS model. BALB/c mice (BALB/cJ, #000651, The Jackson Laboratory) were used in the SARS-CoV-2-MA10-induced ARDS model. All mice were housed under pathogen-free conditions with a 12-hour light‒dark cycle, controlled temperature (22 °C), and humidity, with free access to food and water. All the mice used in this study were purchased at 7–8 weeks of age and underwent a minimum of one week of acclimation prior to the experimental procedures. Animals between 8 and 13 weeks of age were included in the study. The experimental groups were randomly assigned and balanced for both age and sex, with blinding applied where feasible. Randomization was based on body weight to ensure comparability across groups. Animal studies conducted with BSL3 agents were housed in an ABSL3 environment.

All the animal studies were conducted under protocols approved by the Institutional Animal Care and Use Committee (IACUC) and the Institutional Biosafety Committee at UIC.

### Cell line

Human primary microvascular lung endothelial cells (HLMVECs; #ACBRI 468, Cell Systems) were obtained from Cell Systems and cultured in EGM-2 media (#CC-4176, Lonza) supplemented with an EGM2 Bulletkit (#CC-3162, Lonza) and 10% fetal bovine serum. The cells were cultured at 37 °C with 5% CO_2_. These cell lines were obtained from authenticated sources and confirmed to be free of mycoplasma.

### Nuclear magnetic resonance (NMR) spectroscopy

NMR saturation transfer difference (STD) experiments were carried out on a Bruker 800 MHz Avance spectrometer at 25 °C on 1 µM EB3 dissolved in phosphate buffered saline (pH 7.6) with or without 100 molar excesses of tested peptides. The difference spectrum was obtained by subtracting the spectra collected with saturation of 100 Hz at −1 ppm for 1 s from the spectra without saturation. Signal intensities were measured and used to calculate the amplification ratio (I_STD_/I_NO SAT_*{L}, where I_STD_ is the highest STD signal intensity measured with saturation, I_NO SAT_ - the intensity measured for the same signal without saturation, {L} – peptide concentration). The peptides with the amplification ratio exceeding that of the parent peptides were considered to have improved binding characteristics.

^1^H-^15^N HSQC spectra were collected on 50 μM ^15^N-labeld EB3_200-281_ dissolved in phosphate buffered saline with 10% D_2_O in the presence or absence of 50 μM VT-109 at 800 MHz. The spectra were processed and analyzed in NMRPipe^87^. The ^1^H and ^15^N chemical shift assignments were previously published^88^.

### Peptide synthesis

Linear peptides are synthesized using a stepwise solid-phase method by 9-fluorenylmethoxycarbonyl (Fmoc) chemistry on the Wang resin (AnaSpec) with a 12-channel multiplex peptide synthesizer (Protein Technologies) according to the manufacturer’s procedures. For the synthesis of peptides with -NH_2_, the Wang resin is replaced with a Rink amide resin. Detachment of peptides from the resin and removal of the side chain protection groups are done by incubating the resin with a mixture of trifloroacetic acid (TFA):Thioanisole:Water:Phenol:1,2-ethanedithio (82.5:5:5:5:2.5 v/v) for 2 hours.

The crude peptide is purified on a preparative Kinetex reversed-phase C18 column, 150 ×21.1 mm (Phenomenex, Torrance, CA, USA) using a BioCad Sprint (Applied Biosystems). A flow rate of 30 mL/min with solvent A (0.1% TFA in deionized water) and solvent B (0.1% TFA in acetonitrile) is used. The column is equilibrated with 5% solvent B before sample injection. Elution is performed with a linear gradient from 5% solvent B to 100% solvent B in 60 min. The absorbance of the column effluent is monitored at 214 nm, and peak fractions are pooled and lyophilized. The pure peptide fraction is identified by matrix-assisted laser desorption/ionization time-of-flight mass spectrometry (MALDI-TOF MS) or electrospray ionization mass spectrometry (ESI-MS) and lyophilized.

For cyclization of peptides by disulfide formation, linear peptides containing cysteine residues at the N- and C-terminus at 1 mg/ml in 50 mM NH4HCO3 are stirred overnight at 25 °C. Head-to-tail cyclization of the dried linear peptide between the COOH group of 7 and -NH_2_ group of 1 was carried out in solution. The crude linear peptide (300 mg) was dissolved in DCM (1 mg/mL) and DIEA (10 eq) and mixed with HOBt (1 equiv.) and HBTU (1 equiv.) in DMF (2 mL) at rt. The reaction was monitored by mass spectrometry and completed within 2 hours. The solution was concentrated by rotary evaporation in preparation for HPLC purification. For cyclization of peptides by Sortase (SrtA), linear peptides with the SrtA optimal cleavage site LPETGG at the C terminus and diglycine motif (GG) at the N terminus allows intramolecular ligation by SrtA to form a cyclic peptide. The intramolecular transpeptidation reaction is performed with 150 µM of the linear peptide and 5 µM SrtA in sortase reaction buffer (50 mM Tris, pH 7.5, containing 150 mM NaCl and 10 mM CaCl_2_) at 4 °C for 24 h. After completion, the reaction solution is dried, and the cyclic peptide is purified by HPLC. Cyclization is confirmed by HPLC and MS.

For the synthesis of N-myristoylated peptides, chloroform (2.5 ml) is added to ~250 mg of Myr-anhydride and mixed until Myr-anhydride is dissolved. 2.5 ml of dimethylformamide (DMF) is added following 50 µmole peptide on the resin. The mixture is kept at 60 °C for 1 hour; filtered and washed with 50 ml of hot chloroform:DMF (1:1).

For the synthesis of stapled peptides, the procedure is the same as L-form peptide synthesis except that Fmoc-L-Serine is replaced with Fmoc- O-allyl ether. L-Serine (Ser) and L-Homoserine (Hse) O-allyl ethers are selected as olefin-containing residues due to their ready availability and trivial derivatization as allyl ethers.

### Pharmacokinetic studies in rats

The pharmacokinetic profile of VT-109 was evaluated in male Crl:CD (SD) rats following a single intravenous (IV) slow bolus injection via the tail vein at a dose of 5 mg/kg. Blood samples were collected at predefined time points (2, 10, 30, 60, 90 minutes, and 2, 4, 6, 8, and 24 hours post-dosing) from subgroups of rats, ensuring each animal contributed a limited number of samples to minimize stress. Plasma was separated by centrifugation and stored at -80 °C until analysis. Lungs were collected at selected time points (0.5, 2, 8, and 24 hours) for drug quantification. Plasma and lung concentrations of VT-109 were measured using a validated liquid chromatography-tandem mass spectrometry (LC-MS/MS) method. Pharmacokinetic parameters, including Cmax, Tmax, AUC, half-life, clearance, and volume of distribution, were calculated using Phoenix WinNonlin software.

### Endotoxemia models

To induce a systemic inflammatory response with lipopolysaccharide (LPS), CD1 mice were preloaded intraperitoneally with corn oil (Sigma Aldrich) at 12 mL/kg body weight every 48 hours for three doses. LPS was administered on day 5 following the last corn oil injection. CD1 mice received a single intraperitoneal dose of LPS (4 mg/kg; *Escherichia coli* O55:B5, Santa Cruz Biotechnology). VT-109 was administered intravenously in two treatment protocols: the early treatment group received three daily doses of VT-109 starting 1 day after LPS injection, while the late treatment group received two daily doses starting 3 days after LPS injection.

In the survival studies, mice received i.p. corn oil at a dose of 0.35 mL two days before the challenge. CD1 mice were injected intraperitoneally with a lethal dose 90 (LD_90_) of LPS (Santa Cruz Biotechnology). In most experiments, this dose was 30 mg/kg bw; however, it was adjusted for each new batch of LPS in pilot studies. In the survival study, treatment with varying doses of VT-109 (1, 10, 100, 250, 500, and 2000 nmol/kg bw) was initiated 20 hours after the LPS challenge. In the lung compliance study, a non-efficacious dose (1 nmol/kg bw) of VT-109 was used as a control, while 250 nmol/kg bw of VT-109 was used for the experimental group. Both treatments were administered intravenously 20 hours after the LPS challenge. All mice received 1 mL of prewarmed saline daily for 3 days.

### Polymicrobial sepsis models

Polymicrobial sepsis was induced in CD1 mice via cecal ligation and puncture (CLP) surgery. After a midline incision, the cecum was exteriorized, ligated, and punctured twice with an 18- or 20-gauge needle to allow fecal matter to enter the peritoneal cavity. Treatment groups included VT-109 monotherapy (2 µmol/kg bw, administered intravenously), antibiotic monotherapy (Enrofloxacin, 5 mg/kg bw, administered intraperitoneally at 8-, 24-, and 28-hours post-CLP), or combination therapy with VT-109 and antibiotics. Mice that underwent sham surgery without ligation or puncture of the cecum were used as controls. Vehicle-treated mice served as treatment controls and followed the same treatment schedule as the VT-109 group. All mice received daily injections of 1 mL of warmed saline for 3 days.

### High tidal volume mechanical ventilation

Mice intubated with a 20-gauge catheter were ventilated using a Harvard Apparatus ventilator (Harvard Bioscience) with high tidal volumes of 40 mL/kg body weight, a respiratory rate of 80 breaths per minute, and no positive end-expiratory pressure (PEEP) for 2 hours to induce ventilator-induced lung injury (VILI). Intravenous treatment with 250 nmol/kg bw VT-109 was administered at the onset of HVMV. Mice breathing spontaneously were used as experimental control.

### Two-hit model of ARDs

In the two-hit model of ARDS, CD-1 mice underwent CLP surgery using a 20-gauge needle. After 20 hours, the mice were subjected to normal tidal volume mechanical ventilation at 10 mL/kg body weight and a respiratory rate of 80 breaths per minute for 5 hours using a Harvard Apparatus ventilator (Harvard Bioscience). Intravenous treatment with 250 nmol/kg bw VT-109 was administered at the onset of MV. Sham mice were used as experimental controls, and vehicle-treated mice were used as treatment controls. All mice received 1 mL of prewarmed saline before being placed on the mechanical ventilator.

### I.t. P. aeruginosa-induced pneumonia

Mice (CD1) were anesthetized by intraperitoneal injection of xylazine and positioned supine on a sterile surgical surface. A small midline incision was made in the neck to expose the trachea. GFP-expressing Pseudomonas aeruginosa (PA) was cultured overnight in LB broth supplemented with carbenicillin (100 µg/mL). A total volume of 30 µL PBS containing 0.5 × 10⁶ colony-forming units (CFU) was injected directly into the trachea using a sterile 28G insulin syringe. Following intratracheal instillation, mice received VT-109 (250 nmol/kg body weight) or vehicle by IV at 5- and 24-hours post-infection. Lung tissues were collected at 24 and 48 hours to assess treatment effects. The left lung was inflated with fixative and harvested for histological analysis, while the right lung was collected for bacterial quantification. For CFU analysis, the right lung was homogenized in 1 mL of sterile PBS using a bead-based tissue homogenizer. Homogenates were serially diluted and plated on LB agar containing carbenicillin (100 µg/mL). Plates were incubated overnight at 37 °C, and CFUs were counted the following day. Bacterial counts were normalized to the wet weight of the corresponding lung tissue.

### SARS-CoV-2-MA10-induced pneumonia

BALB/c mice were inoculated intranasally with 1 × 10⁴ plaque-forming units (PFU) of the mouse-adapted SARS-CoV-2-MA10 virus in 50 µL of sterile PBS. VT-109 (250 nmol/kg bw) was administered intravenously starting on day 1 post-infection for 3 consecutive days, once daily. All mice received 500 µL of prewarmed saline once daily for 3 days.

### Diffuse alveolar damage induced by SARS-CoV-2 (WA/2020)

The transgenic K18-hACE2 mice were inoculated intranasally with 1 × 10⁴ plaque-forming units (PFU) of the Washington strain of SARS-CoV-2 (WA/2020) in 50 µL of sterile PBS. In the early treatment group, VT-109 (100 or 250 nmol/kg bw) was administered intravenously starting on day 1 post-infection for 3 consecutive days, once daily. In the late treatment group, VT-109 (250 nmol/kg bw) was also administered intravenously starting on day 3 post-infection for 3 doses, once daily. For the inflammatory cytokine analysis, 2 µmol/kg bw VT-109 was used, with the treatment schedule involving both the early and late treatment groups. All mice received 500 µL of prewarmed saline once daily for 3 days.

### Statistical analysis

Statistical significance was calculated via GraphPad Prism unless otherwise indicated. The statistical details of each experiment are provided in the figure legends. All the data are shown as the means ± SEMs unless otherwise indicated. Normality was tested across all datasets. A two-tailed Student’s *t*-test was applied when two groups were compared. One-way ANOVA was used for comparing multiple groups, with two-way ANOVA applied where applicable. Post hoc multiple comparison tests were performed to identify significant differences between groups. Q and *P* < 0.05 were considered statistically significant where applicable. Nonparametric statistical tests were used for nonnormally distributed data. All the data were tested for outliers and removed from the analysis via ROUT’s method. To ensure adequate power, data were collected from three or more independent experiments in triplicate. The sample size for each experiment was determined on the basis of commonly used values in similar or previous experiments unless noted otherwise. Across all mouse models, age, sex, and weight were comparable. All immunostaining, microscopy, imaging, quantification, and histology scoring procedures were performed in a blinded manner to the experimental group and genotype, where applicable.

## Supplementary information

Supplementary Materials

## Data Availability

Data, materials, and methods related to this study are presented in the main manuscript or the Supplementary Materials. The bulk RNA-sequencing data were submitted to the Gene Expression Omnibus under the reference number GSE 282723. Any additional information or requests for resources and materials will be addressed and fulfilled by the corresponding author, Dr. Yulia A Komarova (ykomarov@uic.edu).
