# Supplementary material for: Therapeutic targeting of endothelial calcium signaling accelerates the resolution of lung injury
Source: Signal Transduct Target Ther. 2025 Nov 18;10:375. doi: 10.1038/s41392-025-02461-y (PMC12627862; doi:10.1038/s41392-025-02461-y)
Supplement: Supplementary file 1 — Supplementary Materials [file 41392_2025_2461_MOESM1_ESM.docx]

Supplementary Materials for

**Therapeutic targeting of endothelial calcium signaling accelerates resolution of lung injury**

Wan Ching Chan, Man Long Kwok, Xinyan Qu, Hazem Abdelkarim, Jonathan Le, Deying Yang, Avik Banerjee, Shuangping Zhao, Jacob Class, Marlen Gonzalez, Harry Hailemeskel, Raman Ghotra Singh, Ricardo Gallardo-Macias, Vadim J. Gurvich, Mark Maienschein-Cline, Matthew Lindeblad, Kasim Kabirov, Alex Lyubimov, Patrick Belvitch, Justin Richner, Vadim Gaponenko, Yulia A Komarova

Corresponding Author: Dr. Yulia A Komarova, [ykomarov@uic.edu](mailto:ykomarov@uic.edu)

**This PDF file includes:**

The ARRIVE Guidelines 2.0

Materials and Methods

Supplementary Figures 1 to 7

Supplementary Tables 1 to 13

**The ARRIVE Guidelines 2.0**

*Study design*

This study aimed to develop an EB3 inhibitor with drug-like physiochemical properties for the treatment of ARDS. A total of 61 compounds, including linear; cyclic; and stapled analogs, were designed and synthesized based on the parent compound, EBIN. STD-NMR and HSQC-NMR were employed as primary screening methods to measure each compound’s binding affinity to EB3. The solubility, lipophilicity, aggregation potential, and inhibitory ability of each compound were also determined. Compounds with promising profiles were further characterized in cell culture models. Fluorescence live-cell imaging using FAM-labeled analog was performed to evaluate cell-penetrating capabilities. Additionally, stability in human, dog, rat, and mouse plasma, along with storage stability, were determined prior to selecting the lead and backup compounds, VT-109 and -108, respectively. The selected compounds were tested for their IC_50_ values in inhibiting calcium release from ER stores using Fluo-4 live-cell fluorescence imaging. Their effects on endothelial barrier function were further evaluated by measuring TEER and vascular leakage to plasma proteins in response to PAR-1 activation. Furthermore, the efficacy of VT-109 was evaluated in multiple models of ARDS, including systemic endotoxemia induced by systemic LPS exposure, polymicrobial sepsis using CLP surgery, VILI, a two-hit model involving CLP followed by mechanical ventilation, and *Pseudomonas aeruginosa*-induced pneumonia.

The efficacy of VT-109 in treating diffuse alveolar damage was also evaluated in two models of SARS-CoV-2-induced pneumonia: K18-hACE2 transgenic mice infected with the Washington strain of SARS-CoV-2, and BALB/c mice with the mouse-adapted SARS-CoV-2-MA10 virus. Pulmonary vascular permeability was measured by EBAE assay while pulmonary edema was determined using the lung wet-to-dry weight ratio. The VE-cadherin junctions were evaluated via co-immunofluorescent staining with PECAM-1 and analyzed as overlapping junctional area as we previously published^87^. Lung inflammation was assessed by measuring MPO levels, scoring of the neutrophil numbers in lung tissues using histological staining with anti-Ly6G antibody, analyzing immune cells in BAL fluids using cell smears stained with Diff-Quik, quantifying proinflammatory cytokines in BAL, and assessing NFAT nuclear localization.

Lung injury was evaluated using histopathological analysis and ALI scoring, while lung function was assessed by measuring lung compliance. Multi-organ function was assessed through a blood chemistry panel and 7-day survival rates. Immunostaining for the AT2-AT1 transition markers was performed to determine the impact of VT-109 on epithelial cell repair. To delineate the mechanism of action of VT-109, bulk RNA-seq was conducted on isolated pulmonary endothelial cells, and gene expression and pathway alterations were analyzed using bioinformatic approaches.

Western blotting was performed to determine changes in the protein expression levels of FOXM1 target genes. Additionally, an endothelial cell-specific deletion of *foxm1* gene was used to evaluate whether VT-109 treats vascular leakage in mice lacking FOXM1. Safety assessments included measurements of bacterial burden in the CLP and i.t. model of *Pseudomonas aeruginosa*-induced pneumonia, as well as viral replication in the SARS-CoV-2 model, using PCR and plaque assays. All immunostaining, microscopy, imaging, quantification, and histology scoring were performed blinded to the experimental group and genotype, where applicable. A minimum of three biological replicates were included for each experiment.

*Sample size*

The exact number of experimental units for each experiment can be found in the figure legends of each corresponding experiment. The sample size for mouse experiments was determined based on prior studies using similar models and outcome measurements. A minimum of three mice per group was used to allow for initial statistical comparisons, with sample sizes estimated to provide at least 80% power to detect statistically significant differences (α = 0.05) using two-tailed tests. Group sizes were further adjusted to account for potential biological variability and occasional technical loss due to procedural complications or suboptimal tissue quality.

*Inclusion and exclusion criteria*

All data were tested for outliers and removed from the analysis using ROUT’s method. No criteria were set for including and excluding animals during the experiment. Please see the corresponding figure legends from each experiment for the exact values of n in each group.

*Randomisation*

Most of the mice used in the experiments were purchased from an external vendor. In each experiment, mice from multiple vendor-supplied lots were included, and they were randomly caged by BRL staff. Group assignments were made based on cage numbers, without prior observation of the animals, to avoid selection bias. For in-house bred mice, group allocation was based on similar age to ensure consistency. Most treatments and measurements were completed within approximately one hour to minimize potential confounding factors related to time.

*Blinding*

Investigators could not be blinded to the different mice group during the experiments due to institutional policy that we must clarify which cage has been subjected to its specific treatment or surgical procedure. All investigators handled the mice cannot avoid seeing which group the mice were in. Blinding was done for acute lung scoring, where sample IDs were randomized before scoring.

*Statistical methods*

Statistical significance was calculated using GraphPad Prism unless otherwise indicated. Statistical details of each experiment are provided in the figure legends. All data are shown as mean ± SEM unless otherwise indicated. Normality was tested across all datasets. A two-tailed Student’s *t*-test was applied when comparing two groups. One-way ANOVA was used for comparing multiple groups, with two-way ANOVA applied where applicable. Post hoc multiple comparison tests were performed to identify significant differences between groups. Q and P < 0.05 were considered statistically significant, where applicable. Non-parametric statistical tests were used for non-normal data. All data were tested for outliers and removed from the analysis using ROUT’s method. To ensure adequate power, data were collected from three or more independent experiments with triplicates. Sample size for each experiment was determined based on commonly used values in similar or previous experiments unless noted otherwise. In all models, mice were selected to be similar in age, sex, and weight. All immunostaining, microscopy, imaging, quantification, and histology scoring were performed blinded to the experimental group and genotype, where applicable.

*Experimental animals*

FOXM1^ΔiEC^ mice were generated by crossing the FOXM1^fl/fl^ mice with Cdh5-Cre-ERT2 mice expressing inducible Cre under Cdh5 (VE-cadherin) promoter. CD1 mice (Crl:CD1, #022, Charles River) were used in experiments involving the PAR-1 agonist peptide, endotoxin, CLP, HVMV, and the two-hit model. The transgenic K18-hACE2 mice (B6.Cg-Tg(K18-ACE2)2Prlmn/J, #034860, The Jackson Laboratory) were used in the SARS-CoV-2-induced ARDS model. The BALB/c mice (BALB/cJ, #000651, The Jackson Laboratory) were used in the SARS-CoV-2-MA10-induced ARDS model. Mice were housed in pathogen-free conditions with a 12-hour light-dark cycle, controlled temperature (22°C), and humidity, with free access to food and water. All mice used in this study were purchased at 7-8 weeks of age and underwent a minimum of one week of acclimation prior to experimental procedures. Animals were enrolled in study between 8 and 13 weeks of age. Experimental groups were randomly assigned and balanced for both age and sex, with blinding applied where feasible. Randomization was based on body weight to ensure comparability across groups. Animal studies conducted with BSL3 agents were housed under ABSL3 environment. All animal studies were conducted under approved protocols by the Institutional Animal Care and Use Committee (IACUC) and Institutional Biosafety Committee at UIC.

*Experimental procedures*

Please see our Supplemental Materials for all experimental procedures related to this manuscript.

*Results*

All related summary statistics for each experiment group for each experiment can be found in the corresponding figure legend.

**Materials and Methods**

***Molecular docking studies***. The binding of VT-109 with EB3’s C-terminus (residues 200–281) was modeled using AutoDock Vina^25^. Ten models were generated and assessed based on NMR experiments to select the best binding pose. Using Molecular operating environment (MOE) software, a 2D map was generated to show the binding interface between VT-109 and EB3.

***Lipophilicity of peptides.*** Two separate solutions were obtained: an aqueous phosphate buffer with saturated n-octanol, and an n-octanol solution with saturated phosphate buffer. Peptides were then dissolved and diluted in the phosphate buffer. Different partitions were then made with different n-octanol to water ratio before shaking for an hour at room temperature. The aqueous phase and n-octanol were allowed to equilibrate, and each phase was extracted and analyzed for peptide concentrations. The ratio of peptide concentration in n-octanol to that in water is expressed as *log(P)*.

***Stability assay of peptides in blood plasma of rodents, dogs, and humans.*** For the plasma stability assay, 5 μM of each peptide was incubated with species-specific plasma for 120 minutes and samples were taken at the required time points. Positive control used were molecules with known stability: enalapril, propantheline, and warfarin. The amount of test compound remaining at each time point was analyzed by LC-MS/MS. Appropriate control compounds for each species were also incubated.

***Stability assay of peptides in various storage conditions.*** Peptides were dissolved and stored in phosphate-buffered sterile saline at -20, 4, 37, and 60°C for 8 days. The amount of peptide remaining after the study was analyzed by LC-MS/MS.

***Acute toxicity studies in rats.*** For the acute toxicity evaluation, male Crl:CD (SD) rats received a single IV dose of VT-109, with 5% N,N-dimethylacetamide (DMA), 10% Polyethylene Glycol 400 and 1% Polysorbate 80 with sodium hydroxide to adjust pH to 6.5 – 7.0 in 0.9% sodium chloride, injected per day at 0 (Group 1), 3.75 (Group 2), 7 (Group 3), and 15 mg/kg (Group 4) for seven consecutive days. Animals were monitored for clinical signs, body weight changes, and mortality for three days post-administration. Blood was collected on Day 8 for coagulation, hematology, and clinical chemistry analysis. Parameters measured included prothrombin time, fibrinogen, leukocyte differentials, liver enzymes (ALT, AST, ALP), cholesterol, and other metabolic markers.

***Transendothelial electrical resistance (TEER) assay.*** TEER was performed to assay the endothelial barrier functions. Human pulmonary artery endothelial cells were plated onto gelatin-coated 8W1E gold electrodes (Applied Biophysics). Baseline TEER measurements were collected before the cells were incubated with 1 μM of peptide for an hour in media supplemented with 1% serum. Changes in TEER in response to 50 nM α-thrombin are monitored using an Electric Cell Substrate Impedance Sensing system (Applied Biophysics Inc.).

***Peptide uptake in endothelial cells.*** The rate of peptide uptake by primary human microvascular endothelial cells was measured using 5’6FAM (fluorescein)-labeled peptides and confocal fluorescence live-cell imaging, with fluorescein alone used as a negative control. Changes in intracellular fluorescence for each peptide were monitored over time, and saponin, a detergent, was added at the end of the experiment to induce full uptake of the tested peptides by endothelial cells. The fluorescence intensity inside the cells was tracked over time, and the half-time uptake (t1/2) and maximum uptake were calculated.

***Cytosolic calcium measurements.*** The Ca^2+^-sensitive dye Fluo-4 was used to measure the intracellular calcium concentration via a robotically integrated platform for high content screening (FlexStation-II, Molecular Devices). Primary human microvascular endothelial cells were grown on a 96-well microplate until confluency. The endothelial cells were then loaded with Fluo-4am (Life Technologies) for 20 minutes at 37 °C in culture media with supplements. Treatments with VT-108 and -109 were applied alongside the Fluo-4am. After incubation, endothelial cells were washed with HBSS before imaging. To increase cytosolic calcium, endothelial cells were stimulated with 50 nM α-thrombin. The Fluo-4 fluorescent intensities were measured at excitation λ = 494 nm and emission λ = 506 nm every 2 seconds. At least 5 different concentrations of each compound were tested. The relative changes in Fluo-4am fluorescence, area under the curve, were plotted as a function of compound concentration in a *log* scale and the EC_50_ was determined from the dose-response curve.

***AT2 Cell Isolation and Calcium Imaging Assay.*** Primary alveolar type II (AT2) cells were isolated from CD1 mice as described previously^78,89^. Cells were seeded into 96-well plates at a density of 10,000 cells per well and cultured for 2–3 days to allow adherence and recovery. Cells were then exposed to varying concentrations of VT-109 for 1 hour. Following treatment, cells were incubated with 3 µM Fluo-4 AM in Hanks' Balanced Salt Solution (HBSS) for 20 minutes at 37°C in the dark. After dye loading and washing, calcium influx was triggered by the addition of ATP (100 µM). Fluorescence intensity was measured the in real time using a fluorescence plate reader (excitation: 485 nm; emission: 520 nm) for up to 4 minutes after 1 minute trigger reagent added.

To quantify calcium influx, fluorescence signals were first normalized by subtracting the lowest value in that wells in 4-minute recording. To assess calcium activity, all excitation peaks observed during the 4-minute measurement were identified, and their amplitudes were summed. The total amplitude was then divided by the number of excitation to calculate the average excitation amplitude for each well. This metric reflects the overall calcium response intensity under each condition.

***PAR-1 agonist peptide-induced microvascular leakage in lung.*** C57BL/6 mice were first treated with 2 µmol/kg bw I.V. of VT-108 or VT-109 followed by the challenge with 10 mg/kg bw PAR-1 agonist peptide (AP) (TFLLRNPNDK-NH_2_). Mice treated with PAR-1 control peptide (FTLLRNPNDK-NH_2_) was used as negative control. For Evan’s blue albumin extravasation, Evans’s blue-labeled albumin was administered at the same time as the PAR-1 AP. Vehicle-treated mice were used as treatment control.

***Evans’ blue-labeled albumin extravasation.*** To evaluate vascular permeability in lung tissues, mice were injected intravenously with Evans' blue-labeled albumin (25 mg/kg) 30 minutes before the collection of lung tissues. The lungs were perfused via the right ventricle with warmed phosphate-buffered saline to remove intravascular dye, and the right atrium was excised to facilitate clearance. The perfused lungs were then excised, homogenized in N,N-dimethylformamide (Sigma-Aldrich), and centrifuged at 12,000 × g for 30 minutes. The optical density of the supernatant was measured spectrophotometrically at 620 nm (Evans Blue) and 740 nm (hemoglobin correction), with the dye content normalized to the wet weight of the lungs. Corrections for hemoglobin were made as follows: Corrected absorbance (Evans blue) = Absorbance (620) - (1.426 × Absorbance (740) + 0.03). A standard curve was used to determine the exact concentrations of Evans’ blue-labeled albumin in each sample.

***Lung wet-to-dry weight ratio.*** To evaluate pulmonary edema, mouse lungs were weighed before and after drying in a 60°C oven for 3 days. The lung wet-to-dry ratio was then calculated.

***Lung compliance measurements.*** The trachea was cannulated, followed by mechanical ventilation using the Flexivent small animal ventilator (Scientific Respiratory Equipment Inc.). After 5 minutes of stable ventilation, static compliance was measured at 10 cmH₂O on the expiratory limb of a pressure-volume (P-V) loop using the Salazar-Knowles equation: Cstatic = ΔV * ΔP * C_static_​ where ΔV is the change in lung volume and ΔP is the corresponding change in pressure. Static compliance was expressed as mL/cmH_2_O.

***Myeloperoxidase (MPO) measurements.*** Lungs were perfused with PBS, weighed, and frozen in liquid nitrogen. The samples were then stored at -80°C to allow all tissues to be processed at once. No tissue samples were stored at -80°C for more than 1 week. MPO levels in the samples were determined using the o-dianisidine-H₂O₂ technique following enzyme extraction with hexadecyl-trimethylammonium bromide (HTAB). The absorbance change was measured at 400 nm over a 3-minute period, and the data were expressed as ΔOD_460_/min/g lung tissue.

***Inflammatory cytokine analyses.*** BAL and serum samples were analyzed by Quansys Biosciences using a Q-Plex™ custom mouse multiplexed ELISA array. Briefly, samples were decontaminated before being removed from BSL3 containment when appropriate. The samples were diluted with the appropriate Quansys sample dilution buffer and analyzed at 1:2 and 1:20 dilutions. Polypropylene low-binding 96-well plates were used to prepare the standard curves and to analyze the samples. An image with a 270-second exposure was captured using a Q-View™ Imager LS driven by Q-View™ Software. Total light emission was measured and reported as pixel intensity units, calculated using Q-View™ Software. The following cytokines were analyzed, where applicable: mIL-1α, mIL-1β, mIL-3, mIL-4, mIL-5, mIL-6, mIL-10, mIL-12, mIL-17, mMCP-1, mIFNγ, mTNFα, mMIP-1α, mGM-CSF, mRANTES, and mKC.

***VT-109’s effect on cytokine release by immune cells in vitro.*** Freshly isolated splenocytes from CD1 mice were treated with 2 µg/mL LPS for 2 hours. Two different concentrations of VT-109 (200 nM and 1 µM) were tested, and the cells were treated concurrently with LPS. Untreated splenocytes served as the control. After the 2-hour incubation, MCP-1, TNF-α, KC, and IL-6 levels were measured in the growth media using mouse multiplexed ELISA array. Data are expressed as pg/mL.

***Western blotting.*** For measuring protein expression, samples were lysed with radioimmunoprecipitation assay (RIPA) buffer (Sigma-Aldrich) supplemented with protease and phosphatase inhibitor cocktails 1, 2, and 3 (Sigma-Aldrich). The total protein concentration of each sample was measured using the Pierce bicinchoninic acid (BCA) protein assay kit (Thermo Scientific) and prepared in Laemmli sample buffer (Bio-Rad). The prepared samples were then separated using SDS-PAGE and transferred to nitrocellulose membranes overnight at 4°C. After blocking with 5% BSA, the samples were incubated with primary antibodies at 4°C overnight for VE-cadherin (#sc-6458, Santa Cruz Biotechnology), FOXM1 (#sc-376471, Santa Cruz Biotechnology), FOXO1 (#ab179450, Abcam), E2F3 (#27615-1-AP, Proteintech), p21 (#ZRB1141, Sigma-Aldrich), and GAPDH (#60004-1-Ig, Proteintech). After washing, the samples were incubated with the appropriate horseradish peroxidase-conjugated secondary antibodies for 2 hours at room temperature before detection using electrochemiluminescence.

***Bacterial count assay.*** 24 hours after the CLP surgery, blood from mice was collected via direct cardiac puncture using sterile techniques. BAL was performed by flushing the lungs twice with 1 mL of sterile phosphate-buffered saline (PBS) per wash. For PF collection, the peritoneal cavity was opened, and 3 mL of sterile PBS was gently applied to wash the peritoneal space. The PF was then aspirated and collected for analysis. All samples were kept on ice and processed immediately for bacterial quantification. Serial dilutions of all samples were plated on agar plates containing 5% of sheep blood, which were then incubated overnight at 37°C. The resulting bacterial burden was assessed by colony counts the following day.

***qRT-PCR.*** The brain, lung, heart, and kidneys were dissected from infected mice and homogenized in PBS. RNA was extracted from the tissue homogenates using a 96-well Quick-RNA Isolation Kit (Zymo Research; R1025) according to the manufacturer's protocol. SARS-CoV-2 viral genomes were quantified using RT-qPCR with a TaqMan primer and probe set with the following sequences: Forward 5′ GAC CCC AAA ATC AGC GAA AT 3′, Reverse 5′ TCT GGT TAC TGC CAG TTG AAT CTG 3′, Probe 5′ ACC CCG CAT TAC GTT TGG TGG ACC 3 (Integrated DNA Technologies; 10006713). A SARS-CoV-2 copy number control was obtained from BEI (NR-52358) and used to interpolate and quantify SARS-CoV-2 genomes.

***Focus forming assay (FFA).*** Supernatant obtained from homogenized tissue samples of infected mice was serially diluted and added to Vero cell monolayers in 96-well plates. The virus was allowed to infect for 1 hour, with the plate being rocked every 15 minutes. Then, 125 µL of a 1:1 solution of 2× DMEM with 8% FBS and 2% methylcellulose was added to the cells. Plates were incubated for 24 hours and then fixed by adding 4% paraformaldehyde (PFA) or 10% formalin. Cells were incubated overnight at 4°C with a 1:15,000 dilution of anti-SARS-CoV-2 Guinea pig polyclonal antibody (BEI Resources; NR-10361). After a 5–15 minute incubation with a 1:5,000 dilution of horseradish peroxidase (HRP)–conjugated goat anti–Guinea pig IgG (Sigma-Aldrich), foci were detected by the addition of TrueBlue substrate (KPL). Foci were analyzed with a CTL Immunospot instrument.

***Tissues processing, embedding, and sectioning.*** At the end of the study, lung tissues were perfused-fixed with 10% neutral-buffered formalin. Tissue processing, embedding, and sectioning of murine lung samples were performed by UIC’s Research Histology Core. Briefly, following the fixation in 10% formalin, murine lung samples were loaded into ASP 300s automated tissue processor (Leica Biosystems) and dehydrated by a series of ascending graded ethanol, cleared in xylene, and infused with paraffin following a preset protocol. Samples were then embedded in paraffin (Paraplast, Leica Microsystem), and five micrometer sections were cut and mounted on positively charged slides before drying.

***Histological and immunohistochemical staining.*** The lung samples were stained with H&E by UIC’s Research Histology Core and imaged by UIC’s Research Tissue Imaging Core. Briefly, adhered sections were deparaffinized, stained with H&E or Ly6G (1:200, #87048, Cell Signaling Technology), and dehydrated on Leica Autostainer XL (Leica Biosystems) following a preset protocol. The staining with Ly6G was appropriately followed up by incubation in corresponding horseradish peroxidase secondary antibody and viewed with 3,3’-diaminobenzidine as the chromogenic substrate. Sections were mounted with Micromount media (Leica Biosystems) on CV5030 automated cover-slipper (Leica Biosystems). Stained slides were scanned at 40X magnification on Aperio AT2 brightfield digital scanner (Leica Microsystems). Leica Aperio Imagescope software was used to analyze the images for acute lung injury scoring and the number of neutrophils in the lung tissues.

***Immunofluorescent staining.*** For immunostaining, the sections were deparaffinized and rehydrated sequentially in 100% xylene, 50% xylene:ethanol (EtOH), 100% EtOH, 95% EtOH:H2O, 70% EtOH, 50% EtOH, and 100% ddH_2_O. Tissue slides then underwent antigen retrieval with either 10 mM sodium citrate buffer (pH 6.0) or 10 mM Tris base, 1 mM EDTA buffer (pH 9.0). Tissue slides were then washed, permeabilized in 0.25% Triton-X100 in PBS, and blocked with a 5% BSA solution. Samples were incubated in appropriate primary antibodies for VE-cadherin (1:100, #AF1002, R&D Systems), CD31 (1:50, #ab28364, Abcam), PDPN (1:100, #8.1.1, DSHB), NFATC2 (1:200, #22023-1-AP, Proteintech), SARS-CoV-2 nucleocapsid protein (1:500, #NB100-56576, Novus Biologicals), SPC (1:500, #AB3786, Sigma-Aldrich), KRT8 (1:50, #TROMA-1, DSHB), KI-67 (1:200, #4328926, Invitrogen), ERG (1:200, #ab92513, abcam), ACE2 (1:100, #AF933, R&D Systems), collagen IV (1:40, #AB769, Millipore Sigma), occludin (1:100, #71-1500, Invitrogen), fibronectin (1:200, #A0080, Dako), or DAPI (1:500, #D1306, Invitrogen) overnight at 4°C. After washing, the samples were then incubated with the appropriate and corresponding secondary antibodies for 1-2 hours at room temperature. For tissues with major blood autofluorescence, Vector TrueVIEW Quencher (Vector Laboratories) was used across all slides in an experiment. TUNNEL staining was accomplished after immunofluorescent staining, following the manufacturer’s instructions (abcam; ab66108). Stained tissue sections were imaged using a Zeiss LSM-710 confocal microscope driven by the ZEN software with the following objectives: Plan-Apochromat 63x/1.40 oil, EC Plan-Neofluar 40x/1.30 oil, and Plan-Apochromat 20x/0.8 M27. All image analysis was performed and quantified using ImageJ (Fiji). At least 5 random fields were quantified per lung section. The data obtained from all fields were then averaged to obtain the data for each sample.

***Image analyses.*** For the quantification of the VE-cadherin junction area, projected images were generated by collecting the maximum pixel intensity from each image of the z-stack and projecting it onto a single image. Thresholding was used to remove background noise from images stained with VE-cadherin and CD31. The area of VE-cadherin at the adherens junctions was calculated based on the VE-cadherin positive signal within the overlapping region of the CD31 positive signal, a marker for endothelial cell junctions. The resulting value was then normalized to the area of CD31.

For the quantification of the occludin signal, projected images were generated as described above. Thresholding was used to remove background noise from images. The occludin signal was then normalized to the area of PDPN.

For the quantification of the nuclear NFATC2 signal, projected images were generated as described above. Using DAPI as a guide for the cell nuclei, a mask of each cell nucleus was obtained and applied to the NFATC2 image to remove the signal outside the nuclei. The integrated density of nuclear NFATC2 in each cell was then measured.

For the quantification of the ACE2 signal, projected images were generated as described above. Thresholding was used to remove background noise from images. The ACE2 signal was then quantified.

For the quantification of the endothelial apoptotic cells, ERG, DAPI, and TUNNEL images were composited together. The number of ERG+, DAPI+, and TUNNEL+ cells were then scored per field.

For the quantification of the number of endothelial (ERG) and epithelial (SPC) KI-67+ cells, the specific endothelial or epithelial cell marker was used to composite with KI-67 and DAPI together. The number of SPC+/ERG+, KI-67+, and DAPI+ cells were scored per field.

For the quantification of the number of SPC+KRT8+ transitional cells, the SPC, KRT8, and DAPI images were composited together, and the number of SPC+, KRT8+, and DAPI+ cells were scored per field. The number of transitioning cells was normalized to the area of PDPN.

***Acute lung injury (ALI) scoring.*** The ALI score was calculated according to previously described methods^90^. Briefly, using H&E-stained lung images, the lung was scored for the following: (A) appearance of neutrophils in the alveolar space, (B) appearance of neutrophils in the interstitial space, (C) appearance of hyaline membranes, (D) appearance of proteinaceous debris within the airspace, and (E) appearance of alveolar wall thickening. Each item (A-E) was given a score between 0 and 2, where 0 is the lowest (healthy) score and 2 is the highest (severe) score. The total score for the field was calculated using the following equation: Total ALI Score = [(20A + 14B + 7C + 7D + 2E) / 100], where A-E are the variables described above. A minimum of 15 fields were calculated per mouse, and the total score per animal was averaged. A minimum score of zero represents a healthy lung, and a maximum score of 1 represents a lung with severe injury.

***Differential cell analysis in BAL.*** A small incision was made across the width of the trachea for cannulation with a 20G blunt-tip needle attached to a syringe. The lungs were then instilled with sterile, pyrogen-free physiological saline until 3 mL of lavage was obtained. The lavage was then centrifuged at 500 g for 10 minutes, and the resulting supernatant was used for cytokine analysis. The cell pellet was resuspended in 1 mL of 1% BSA in sterile saline. The cell pellet was placed on a slide, entrained, and spread over with an even stroke of a second slide, repeated in triplicate per BAL sample. The slides were then dried before staining with Diff-Quik (AMR Vet Collective) according to the manufacturer’s instructions. The slides were covered with coverslips before imaging on an Olympus BX51 brightfield microscope. At least 100 cells were counted and characterized per sample.

***Pulmonary endothelial cell isolation.*** Lung tissues were obtained on days 3 and 5 following LPS challenge, as well as from healthy naïve mice. Each tissue was cut into very small pieces, and 1 mg/mL collagenase (Roche Applied Science) in HBSS was added to the tissue and set to incubate for 1 hour in a shaking water bath set to 200 rpm at 37°C. After the initial digestion, the tissue was passed through a 100-µm cell strainer to create a single-cell suspension. CD45+ cells were first depleted using Dynabeads CD45 antibodies (#11153D, Thermo Fisher). Pulmonary endothelial cells were then isolated using Dynabeads CD31 (#11155D, Thermo Fisher) and washed thoroughly to remove beads.

***RNA isolation.*** Total RNA for bulk RNA sequencing from the isolated pulmonary endothelial cells was obtained using TRIzol (#15596026, Invitrogen) according to the manufacturer’s instructions. The concentration of the isolated RNA was measured using a Nanodrop 1000 (Thermo Fisher) at 260 nm, and quality was assessed by the 260/280 optical density ratio. The libraries were obtained through reverse transcription of the RNA samples to cDNA using a high-capacity cDNA reverse transcription kit (#4368814, Thermo Fisher). The resulting cDNA libraries were sent to the University of Chicago’s Genomics Facility for bulk RNA sequencing.

***RNA-sequencing.*** Bulk RNA sequencing was performed by the University of Chicago’s Genomics Facility. The RNA quality and quantity were assessed using Agilent’s bioanalyzer, where all samples showed an RNA integrity number greater than eight. Strand-specific RNA-Seq libraries were prepared using the TruSeq mRNA RNA-Seq library protocol (Illumina). Library quality and quantity were assessed using Agilent’s bioanalyzer. All libraries were sequenced using an Illumina NovaSeq 6000 (Illumina).

***RNA-sequencing analysis.*** Raw reads were aligned to the mouse reference genome mm10 using STAR^91^. ENSEMBL gene expression was quantified using FeatureCounts^92^. Differential expression statistics (fold-change and p-value) were computed using edgeR^93^, and p-values were adjusted for multiple testing using the false discovery rate (FDR) correction of Benjamini and Hochberg^94^.

Clustering of differentially expressed genes (FDR < 0.05) was performed using k-means clustering with reproducibility statistics to determine the optimal number of clusters. We performed k-means clustering with ten random initializations on a range of cluster numbers (k = 2 to 20). For each k, we evaluated the reproducibility of the repeated clustering runs by computing the pairwise distance between clustering results. Specifically, we calculated the fraction of co-clustered feature pairs across two clustering results relative to the number of co-clustered feature pairs within each result individually. This difference was averaged across all result pairs for each value of k, and the largest k with an average distance less than 1e-5 was selected as the k with highly reproducible clusters. This procedure yielded 8 clusters for our data. Patterns from the clusters will be visualized using heatmaps and boxplots to aid in interpretation.

Sequencing pathway enrichment was performed using QIAGEN Ingenuity Pathway Analysis (IPA) on differentially expressed genes to obtain canonical pathways within each cluster. The analysis utilized the calculated Z-score, p-value, and q-value generated from edgeR in R programming to perform predictive and literature-based predictions of regulations.

To visualize Foxm1 downstream signaling, Foxm1 target genes were downloaded from two sources: the “FOXM1 transcription factor network” from the NCI Pathway Interaction Database^95^ and FOXM1 target genes from MSigDB (FOXM1_01, <https://www.gsea-msigdb.org/gsea/msigdb/human/geneset/FOXM1_01.html>)^96,97^. These target genes were cross-referenced with our differentially expressed genes and gene clusters.

***Public data analysis.*** Single-cell RNA sequencing (scRNA-seq) data and associated metadata were obtained from the National Center for Biotechnology Information’s Gene Expression Omnibus (GEO) under accession number GSE171524^59^. The pulmonary endothelial cell cluster was isolated and used for downstream analysis. Differentially expressed genes (DEGs) were identified based on a q-value threshold of 0.20. Fold changes were calculated by comparing gene expression in COVID-19 patients versus healthy control individuals. Pathway enrichment analysis was performed using QIAGEN’s Ingenuity Pathway Analysis (IPA) on DEGs to obtain canonical pathways within the endothelial cluster. Statistical analysis was calculated using the EdgeR package in R, and both Z score and q-value were used for literature-based inference of regulatory networks and pathways.

**
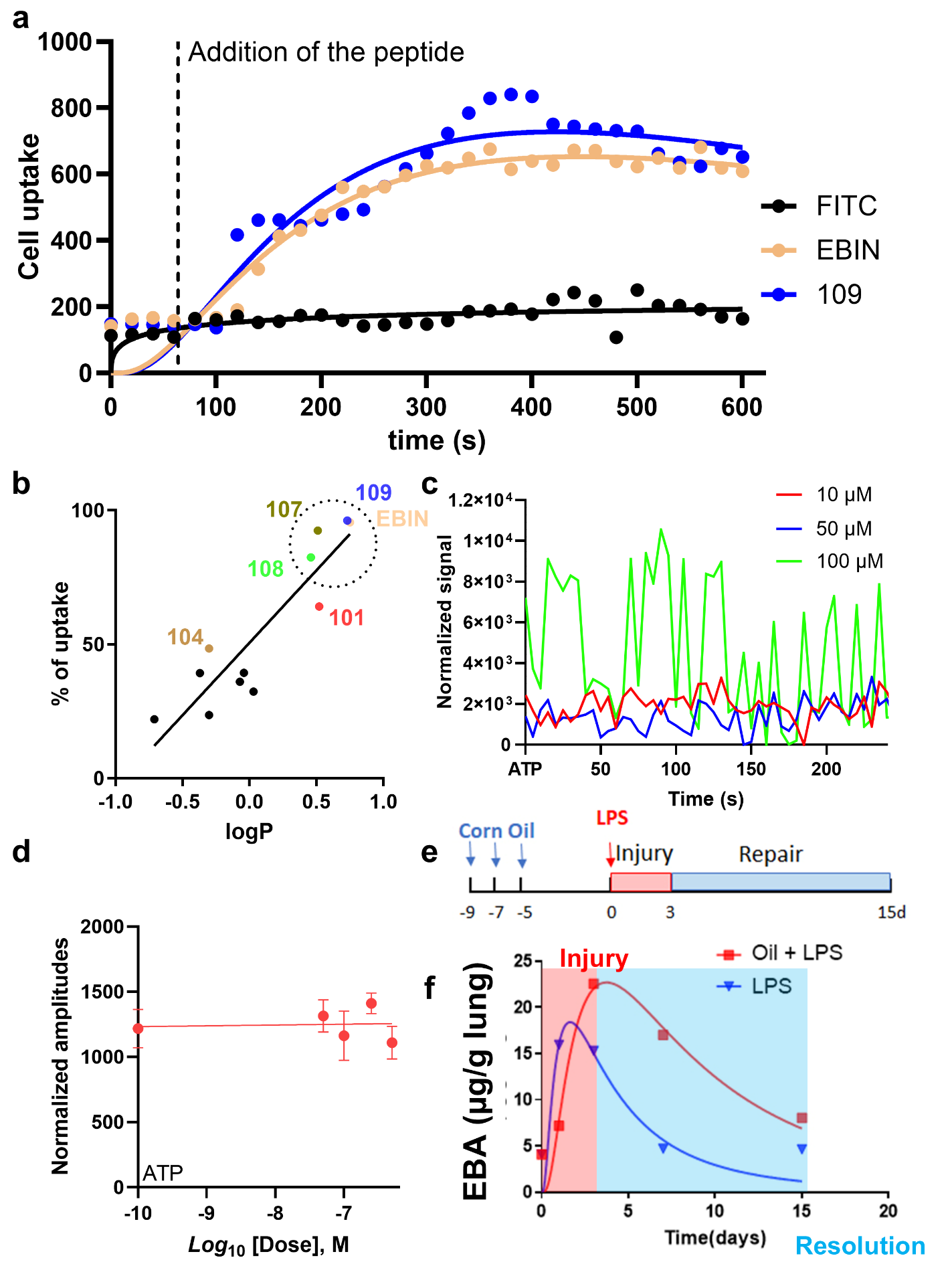
**

**Supplementary Figure 1. Physicochemical and biochemical properties of designed inhibitors.**

**a** Representative traces of EB3 inhibitor uptake by endothelial cells. The 5’6-FAM-labeled peptides were added to the cell culture medium at 50 s (dashed line), and intracellular fluorescence was used as a measure of peptide uptake. A lognormal function was used to fit the curve for each peptide.

**b** The percentage of selected peptide uptake by endothelial cell as a function of lipophilicity (log scale). (n = 3 independent experiments).

**c** Dose-dependent calcium response of isolated primary murine AT2 cells to ATP at10 (red), 50 (blue), and 100 µM (green).

**d** Concentration-dependent curves showing changes in intracellular calcium levels in primary AT2 cells following stimulation with 100 µM ATP *versus* varying concentrations of VT-109 (log scale).

**e-f** A schematic representation of the treatment schedule with oil and LPS (e) and the time course of EBA extravasation (f) in the lungs of mice challenged i.p. with 4 mg/kg bw LPS, with (red) and without (blue) oil pre-loading. Corn oil was administered i.p. three times at 12ml/kg body weight every 48 hours. LPS was administrated on day 5 following the last injection of corn oil. mean$\pm$S.D; n=3 mice per group. A smooth line function was used to plot the curve.

Supplemental data to Figures 2 and 3.


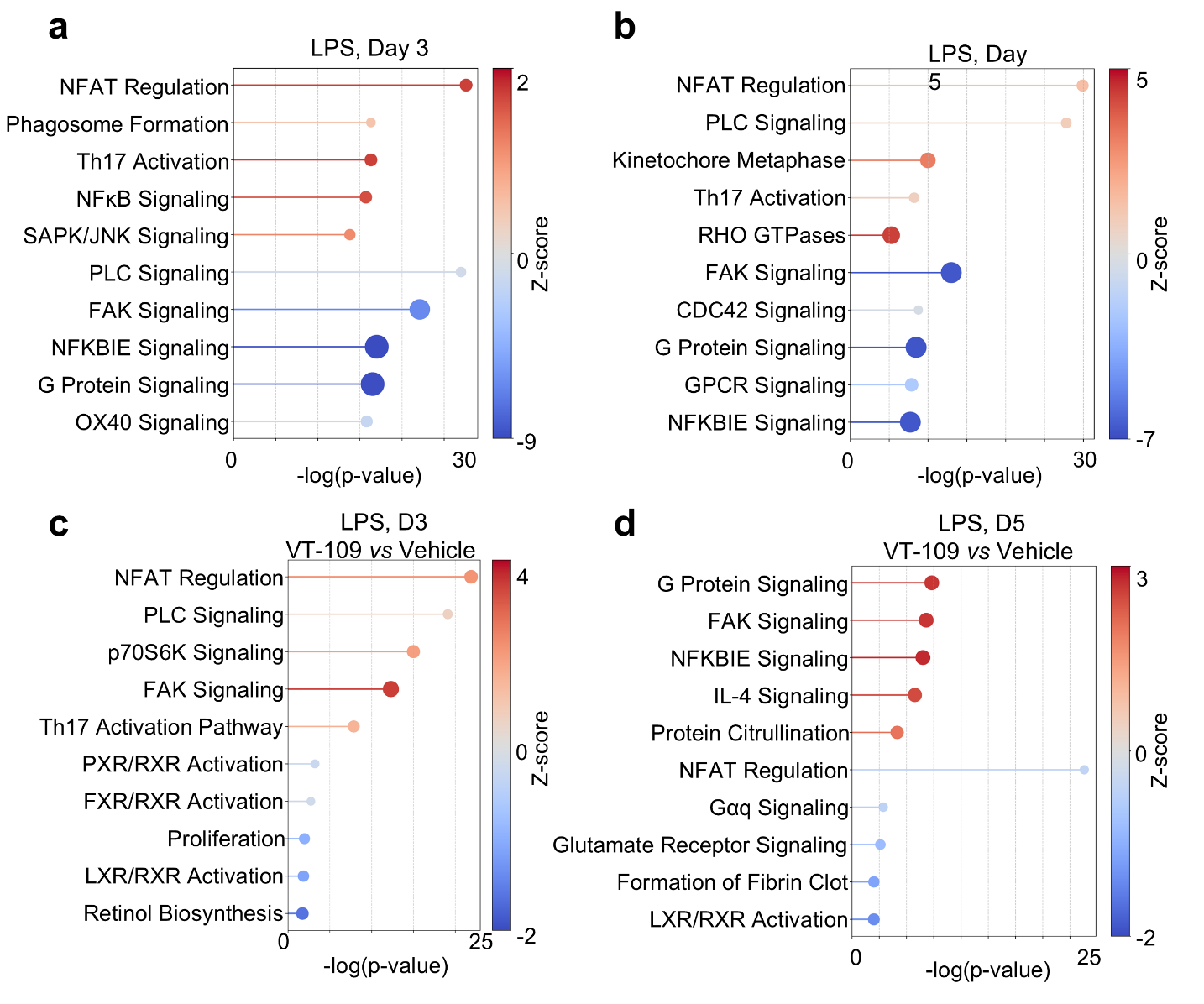


**Supplementary Figure 2. Pathway analysis of transcriptomic changes in endothelial cells from mice challenged with endotoxin and treated with either vehicle or VT-109 during the onset and late phases of injury.**

**a-d** Lollipop graphs showing the top relevant pathways affected by LPS on days 3 (a) and 5 (b) after LPS challenge (LPS *vs* healthy naïve mice); altered by VT-109 treatment on days 3 (c) and 5 (d) after LPS challenge (VT-109 *vs* vehicle). (n = 3 mice per group).

Supplemental data to Figure 3.

**
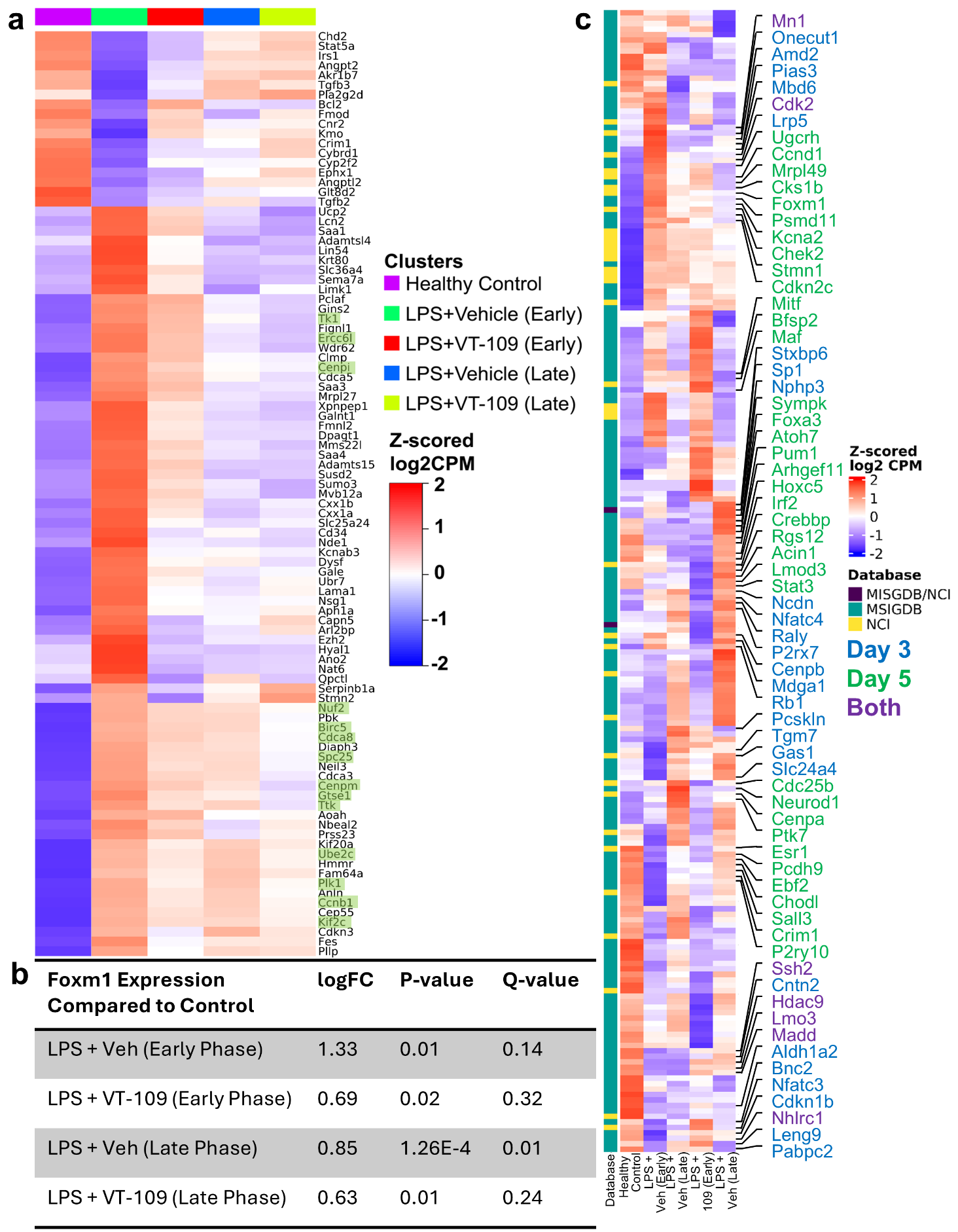
**

**Supplementary Figure 3. VT-109 reversed the expression of pro-inflammatory genes and promoted the FOXM1-mediated reparative program in pulmonary endothelial cells of LPS-challenged mice.**

**a** Heatmap showing selected genes that VT-109 restored to the baseline levels (healthy control) in endothelial cells of mice challenged with endotoxin. Experimental groups are labeled in each column, and each row represents a gene. The color scale shows gene expression changes, with upregulation in red and downregulation in blue, presented as Z scored log2 counts per million reads mapped (CPM). Genes associated with the cell cycle are highlighted in green. (n = 3 mice per group).

**b** Changes in *Foxm1* gene expression in the indicated groups relative to healthy control.

**c** Heatmap of target genes predicted to be regulated by FOXM1 transcription activity. Experimental groups are labeled accordingly. Each column represents a gene. VT-109-mediated normalization of the genes within the FOXM1 pathway is highlighted for the early phase (the onset; blue), late phase (green), and both (purple).

Supplemental data to Figure 3.


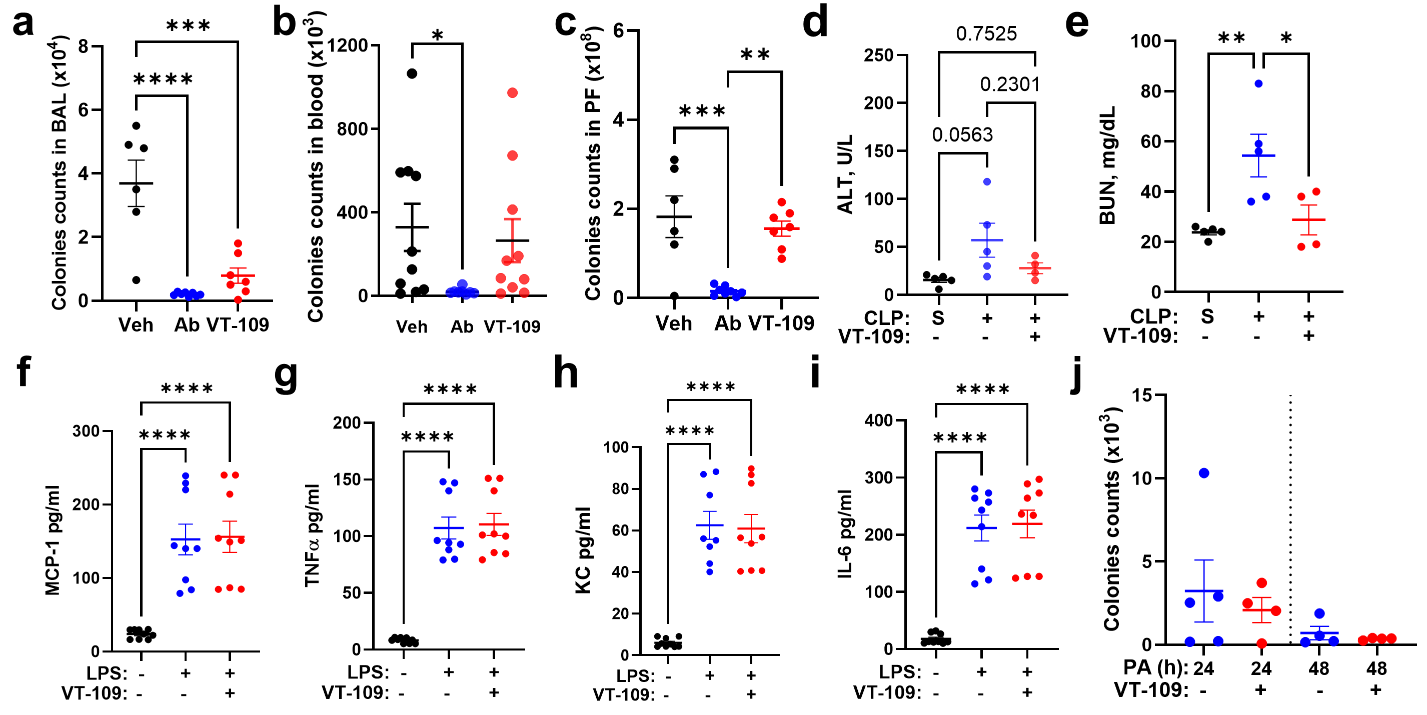


**Supplementary Figure 4. VT-109 treatment protects against multi-organ failure but does not alter cytokine levels secreted by immune cells.**

**a-c** The bacterial colony counts in bronchoalveolar lavage (BAL; a), blood (b), and peritoneal fluid (PF; c) of mice 24 hours after CLP surgery. Mice received indicated treatments as in Fig. 4a. (n = 6-10 mice per group) *, *P*<0.05 using Kruskal-Wallis with Dunn’s multiple comparison test in (b).

**d-e** Blood levels of organ damage markers, ALT (d) and BUN (e), measured 24 hours after sham or CLP surgery in mice. Mice were treated with an i.v. vehicle control (blue) or 2 µmol/kg bw VT-109 (red) administered 5 hours post-surgery. (n = 5 mice per group).

**f-i** *In vitro* levels of inflammatory cytokines, MCP-1 (f), TNFα (g), KC (h), and IL-6 (i), secreted by naïve (baseline) or LPS-challenged hematopoietic stem cells, with or without VT-109 treatment as described.

**j** Bacteria colony counts in the lung of mice at 24 and 48 hours after i.t. infection with *P. aeruginosa* with and without VT-109 as described (n = 4-5 mice per group).

Data as mean±SEM. *, *P*<0.05; **, *P*<0.01; ***, *P<*0.001, and ****, *P*<0.0001 using one-way ANOVA with Tukey’s post hoc test. Supplemental data to Figure 4-5.


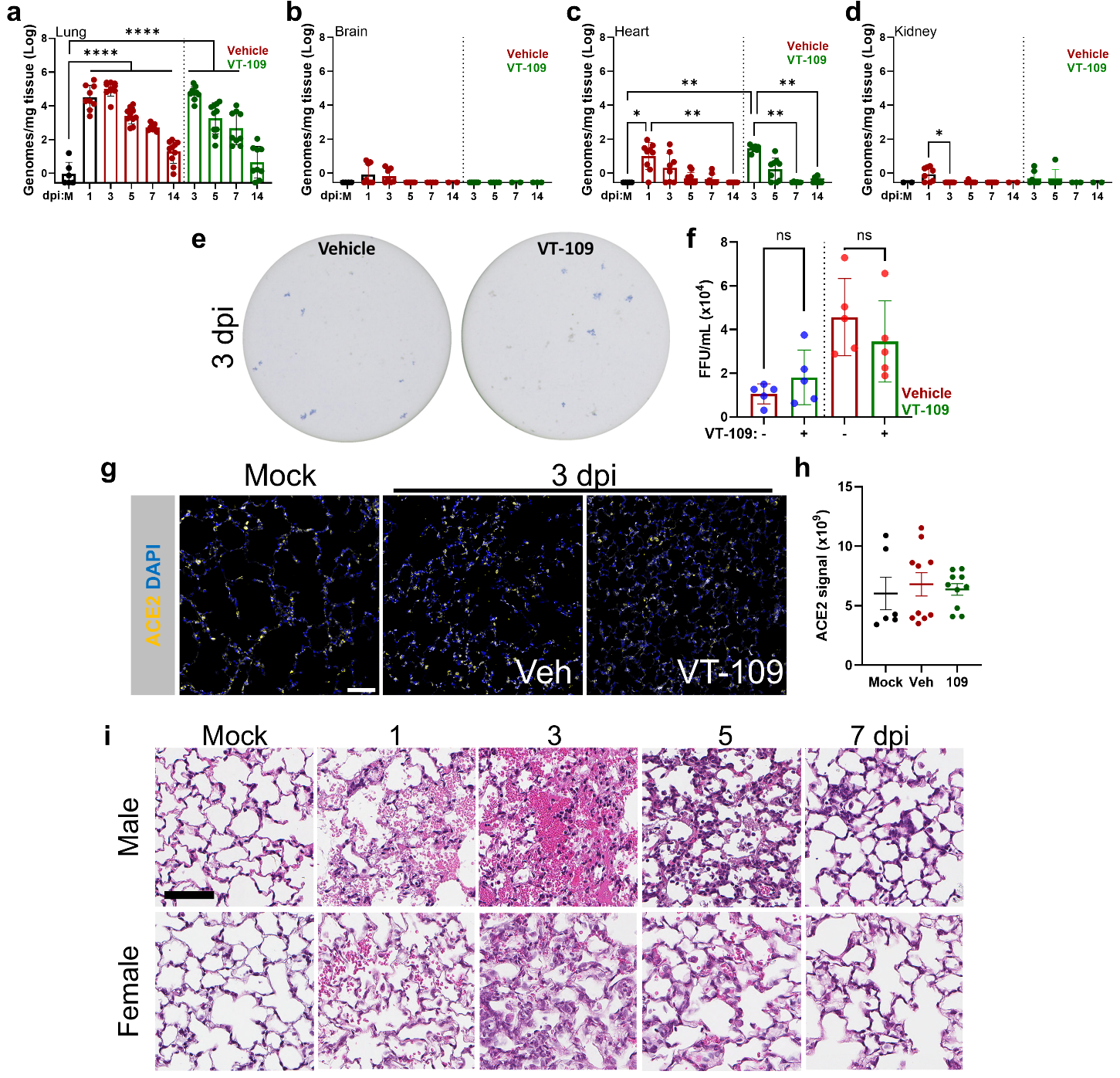


**Supplementary Figure 5. VT-109 treatment does not affect SARS-CoV-2 replication.**

**a-d** Time-course of SARS-CoV-2-MA10 genome in the lungs (a), brain (b), heart (c), and kidney (d) tissues of BALB/c mice infected with i.n. 1x10^4^ PFU of the SARS-CoV-2 MA10 virus, treated with either i.v. vehicle (red) or 250 nmol/kg b.w. VT-109 (green). (n = 8-10 mice per group) *, *P*<0.05; **, *P*<0.01; and ****, *P*<0.0001 using two-way ANOVA with Sidak’s multiple comparison test.

**e-f** Representative images (e) and quantifications (f) of SARS-CoV-2 MA10 colonies in a focus-forming assay from lung tissue of male (blue) and female (red) mice at 3 dpi with treatment as described. (n = 5 mice per sex per group) ns, not significant using two-tailed t-test.

**g-h** Representative images of lungs stained for ACE2 (yellow) and DAPI (blue) (g) and quantifications of ACE2 signals (h) at 3 dpi; treatment groups as indicated. Scale bar, 50µm. No statistical significance using one-way ANOVA with Tukey’s post hoc test. (n = 6-10 mice per group).

**i** Representative H&E-stained images of mouse lungs infected with the SARS-CoV-2 MA10 virus as in (a).

Data as mean±SEM. Supplemental data to Figure 6.

**
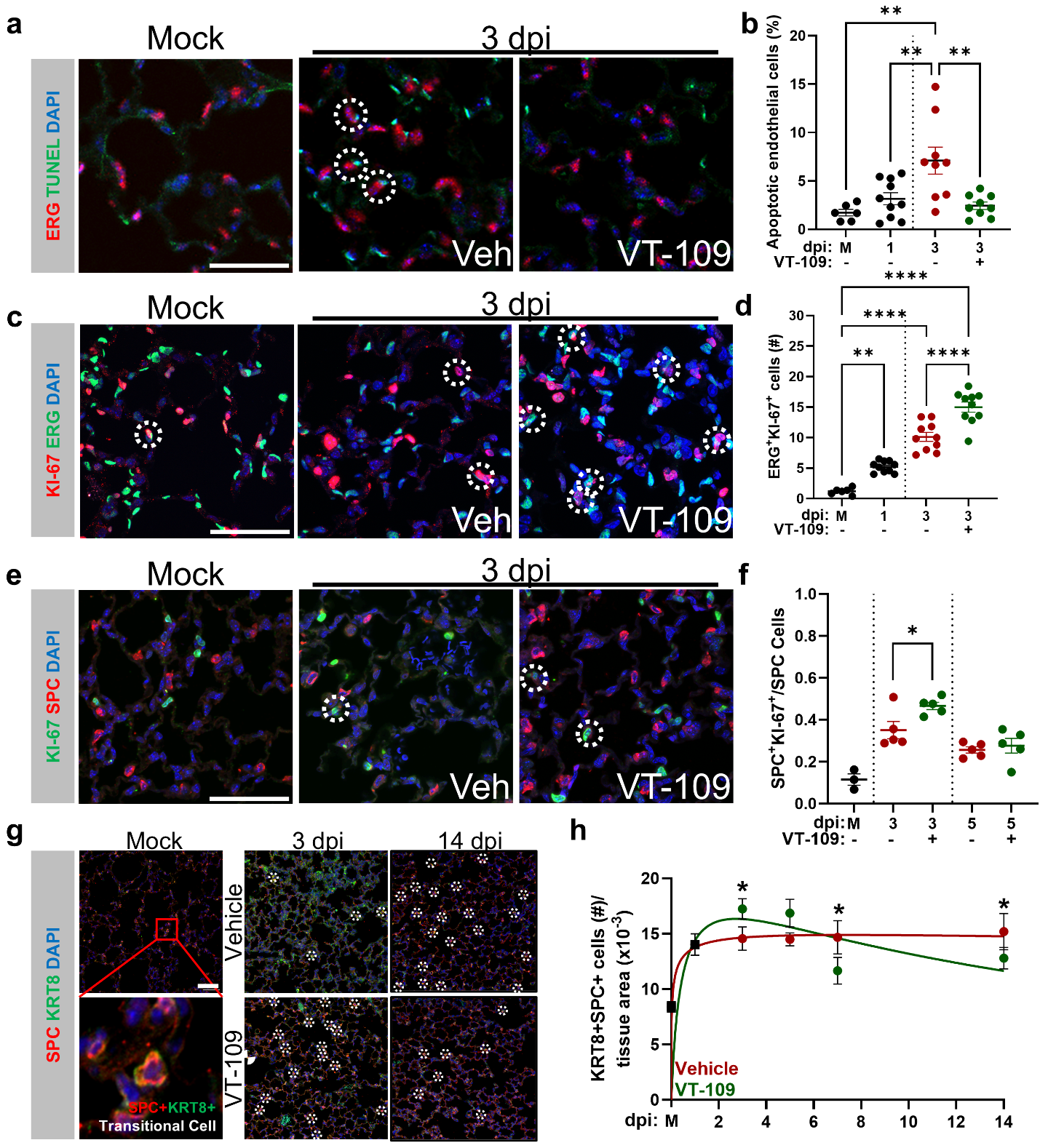
**

**Supplementary Figure 6. VT-109 treatment prevents endothelial cell death and promotes lung regeneration.**

**a-b** Representative images of lung tissue stained for ERG (red), TUNEL (green), and DAPI (blue) (a) and quantification of apoptotic endothelial cells expressed as the ratio of TUNEL⁺/ ERG⁺/DAPI⁺ cells to ERG⁺/DAPI⁺ cells (b). Scale bar, 50 µm. Dashed circles highlight ERG- and TUNEL-positive cells in each image. (n = 6-10 mice per group).

**c-d** Representative images of lung tissue stained for KI-67 (red), ERG (green), and DAPI (blue) (c), and quantification of KI-67⁺/ERG⁺ double-positive cells (d). Scale bar, 50 µm. Dashed circles highlight KI-67⁺/ERG⁺ cells in each image. (n = 6-10 mice per group).

**e-f** Representative images of lungs stained for KI-67 (green), SPC (red), and DAPI (blue) (e), and quantification of KI-67⁺/SPC⁺ double-positive cells (f). Scale bar, 50 µm. Dashed circles highlight KI-67⁺/SPC⁺ double-positive cells in each image. (n = 3-6 mice per group). *, *P*<0.05 using two-tailed *t*-test comparing vehicle- and VT-109-treated mice at the respective time point.

**g-h** Representative images of lungs stained for SPC (red), KRT8 (green), and DAPI (blue) at indicated times of post-infection (g) and quantification of the number of KRT8⁺/SPC⁺ double-positive cells (h). Enlarged insert shows the colocalization of SPC and KRT8. Dashed circles highlight SPC- and KRT8-positive cells in each image. Scale bar**,** 50 µm. Insert scale bar, 10 µm. (n = 6 mice per group) *, p<0.05 using two-tailed *t*-test comparing vehicle- and VT-109-treated mice at the respective time point.

Data as mean±SEM. *, *P*<0.05; **, *P<0.01;* and ****, *P*<0.0001 using one-way ANOVA with Tukey’s post hoc test unless otherwise indicated. Supplemental data to Figure 6.


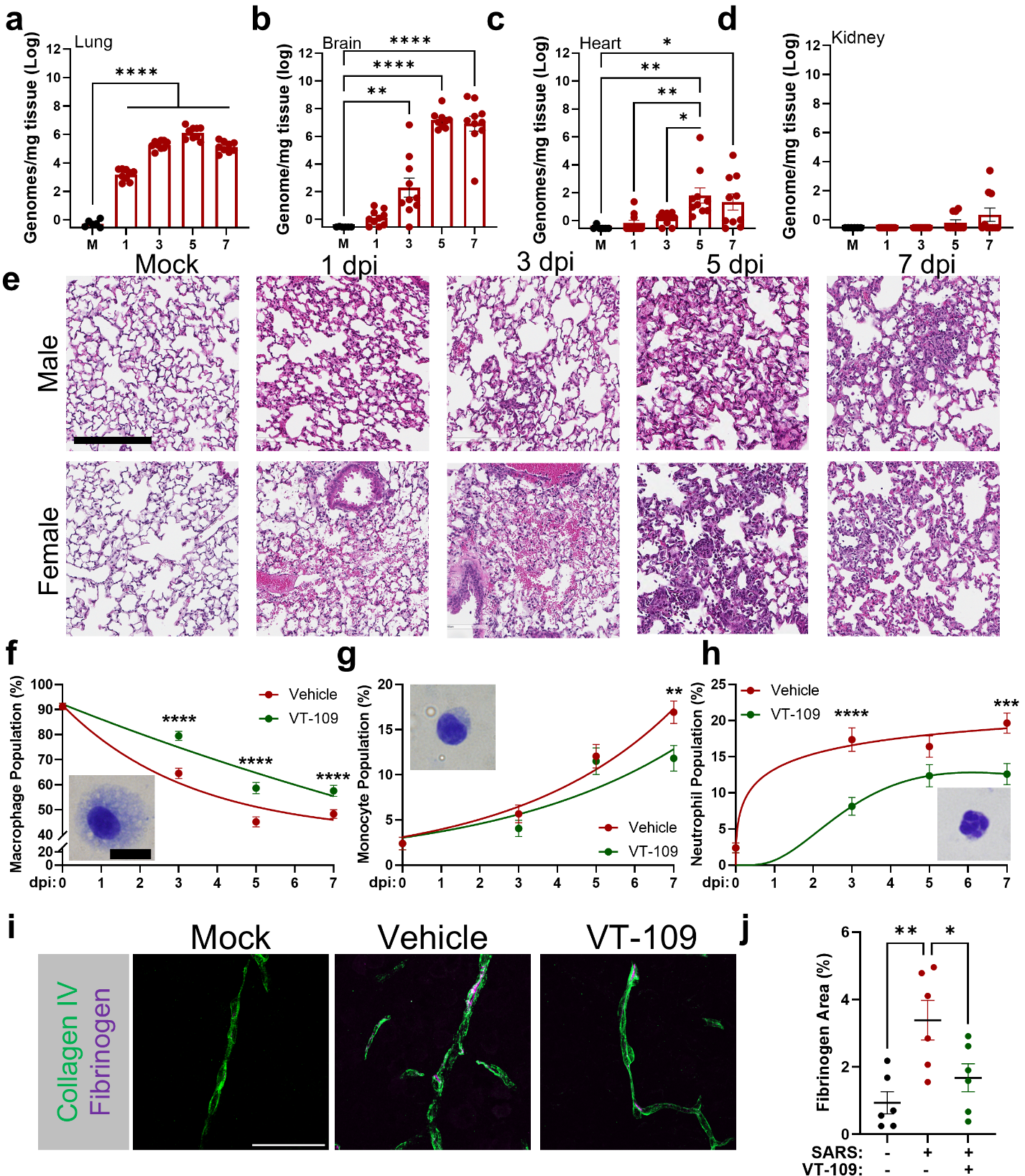


**Supplementary Figure 7. VT-109 treatment restores immune homeostasis in K18-hACE2 mice infected with the Washington strain** **SARS-CoV-2.**

**a-d** SARS-CoV-2 genome detected in the lung (a), brain (b), heart (c), and kidney (d) tissues of K18-hACE2 mice infected with 1x10^4^ PFU of the Washington strain of SARS-CoV-2. (n = 8-10 mice per group). *, *P*<0.05; **, *P*<0.01; and ****, *P*<0.0001 using one-way ANOVA with Tukey’s post hoc test.

**e** Representative H&E-stained images of lungs from male and female K18-hACE2 mice infected with SARS-CoV-2 as in (a).

**f-h** Differential counts and representative Diff-Quik-stained images of immune cells, macrophages (f); monocytes (g); and neutrophils (h); detected in the BAL of mice infected as in (a) and treated i.v. with either vehicle (red) or 2 µmol/kg bw VT-109 (green), starting on 1 dpi. (n = 6 mice per group). **, *P*<0.01; ***, P<0.001; and ****, *P*<0.0001 using two-tailed unpaired t-test between vehicle or VT-109-treated mice at the respective time point.

**i-j** Representative images of the cerebral cortex stained for fibrinogen (magenta) and collagen IV (green) (i), and quantification of the fibrinogen-positive area within the vessels (j) at 5 dpi. Scale bar, 50 µm. (n = 6 mice per group). *, *P*<0.05 and **, *P*<0.01 using one-way ANOVA with Tukey’s post hoc test.

Data as mean±SEM. Supplemental data to Figure 7.

| ID | Type of Peptide | Chemical Formula | IUPAC Name | Structure |
| --- | --- | --- | --- | --- |
| 000 | Linear | C_39_H_61_N_7_O_12_ | (*S*)-4-((2*S*,3*R*)-2-((*S*)-2-amino-3-phenylpropanamido)-3-hydroxybutanamido)-5-(((2*S*,3*S*)-1-((S)-2-(((2*S*,3*R*)-1-(((1*S*,2*S*)-1-carboxy-2-methylbutyl)amino)-3-hydroxy-1-oxobutan-2-yl)carbamoyl)pyrrolidin-1-yl)-3-methyl-1-oxopentan-2-yl)amino)-5-oxopentanoic acid | 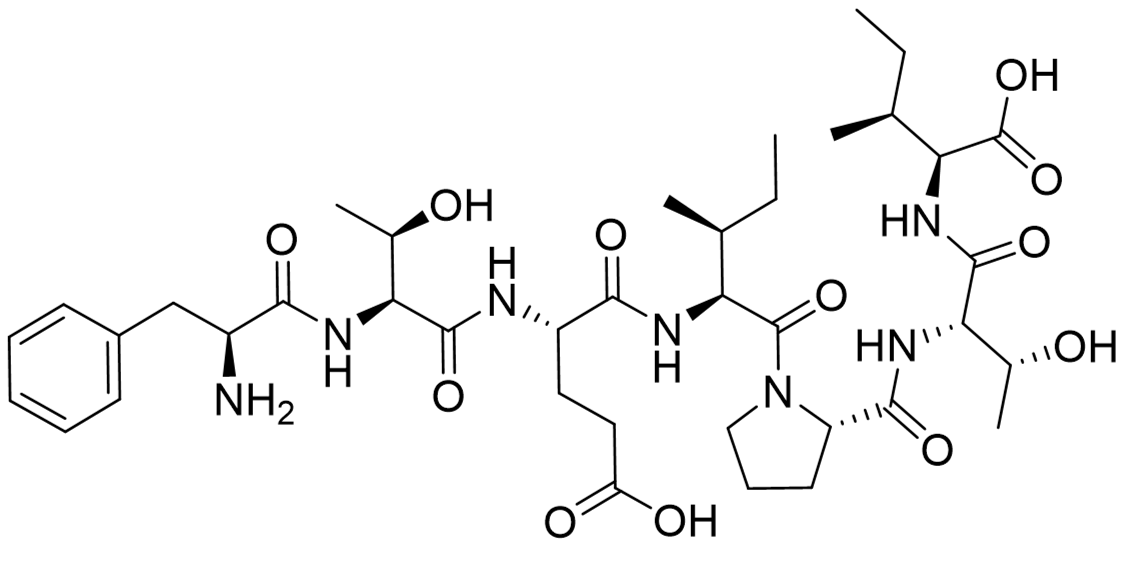 |
| 004 | Linear | C_39_H_61_N_7_O_12_ | (*S*)-4-((2*S*,3*R*)-2-((*R)*-2-amino-3-phenylpropanamido)-3-hydroxybutanamido)-5-(((2S,3S)-1-((S)-2-(((2*S*,3*R*)-1-(((1*S*,2*S*)-1-carboxy-2-methylbutyl)amino)-3-hydroxy-1-oxobutan-2-yl)carbamoyl)pyrrolidin-1-yl)-3-methyl-1-oxopentan-2-yl)amino)-5-oxopentanoic acid | 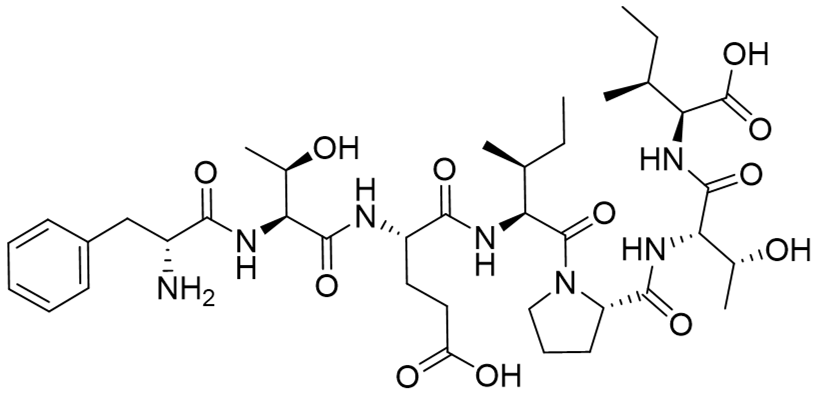 |
| 016 | Linear with Small Protein-like Chains | C_41_H_65_N_7_O_12_ | (*R*)-4-((*S*)-2-((*S*)-2-amino-3-phenylpropanamido)-3-hydroxy-3-methylbutanamido)-5-(((*S*)-1-((*S*)-2-(((2*S*,3*R*)-1-(((1*S*,2*S*)-1-carboxy-2-methylbutyl)amino)-3-hydroxy-1-oxobutan-2-yl)carbamoyl)pyrrolidin-1-yl)-4,4-dimethyl-1-oxopentan-2-yl)amino)-5-oxopentanoic acid | 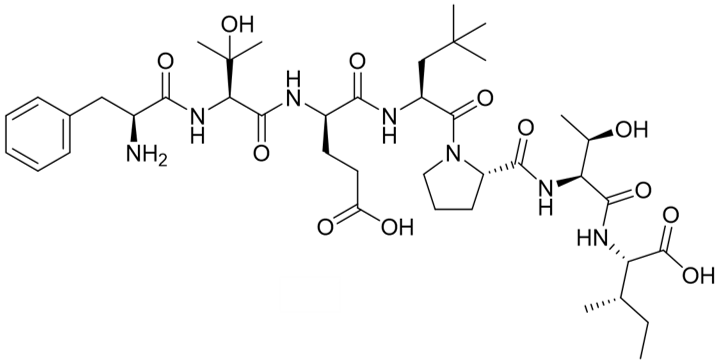 |
| 019 | Linear with Small Protein-like Chains | C_39_H_59_N_7_O_12_ | (*S)*-4-((2*S*,3*R*)-2-((*S*)-2-amino-3-phenylpropanamido)-3-hydroxybutanamido)-5-(((*S*)-1-((*S*)-2-(((2*S*,3*R*)-1-(((1*S*,2*S*)-1-carboxy-2-methylbutyl)amino)-3-hydroxy-1-oxobutan-2-yl)carbamoyl)pyrrolidin-1-yl)-4-methyl-1-oxopent-4-en-2-yl)amino)-5-oxopentanoic acid | 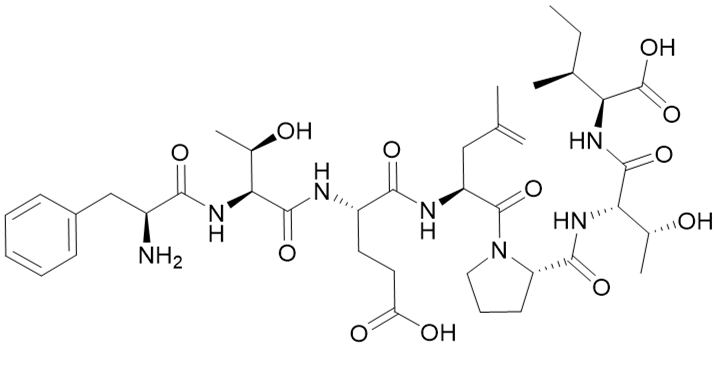 |
| 021 | Linear with Small Protein-like Chains | C_43_H_63_N_7_O_10_ | (4-(((*R*)-2-((*S*)-2-amino-3-phenylpropanamido)-3-hydroxy-3-methylbutanamido)methyl)benzoyl)-L-isoleucyl-L-prolyl-L-threonyl-L-isoleucine | 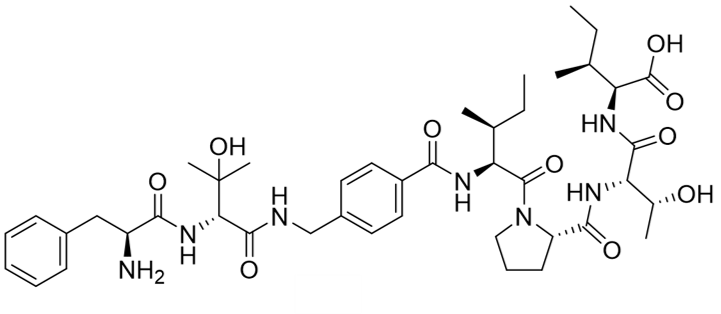 |
| 036 | Linear with Small Protein-like Chains | C_40_H_61_N_7_O_12_ | (*S*)-4-((*R*)-2-((*R*)-2-amino-3-phenylpropanamido)-3-hydroxy-3-methylbutanamido)-5-(((*S*)-1-((*S*)-2-(((2*S*,3*R*)-1-(((1*S*,2*S*)-1-carboxy-2-methylbutyl)amino)-3-hydroxy-1-oxobutan-2-yl)carbamoyl)pyrrolidin-1-yl)-4-methyl-1-oxopent-4-en-2-yl)amino)-5-oxopentanoic acid | 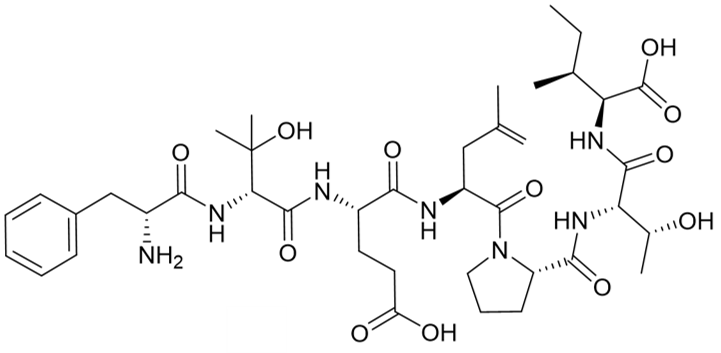 |
| 038 | Linear with Small Protein-like Chains | C_41_H_65_N_7_O_13_ | (*S*)-4-((*R*)-2-((*S*)-2-amino-3-phenylpropanamido)-3-hydroxy-3-methylbutanamido)-5-(((*S*)-1-((2*S*,4*S*)-2-(((2*S*,3*R*)-1-(((1*S*,2*S*)-1-carboxy-2-methylbutyl)amino)-3-hydroxy-1-oxobutan-2-yl)carbamoyl)-4-hydroxypyrrolidin-1-yl)-4,4-dimethyl-1-oxopentan-2-yl)amino)-5-oxopentanoic acid | 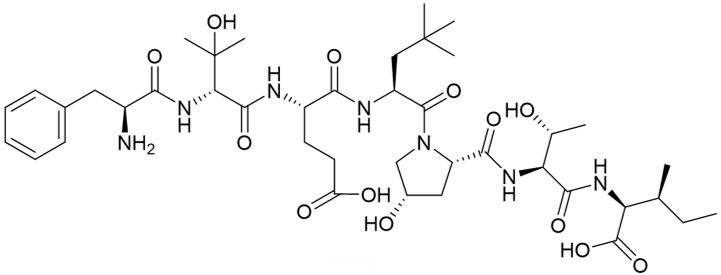 |
| 040 | Linear with Small Protein-like Chains | C_46_H_73_N_7_O_12_ | (*S*)-4-((*R*)-2-((*S*)-2-amino-3-phenylpropanamido)-3-hydroxy-3-methylbutanamido)-5-(((*S*)-1-((1S,3a*S*,7a*R*)-1-(((R)-1-(((1*S*,2*S*)-1-carboxy-2-methylbutyl)amino)-3-hydroxy-3-methyl-1-oxobutan-2-yl)carbamoyl)octahydro-2H-isoindol-2-yl)-4,4-dimethyl-1-oxopentan-2-yl)amino)-5-oxopentanoic acid. | 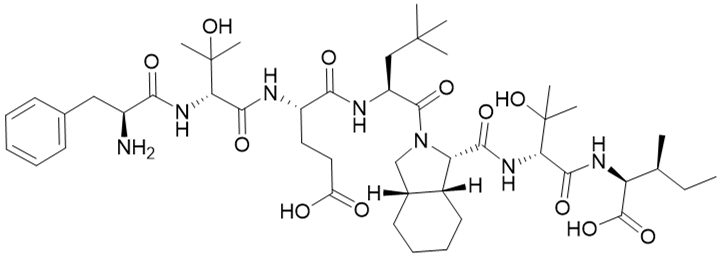 |
| 041 | Linear with Small Protein-like Chains | C_48_H_71_N_7_O_10_ | (4-((1*S*,3a*S*,7a*R*)-2-((*S*)-2-(3-(((*R*)-2-((*S*)-2-amino-3-phenylpropanamido)-3-hydroxy-3-methylbutanamido)methyl)benzamido)-4,4-dimethylpentanoyl)octahydro-1H-isoindole-1-carboxamido)-3-hydroxybutanoyl)-L-isoleucine | 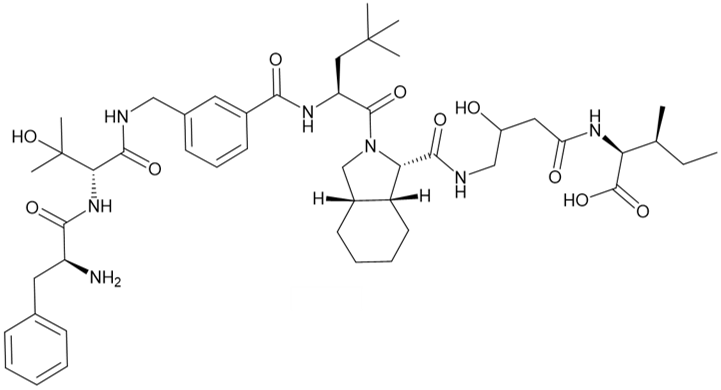 |
| 042 | Linear with Small Protein-like Chains | C_45_H_67_N_7_O_11_ | ((*R*)-2-((2*S*,4*S*)-1-((*S*)-2-(3-(((*R*)-2-((*S*)-2-amino-3-phenylpropanamido)-3-hydroxy-3-methylbutanamido)methyl)benzamido)-4,4-dimethylpentanoyl)-4-hydroxypyrrolidine-2-carboxamido)-3-hydroxy-3-methylbutanoyl)-L-isoleucine | 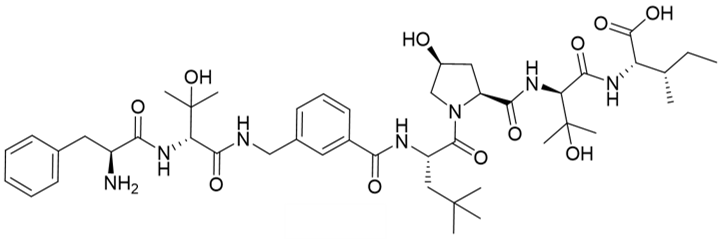 |
| 044 | Linear with Small Protein-like Chains | C_41_H_64_N_8_O_11_ | (*S*)-4-((*R*)-2-((*R*)-2-amino-3-phenylpropanamido)-3-hydroxy-3-methylbutanamido)-5-(((*S*)-1-((*S*)-2-(((2*S*,3*R*)-1-(((*S*)-1-amino-4-methyl-1-oxopent-4-en-2-yl)amino)-3-hydroxy-1-oxobutan-2-yl)carbamoyl)pyrrolidin-1-yl)-4,4-dimethyl-1-oxopentan-2-yl)amino)-5-oxopentanoic acid | 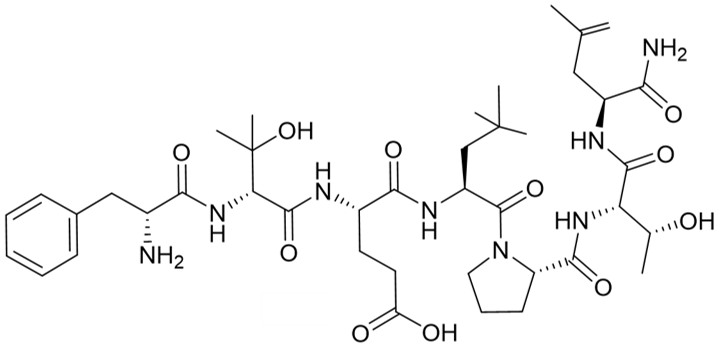 |
| 049 | Linear with Small Protein-like Chains | C_40_H_59_N_7_O_12_ | (*S*)-2-((2*S*,3*R*)-2-((*S*)-1-((*R*)-2-((*S*)-2-((*R*)-2-((*R*)-2-amino-3-phenylpropanamido)-3-hydroxy-3-methylbutanamido)-4-carboxybutanamido)-4-methylpent-4-enoyl)pyrrolidine-2-carboxamido)-3-hydroxybutanamido)-4-methylpent-4-enoic acid. | 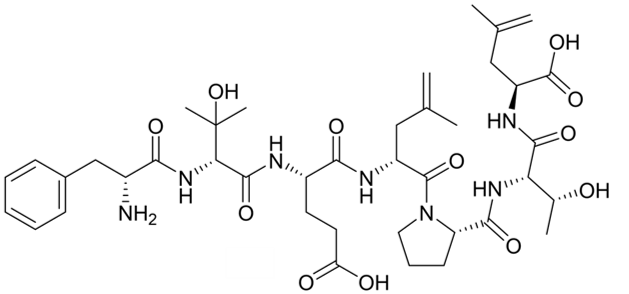 |
| 101 | Linear with Small Protein-like Chains | C_49_H_73_N_7_O_10_ | (2*S*)-2-(4-((2*S*,3a*S*,7a*S*)-1-((*S*)-2-(4-(((*R*)-2-((*R*)-2-amino-3-phenylpropanamido)-3-hydroxy-3-methylbutanamido)methyl)benzamido)-4,4-dimethylpentanoyl)octahydro-1H-indole-2-carboxamido)-3-hydroxybutanamido)-4,4-dimethylpentanoic acid. | 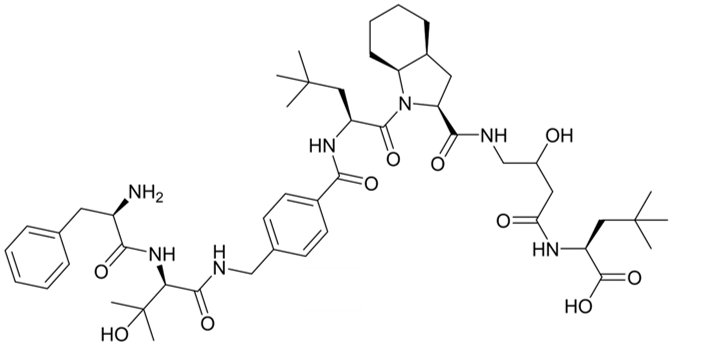 |
| 102 | Linear with Small Protein-like Chains | C_47_H_67_N_7_O_10_ | (2*S*)-2-(4-((2*S*,3a*S*,7a*S*)-1-((2*S*)-2-(4-((4-((*R*)-2-amino-3-phenylpropanamido)-3-hydroxybutanamido)methyl)benzamido)-4-methylpent-4-enoyl)octahydro-1H-indole-2-carboxamido)-3-hydroxybutanamido)-4,4-dimethylpentanoic acid. | 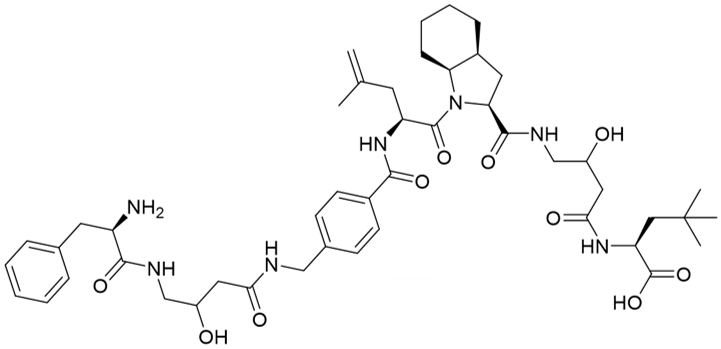 |
| 103 | Linear with Small Protein-like Chains | C_40_H_64_N_6_O_9_ | (2*S*)-2-(4-((2*S*,3a*S*,7a*S*)-1-((S)-2-(4-(((R)-2-amino-3-hydroxy-3-methylbutanamido)methyl)benzamido)-4,4-dimethylpentanoyl)octahydro-1H-indole-2-carboxamido)-3-hydroxybutanamido)-4,4-dimethylpentanoic acid | 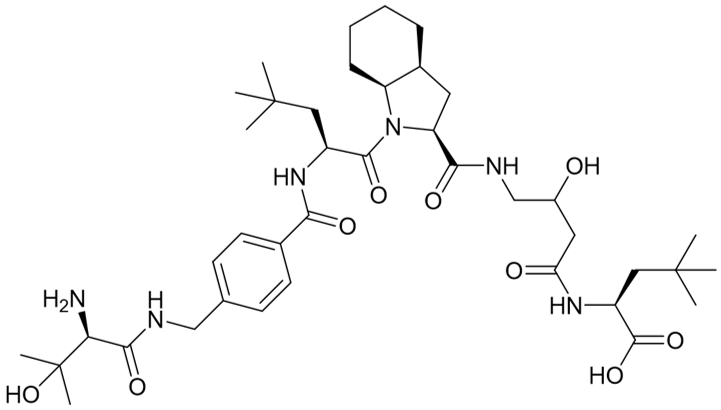 |
| 104 | Linear with Small Protein-like Chains | C_38_H_58_N_6_O_9_ | (2*S*)-2-(4-((2*S*,3a*S*,7a*S*)-1-((2*S*)-2-(4-((4-amino-3-hydroxybutanamido)methyl)benzamido)-4-methylpent-4-enoyl)octahydro-1H-indole-2-carboxamido)-3-hydroxybutanamido)-4,4-dimethylpentanoic acid. | 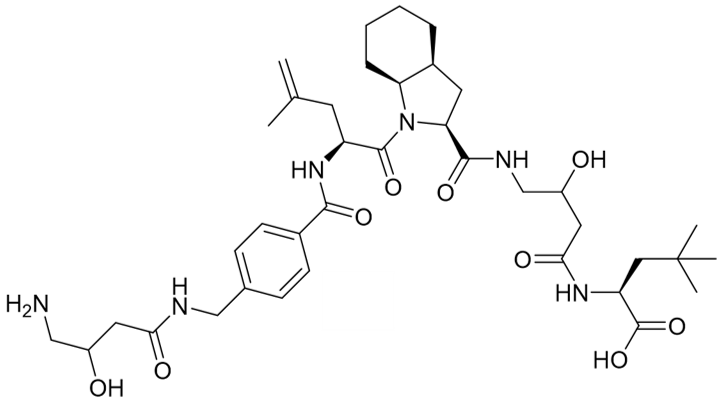 |
| 105 | Linear with Small Protein-like Chains | C_47_H_65_N_7_O_10_ | (2*S*)-2-(4-((2*S*,3a*S*,7a*S*)-1-((*S*)-2-(4-(((*R*)-2-((*R*)-2-amino-3-phenylpropanamido)-3-hydroxy-3-methylbutanamido)methyl)benzamido)-4-methylpent-4-enoyl)octahydro-1H-indole-2-carboxamido)-3-hydroxybutanamido)-4-methylpent-4-enoic acid. | 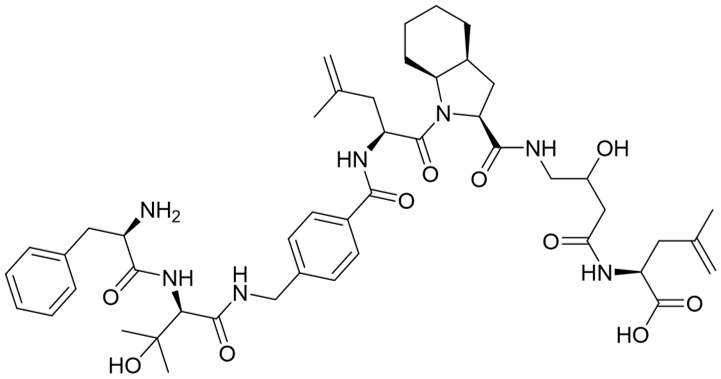 |
| 012 | Cyclic | C_39_H_59_N_7_O_11_ | 3-((3*S*,6*S*,9*S*,12*S*,15*S*,18*S*,23a*S*)-9-benzyl-6,18-di((*S*)-sec-butyl)-3,12-bis((*R*)-1-hydroxyethyl)-1,4,7,10,13,16,19-heptaoxodocosahydro-1H-pyrrolo[1,2-a][1,4,7,10,13,16,19]heptaazacyclohenicosin-15-yl)propanoic acid. | 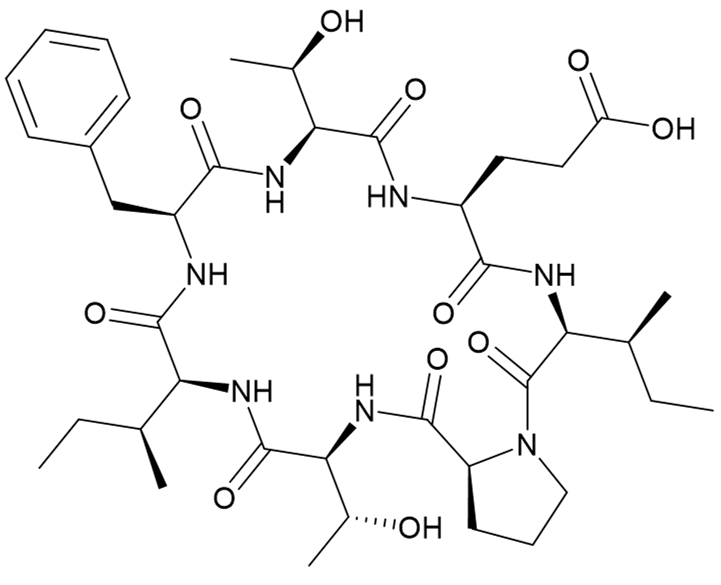 |
| 013 | Cyclic | C_63_H_97_N_13_O_20_ | (6*S*,9*S*,12*S*,15*S*,24*S*,29*S*,31a*S*,37*S*,40*S*,43*S*,45a*S*)-15-benzyl-6,40-di((*S*)-sec-butyl)-9-(2-carboxyethyl)-12,24,43-tris((*R*)-1-hydroxyethyl)-37-isobutyl-5,8,11,14,17,20,23,26,31,36,39,42,45-tridecaoxotetratetracontahydro-5H-dipyrrolo[2,1-c:2',1'-o][1,4,7,10,13,16,19,22,25,28,31,34,37]tridecaazacyclohentetracontine-29-carboxylic acid | 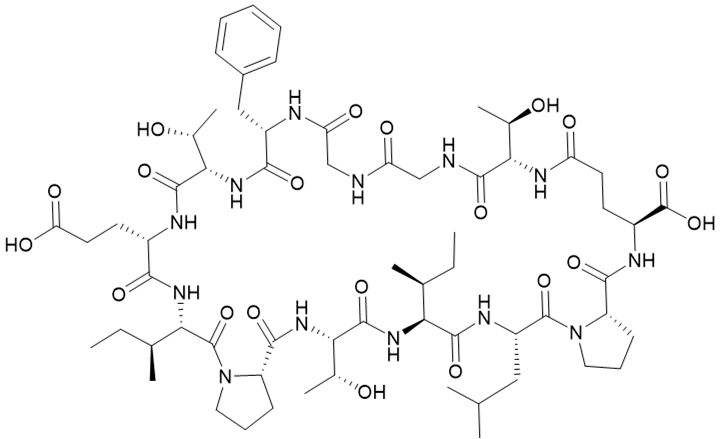 |
| 109 | Cyclic | C_49_H_71_N_7_O_9_ | (1^2^S,1^3a^S,1^7a^*S*,3*S*,10*S*,13*R*,16*S*,20*R*)-13-benzyl-20-hydroxy-10-(2-hydroxypropan-2-yl)-3,16-dineopentyl-1^2^,1^3^,1^3a^,1^4^,1^5^,1^6^,1^7^,1^7a^-octahydro-1^1^H-4,8,11,14,17,22-hexaaza-1(1,2)-indola-6(1,4)-benzenacyclotricosaphane-2,5,9,12,15,18,23-heptaone. | 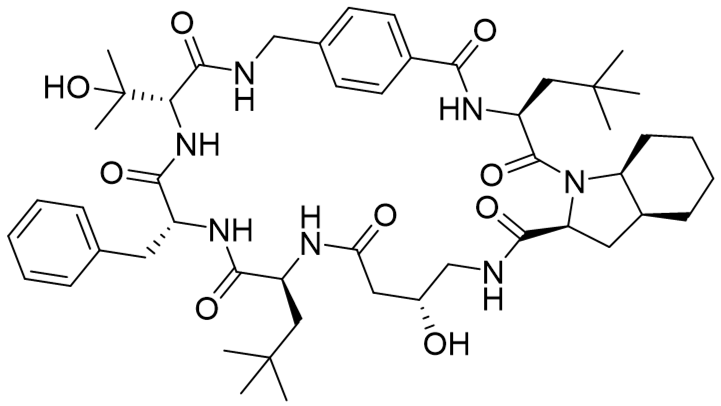 |
| 028 | Stapled | C_61_H_99_N_13_O_15_ | (4*R*)-4-((2*R*,3*S*)-2-((*R*)-2-amino-3-phenylpropanamido)-3-hydroxybutanamido)-5-(((3*S*,6*R*,9*S*,19*R*,22*R*,*Z*)-6-((*R*)-sec-butyl)-9-(((*S*)-1-carboxy-4-guanidinobutyl)carbamoyl)-3-((*R*)-1-hydroxyethyl)-9,19-dimethyl-22-neopentyl-1,4,7,20,23-pentaoxo-2,3,4,5,6,7,8,9,10,11,12,13,16,17,18,19,20,21,22,23,25,26,27,27a-tetracosahydro-1H-pyrrolo[1,2-d][1,4,7,10,13]pentaazacyclopentacosin-19-yl)amino)-5-oxopentanoic acid | 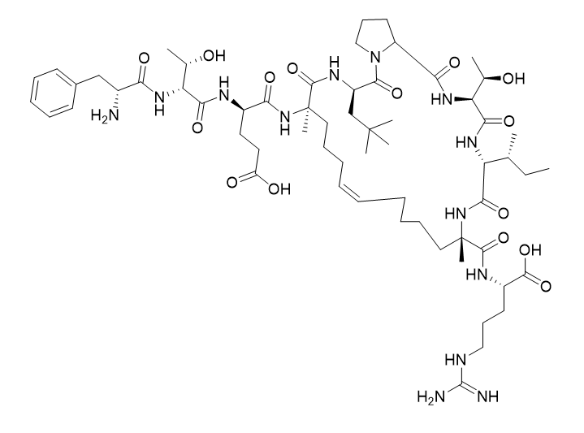 |
| 052 | Stapled | C_44_H_72_N_8_O_13_ | (4*R*)-4-((2*R*,3*S*)-2-amino-3-hydroxybutanamido)-5-(((3*S*,6*R*,15*R*,18*R*,Z)-6-(((1*S*,2*S*)-1-carboxy-2-methylbutyl)carbamoyl)-3-((*R*)-1-hydroxyethyl)-6,15-dimethyl-18-(2-methylallyl)-1,4,16,19-tetraoxo-2,3,4,5,6,7,8,9,12,13,14,15,16,17,18,19,21,22,23,23a-icosahydro-1H-pyrrolo[1,2-d][1,4,7,10]tetraazacyclohenicosin-15-yl)amino)-5-oxopentanoic acid | 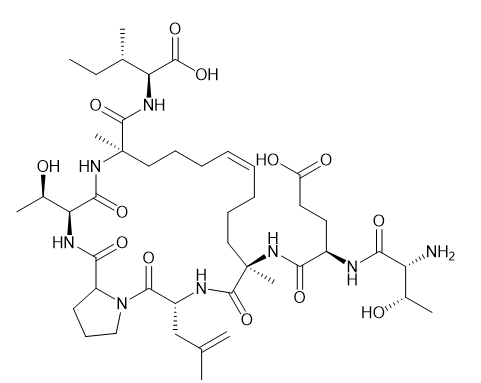 |
| 107 | Stapled | C_52_H_80_N_8_O_11_ | (2*R*)-2-((8*S*,17*R*,20*S*,22a*S*,26a*S*,27a*S*,*Z*)-17-(4-((4-amino-3-hydroxybutanamido)methyl)benzamido)-4-hydroxy-8,17-dimethyl-20-(2-methylallyl)-1,6,18,21-tetraoxo 2,3,4,5,6,7,8,9,10,11,14,15,16,17,18,19,20,21,22a,23,24,25,26,26a,27,27a-hexacosahydro-1H-[1,4,7,12]tetraazacyclotricosino[4,5-a]indole-8-carboxamido)-4,4-dimethylpentanoic acid | 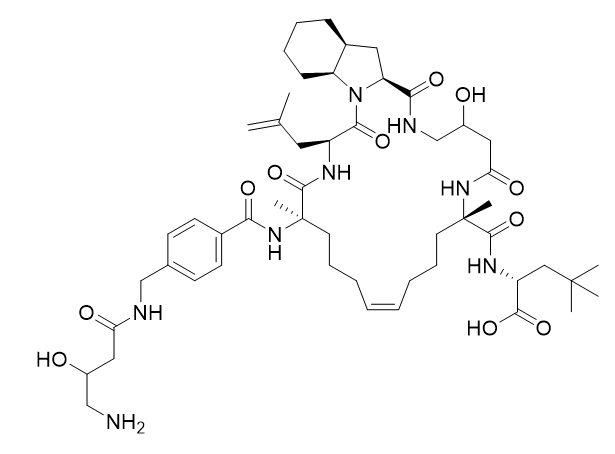 |
| 108 | Stapled | C_55_H_88_N_8_O_11_ | (2*R*)-2-((8*S*,18*R*,21*S*,23a*S*,27a*S*,28a*S*,Z)-18-(4-(((*R*)-2-amino-3-hydroxy-3-methylbutanamido)methyl)benzamido)-4-hydroxy-8,18-dimethyl-21-neopentyl-1,6,19,22-tetraoxo-1,2,3,4,5,6,7,8,9,10,11,12,15,16,17,18,19,20,21,22,23a,24,25,26,27,27a,28,28a-octacosahydro-[1,4,7,12]tetraazacyclotetracosino[4,5-a]indole-8-carboxamido)-4,4-dimethylpentanoic acid | 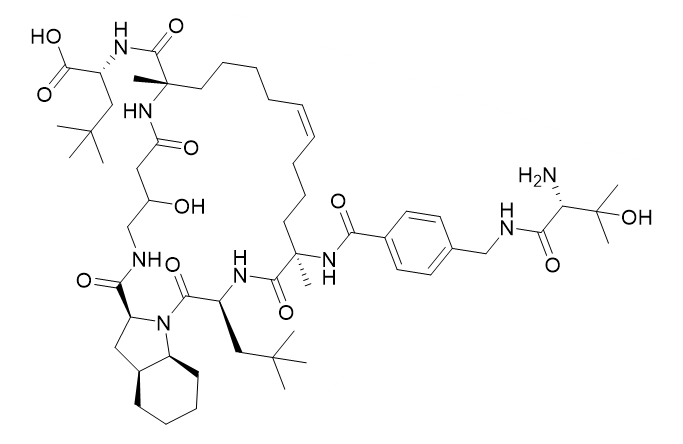 |

**Supplementary Table 1. Structures, IUPAC names, and the 2D chemical formulas of selected EB3 inhibitors**. See also Figures 1 and 2.

| **ID** | **MW (Da)** | **Amplification Factor (Fold Change)** | **Aggregation at 1mM** | **Log P**  **(Octanol/Water)** | **Competitive**  **Binding at 250nM**  **(% Inhibition)** |
| --- | --- | --- | --- | --- | --- |
| **Linear compounds** | | | | | |
| **000** | 820 | 1 |  | +0.75 (Myr) | no inhibition |
| **004** | 820 | 2.6 | 0.35 | -0.81 | 39 |
| **Liner compounds consisting of small protein-like chains** | | | | | |
| **016** | 848 | 3.9 | 0.18 | -0.30 | 48 |
| **019** | 818 | 3.9 | weak signal | -1.0 | 51 |
| **021** | 838 | 2.5 | weak signal | -0.24 | 35 |
| **036** | 832 | 2.9 | weak signal | -0.89 | 40 |
| **038** | 864 | 2.6 | 0.35 | -0.19 | 36 |
| **040** | 916 | 14.6 | weak signal | +0.67 | 50 |
| **041** | 906 | 2.5 | 0.18 | +0.71 | 30 |
| **042** | 881 | 22 | weak signal | +0.03 | 36 |
| **044** | 845 | 3.9 | weak signal | -0.59 | 51 |
| **049** | 830 | 11.5 | 0.31 | -0.71 | 52 |
| **101** | 919 | 6.6 | weak signal | +0.52 | 41 |
| **102** | 889 | 2.8 | weak signal | +0.20 | no inhibition |
| **103** | 772 | 2.8 | weak signal | +0.44 | 12 |
| **104** | 742 | 6.9 | weak signal | -0.3 | 40 |
| **105** | 887 | 3.6 | weak signal | -0.37 | 70 |
| **Cyclic compounds** | | | | | |
| **012** | 802 | 4.5 | 0.17 | -0.04 | 49 |
| **013** | 1357 | 3.8 | 0.17 | -0.27 | 51 |
| **109** | 902 | 23 | weak signal | +0.73 | 58 |
| **Stapled compounds** | | | | | |
| **028** | 1255 | 2.1 | 1.39 | -1.0 | 45 |
| **052** | 922 | 45.7 | 0.17 | -0.07 | 73 |
| **107** | 993 | 42.3 | weak signal | +0.51 | 65 |
| **108** | 1036 | 17.8 | weak signal | +0.46 | 55 |

**Supplementary Table 2. Summary of physicochemical and biochemical characteristics of selected EB3 inhibitors.** This table provides a summary of molecular weights, solubility at 1mM, binding affinities to purified full length EB3 protein (amplification factor), lipophilicity (*logP*), and effectiveness in inhibiting specific protein-protein interaction (competitive binding). See also Figures 1 and 2.

| **Time/Compound** | **Myr-EBIN** | **101** | **107** | **108** | **109** |
| --- | --- | --- | --- | --- | --- |
| **1.5 hr** | 105% | 87% | 87% | 80% | 90% |
| **20 hr** | 53% | 85% | 65% | 65% | 91% |

**Supplementary Table 3. Stability of selected EB3 inhibitors in human blood plasma.** The indicated inhibitors were incubated with human plasma at 37^°^C and samples were taken at the indicated time points. Plasma proteins were precipitated with ice-cold perchloric acid (60%); the concentration of compounds was measured in supernatant using a liquid chromatography-tandem mass spectrometry (LC-MS/MS)**.** See also Figures 1 and 2.

| **Species** | **t_½_ (min)** | **Average % remaining at last time point** |
| --- | --- | --- |
| **Human** | > 480 | 100.7 |
| **Rat** | 293 | 76.5 |
| **Mouse** | > 480 | 89.8 |
| **Dog** | > 480 | 91.9 |

**Supplementary Table 4. Stability of VT-109 in plasma of human, rodents, and dog.** VT-109 was incubated with species-specific plasma for 120 min and samples were taken at the required time points and measured as in Table 2. Minimal loss of VT-109 was observed in the presence of rat plasma, with a half-life of 293 minutes. VT-109 was stable in the presence of mouse, dog, and human plasma. See also Figures 1 and 2.

| **Storage Temp/ Compound** | **Myr-EBIN** | **101** | **107** | **108** | **109** |
| --- | --- | --- | --- | --- | --- |
| **-20 °C** | 94% | 102% | 94% | 104% | 97% |
| **4 °C** | 100% | 89% | 88% | 102% | 97% |
| **37 °C** | 95% | 96% | 85% | 104% | 85% |
| **60 °C** | 74% | 100% | 85% | 104% | 96% |

**Supplementary Table 5. Stability of selected compounds at various storage conditions.** Preliminary accelerated storage stability at various temperature conditions for the selected inhibitors was tested in phosphate-buffered sterile saline at -20^°^C, 4^°^C, 37^°^C and 60^°^C for 8 days. See also Figures 1 and 2.

| BAL Cytokine (pg/mL) | Sham Mean±Std | Vehicle Mean±Std | VT-109 Mean±Std | Combo  Mean±Std | Sham vs Vehicle p-value | Sham vs VT-109 p-value | Sham vs  Combo p-value | Vehicle vs VT-109 p-value | Vehicle vs Combo p-value | VT-109 vs Combo p-value |
| --- | --- | --- | --- | --- | --- | --- | --- | --- | --- | --- |
| mIL-1a | 1.73±0.22 | 0.38±0.733 | 0.14±0.00 | 0.14±0.00 | **0.0014** | **0.0002** | **0.0003** | >0.9999 | >0.9999 | >0.9999 |
| mIL-1b | 0.086±0.00 | 1.71±0.73 | 1.59±1.01 | 0.082±0.00 | **0.0011** | **0.0081** | >0.9999 | >0.9999 | **0.0019** | **0.0118** |
| mIL-3 | 0.28±0.00 | 0.72±0.18 | 0.28±0.00 | 0.28±0.00 | **0.0002** | >0.9999 | >0.9999 | **0.0002** | **0.0005** | >0.9999 |
| mIL-4 | 0.28±0.00 | 1.80±0.77 | 2.79±1.03 | 0.28±0.00 | **0.0111** | **0.0006** | >0.9999 | >0.9999 | **0.0161** | **0.0010** |
| mIL-5 | 4.90±0.28 | 0.64±1.30 | 2.79±1.03 | 0.21±0.00 | **0.0001** | 0.1413 | **<0.0001** | 0.3031 | >0.9999 | 0.1605 |
| mIL-6 | 3.44±9.32 | 93.87±124.30 | 282.12±522.24 | 357.30±851.22 | **0.0109** | **0.0073** | 0.1725 | >0.9999 | >0.9999 | >0.9999 |
| mIL-10 | 1.06±1.64 | 0.79±0.62 | 2.19±2.95 | 0.48±0.00 | >0.9999 | >0.9999 | >0.9999 | >0.9999 | >0.9999 | >0.9999 |
| mIL-12 | 2.60±1.56 | 21.07±4.11 | 16.99±7.90 | 1.80±0.00 | **0.0017** | **0.0253** | >0.9999 | >0.9999 | **0.0002** | **0.0047** |
| mIL-17 | 0.14±0.00 | 0.14±0.00 | 0.14±0.00 | 0.14±0.00 | >0.9999 | >0.9999 | >0.9999 | >0.9999 | >0.9999 | >0.9999 |
| mMCP-1 | 2.15±1.58 | 31.89±28.48 | 19.17±28.01 | 10.67±23.64 | **0.0032** | 0.1865 | >0.9999 | >0.9999 | **0.0074** | 0.2870 |
| mIFNγ | 1.15±0.19 | 0.44±0.70 | 0.49±0.34 | 0.21±0.00 | **0.0012** | **0.0291** | **0.0003** | >0.9999 | >0.9999 | >0.9999 |
| mTNFα | 0.16±0.00 | 2.72±0.97 | 0.43±0.77 | 2.30±0.17 | **0.0002** | >0.9999 | 0.0518 | **0.0009** | >0.9999 | 0.1349 |
| mMIP-1α | 0.51±0.39 | 0.62±0.82 | 1.16±1.31 | 0.98±1.27 | >0.9999 | 0.6736 | >0.9999 | 0.2254 | >0.9999 | 0.9220 |
| mGM-CSF | 0.52±0.69 | 0.60±1.23 | 1.21±0.70 | 0.15±0.00 | >0.9999 | 0.1075 | >0.9999 | 0.2310 | 0.8764 | **0.0047** |
| mRANTES | 0.14±0.00 | 3.46±7.29 | 2.55±5.23 | 1.57±3.88 | 0.3100 | **0.0027** | >0.9999 | 0.5797 | >0.9999 | **0.0386** |
| mKC | 12.94±14.25 | 413.55±245.90 | 609.75±878.78 | 522.37±678.55 | **0.0009** | **0.0142** | **0.0045** | >0.9999 | >0.9999 | >0.9999 |

**Supplementary Table 6. Analysis of inflammatory chemokines and cytokines in BAL of mice challenged with CLP and treated with either vehicle, VT-109, or combo therapy.** This table provides the levels of inflammatory chemokines and cytokines found in BAL of mice challenged with CLP and treated with either vehicle, VT-109, or combo therapy, same as Figure 5D. See also Figure 5.

| BAL Cytokine (pg/mL) | Sham Mean±Std | Vehicle Mean±Std | VT-109 Mean±Std | Combo  Mean±Std | Sham vs Vehicle p-value | Sham vs VT-109 p-value | Sham vs  Combo p-value | Vehicle vs VT-109 p-value | Vehicle vs Combo p-value | VT-109 vs Combo p-value |
| --- | --- | --- | --- | --- | --- | --- | --- | --- | --- | --- |
| mIL-1a | 0.14±0.00 | 113.17±115.39 | 104.93±94.65 | 43.32±21.34 | **0.0004** | **0.0016** | 0.0674 | >0.9999 | >0.9999 | >0.9999 |
| mIL-1b | 0.086±0.00 | 85.32±40.87 | 229.14±237.82 | 61.79±60.88 | **0.0019** | **0.0008** | 0.1038 | >0.9999 | >0.9999 | >0.9999 |
| mIL-3 | 0.28±0.00 | 0.28±0.00 | 0.28±0.00 | 0.28±0.00 | >0.9999 | >0.9999 | >0.9999 | >0.9999 | >0.9999 | >0.9999 |
| mIL-4 | 0.32±0.09 | 1.20±1.87 | 6.90±0.83 | 1.17±0.19 | >0.9999 | **<0.0001** | 0.0611 | **0.0023** | 0.8120 | 0.3648 |
| mIL-5 | 2.81±2.73 | 9.90±20.56 | 5.06±2.77 | 4.43±1.24 | >0.9999 | 0.9937 | >0.9999 | >0.9999 | >0.9999 | >0.9999 |
| mIL-6 | 41.96±19.56 | 18346±14985 | 46833±65942 | 22988±44555 | **0.0006** | **0.0032** | **0.0326** | >0.9999 | >0.9999 | >0.9999 |
| mIL-10 | 0.48±0.00 | 257.79±337.26 | 432.67±490.14 | 137.36±136.19 | **0.0023** | **0.0025** | **0.0339** | >0.9999 | >0.9999 | >0.9999 |
| mIL-12 | 18.76±3.95 | 45.45±17.12 | 57.48±43.37 | 28.82±6.50 | **0.0009** | **0.0010** | 0.2895 | >0.9999 | 0.6121 | 0.5874 |
| mIL-17 | 0.14±0.00 | 13.20±13.67 | 7.20±5.26 | 14.91±9.17 | **0.0077** | 0.0797 | **0.0006** | >0.9999 | >0.9999 | 0.7908 |
| mMCP-1 | 24.11±20.27 | 12200±8195 | 16338±17531 | 10062±10520 | **0.0012** | **0.0024** | **0.0225** | >0.9999 | >0.9999 | >0.9999 |
| mIFNγ | 0.21±0.00 | 1.24±3.10 | 4.39±3.00 | 0.70±1.28 | >0.9999 | **0.0003** | >0.9999 | **0.0019** | >0.9999 | **0.0171** |
| mTNFα | 0.16±0.00 | 20.87±20.56 | 29.39±31.64 | 8.80±5.58 | **0.0012** | **0.0005** | 0.0674 | >0.9999 | >0.9999 | >0.9999 |
| mMIP-1α | 0.49±0.34 | 1930±1911 | 5136±8056 | 923.46±785.81 | **0.0012** | **0.0006** | 0.0647 | >0.9999 | >0.9999 | >0.9999 |
| mGM-CSF | 1.65±0.34 | 9.57±5.49 | 15.75±20.22 | 4.49±4.86 | 0.1745 | 0.5901 | >0.9999 | >0.9999 | 0.3882 | >0.9999 |
| mRANTES | 1.30±0.65 | 347.17±228.41 | 382.32±397.78 | 264.91±334.25 | **0.0010** | **0.0017** | **0.0356** | >0.9999 | >0.9999 | >0.9999 |
| mKC | 19.46±15.41 | 9819±9718 | 24611±34486 | 9965±18000 | **0.0010** | **0.0011** | 0.0507 | >0.9999 | >0.9999 | >0.9999 |

**Supplementary Table 7. Analysis of inflammatory chemokines and cytokines in PF of mice challenged with CLP and treated with either vehicle, VT-109, or combo therapy.** This table provides the levels of inflammatory chemokines and cytokines found in PF of mice challenged with CLP and treated with either vehicle, VT-109, or combo therapy, same as Figure 5D. See also Figure 5.

| Treatment (mg/kg) | 0 | 5 | 10 | 15 |
| --- | --- | --- | --- | --- |
| Group No. | 4 | 1 | 3 | 2 |
| Number of Animals | 3 | 3 | 3 | 3 |
| Clinical Signs | DA | DA | DA, LB | DA, LB, P, HP |
| Body Weights | - | NE | NE | NE |
| Clinical Chemistry | - | NE | ↑BUN | ↑BUN  ↑Creatinine |
| Hematology | - | NE | ↓%Reticulocytes | ↓Platelet Count  ↓%Reticulocytes  ↑Mean Platelet Volume |
| Coagulation | - | NE | NE | NE |

**Supplementary Table 8. Summary of clinical signs and observations of a non-GLP dose-escalation study in rats.** Nine rats were dosed with VT-109 at 5 mg/kg by IV tail vein bolus injection and three control rats were given IV tail vein bolus of emulsion formulation only. NE = no effect. DA = decreased activity. LB = labored breathing. P = piloerection. HP = hunched posture.

| Sex: Male | Day(s) Relative to Start Date | | Vehicle  Dose | 1M  5 mg/kg | 3M  10 mg/kg | 2M  15 mg/kg |
| --- | --- | --- | --- | --- | --- | --- |
| Sodium | 2 | Mean | 143.0 I,a¹ | 141.3 | 144.0 | 142.0 |
| (mmol/L) |  | SD | 1.0 | 0.6 | 1.0 | 0.0 |
|  |  | N | 3 | 3 | 3 | 3 |
| Potassium | 2 | Mean | 6.37 ⁵ | 5.93 d² | 5.77 dd⁴ | 5.47 ddd³ |
| (mmol/L) |  | SD | 0.21 | 0.21 | 0.15 | 0.15 |
|  |  | N | 3 | 3 | 3 | 3 |
| Chloride | 2 | Mean | 101.7 I,a¹ | 98.3 | 99.0 | 97.0 d² |
| (mmol/L) |  | SD | 1.2 | 0.6 | 2.0 | 1.7 |
|  |  | N | 3 | 3 | 3 | 3 |
| Alkaline | 2 | Mean | 436.3 I^6^ | 381.7 | 323.0 | 286.0 |
| Phosphatase |  | SD | 89.9 | 137.4 | 83.2 | 54.0 |
| (U/L) |  | N | 3 | 3 | 3 | 3 |
| ALT | 2 | Mean | 60.3 I^6^ | 52.7 | 52.0 | 52.7 |
| (U/L) |  | SD | 13.1 | 11.6 | 4.4 | 9.3 |
|  |  | N | 3 | 3 | 3 | 3 |
| AST | 2 | Mean | 74.7 I^6^ | 71.3 | 68.3 | 69.7 |
| (U/L) |  | SD | 9.3 | 6.1 | 10.5 | 6.8 |
|  |  | N | 3 | 3 | 3 | 3 |
| Total | 2 | Mean | 0.133 I^6^ | 0.127 | 0.127 | 0.140 |
| Bilirubin |  | SD | 0.006 | 0.006 | 0.015 | 0.026 |
| (mg/dL) |  | N | 3 | 3 | 3 | 3 |
| Blood Urea | 2 | Mean | 13.0 I,a¹ | 13.0 | 18.7 ddd³ | 15.7 d² |
| Nitrogen |  | SD | 1.7 | 1.0 | 0.6 | 0.6 |
| (mg/dL) |  | N | 3 | 3 | 3 | 3 |
| Creatinine | 2 | Mean | 0.29360 I,aaa^7^ | 0.31390 | 0.31803 | 0.35203 dd⁴ |
| (mg/dL) |  | SD | 0.02220 | 0.01792 | 0.00404 | 0.01085 |
|  |  | N | 3 | 3 | 3 | 3 |
| Calcium | 2 | Mean | 11.237 I^6^ | 11.320 | 11.767 | 11.590 |
| (mg/dL) |  | SD | 0.254 | 0.075 | 0.407 | 0.098 |
|  |  | N | 3 | 3 | 3 | 3 |
| Glucose  (mg/dL) | 2 | Mean  SD  N | 123.0 ³  5.6  3 | 161.3 ddd¹  7.4  3 | 126.7  10.5  3 | 142.3 d²  1.2  3 |
| Albumin  (g/dL) | 2 | Mean  SD  N | 3.3127 ⁵  0.0369  3 | 3.0400 dd⁴  0.0834  3 | 3.2720  0.1274  3 | 3.1030 d²  0.0465  3 |
| Total  Protein  (g/dL) | 2 | Mean  SD  N | 5.955 ³  0.054  3 | 5.279 ddd¹  0.084  3 | 5.795  0.203  3 | 5.591 d²  0.067  3 |
| Phosphorus  (mg/dL) | 2 | Mean  SD  N | 8.90 I^6^  0.26  3 | 9.10  0.44  3 | 9.67  0.45  3 | 9.00  0.26  3 |
| Cholesterol  (mg/dL) | 2 | Mean  SD  N | 94.3 I^6^  2.1  3 | 86.0  5.6  3 | 88.7  2.3  3 | 91.0  6.1  3 |
| Globulin  (g/dL) | 2 | Mean  SD  N | 2.642 ³  0.069  3 | 2.239 ddd¹  0.096  3 | 2.523  0.078  3 | 2.488  0.021  3 |
| A/G Ratio | 2 | Mean  SD  N | 1.255 L^7^  0.042  3 | 1.360  0.086  3 | 1.297  0.017  3 | 1.247  0.009  3 |
| Amylase  (mg/dL) | 2 | Mean  SD  N | 692.0 ³  45.7  3 | 472.7 dd⁴  42.4  3 | 496.3 dd⁴  76.7  3 | 422.0 ddd¹  26.9  3 |

**Supplementary Table 9. Summary of clinical chemistry tests in rats.** This table provides the summary of the clinical chemistry tests in rats. ddd = Dunnett 2 Sided p < 0.001. d = Dunnett 2 Sided p < 0.05. I,aaa = Automatic Transformation: Identity (No Transformation), (All Groups) Analysis of Variance p < 0.001. dd = Dunnett 2 Sided p < 0.01. I,aa = Automatic Transformation: Identity (No Transformation), (All Groups) Analysis of Variance p < 0.01. I = Automatic Transformation: Identity (No Transformation). L = Automatic Transformation Log.

| Sex: Male | Day(s) Relative to Start Date | | Vehicle  Dose | 1M  5 mg/kg | 3M  10 mg/kg | 2M  15 mg/kg |
| --- | --- | --- | --- | --- | --- | --- |
| APTT | 2 | Mean | 14.000 I,a² | 17.933 d¹ | 17.800 d¹ | 16.867 |
| (Seconds) |  | SD | 1.769 | 1.210 | 2.095 | 0.808 |
|  |  | N | 3 | 3 | 3 | 3 |
| Prothrombin | 2 | Mean | 11.00 I,a² | 11.77 | 12.27 d¹ | 11.67 |
| Time |  | SD | 0.46 | 0.12 | 0.60 | 0.31 |
| (Seconds) |  | N | 3 | 3 | 3 | 3 |
| Fibrinogen | 2 | Mean | 409.3 I³ | 403.0 | 453.3 | 438.3 |
| (mg/dL) |  | SD | 18.9 | 32.0 | 37.6 | 38.7 |
|  |  | N | 3 | 3 | 3 | 3 |

**Supplementary Table 10. Summary of coagulation tests.** This table provides a summary of the coagulation test. d = Dunnett 2 Sided p < 0.05. I,a = Automatic Transformation: Identity (No Transformation), (All Groups) Analysis of Variance p < 0.05. I = Automatic Transformation: Identity (No Transformation).

| LLOQ | 1.8 | 1.4 | ng/mL |
| --- | --- | --- | --- |
| ULOQ | 7190 | 2810 | ng/mL |
| Time‐Point (hour) |  | Concentration ng/mL |  |
|  | P109 (+Mode) Peak 1 | P109 (+Mode) Peak2 | P109 Total |
| 0.033 | 2.8 | 1.7 | 4.5 |
| 0.033 | 36230.5 | 44861.8 | 81092.3 |
| 0.167 | 7671.6 | 15988.2 | 23659.9 |
| 0.50 | 939.8 | 1614.6 | 2554.4 |
| 1 | 279.9 | 300.2 | 580.0 |
| 1.5 | 190.1 | 208.6 | 398.7 |
| 2 | 122.5 | 129.0 | 251.5 |
| 4 | 59.9 | 67.4 | 127.3 |
| 6 | 31.5 | 34.7 | 66.3 |
| 8 | 20.6 | 22.0 | 42.7 |
| 24 | 2.7 | 3.2 | 5.9 |

**Supplementary Table 11. Summary of plasma drug levels in rats.** This table provides a summary of the plasma drug levels of rats in mean concentrations (ng/mL).

| **Parameter** | **Units** | **Estimate** |
| --- | --- | --- |
| R-squared |  | 0.9950 |
| Lambda_z | 1/hr | 0.1299 |
| Half-Life | hr | 5.33 |
| Tmax | hr | 0.033 |
| Cmax | ng/mL | 40548 |
| C0 | ng/mL | 46301 |
| Tlast | hr | 24 |
| Clast | ng/mL | 5.93 |
| AUC (0-last) | hr*ng/mL | 12361 |
| AUC (0-inf) | hr*ng/mL | 12406 |
| AUC_%Extrap_obs | % | 0.3681 |
| AUC_%Back_Ext_obs | % | 11.55 |
| Volume of Distribution | mL/kg | 3102 |
| Clearance | mL/hr/kg | 403.0 |

**Supplementary Table 12. Summary of pharmacokinetic of VT-109 in rat plasma.** This table provides a summary of pharmacokinetics of VT-109 in rat plasma.

| Parameter | Units | Estimate |
| --- | --- | --- |
| r-squared |  | 0.9713 |
| Lambda_z | 1/hr | 0.1535 |
| Half-Life | hr | 4.52 |
| Tmax | hr | 2.00 |
| Cmax | ng/g | 3606 |
| Tlast | hr | 24.00 |
| Clast | ng/mg | 105.7 |
| AUC (0-last) | hr*ng/g | 27219 |
| AUC (0-inf) | hr*ng/g | 27908 |
| AUC_%Extrap_obs | % | 2.47 |
| AUC_%Back_Ext_obs | % | 5.73 |

**Supplementary Table 13. Summary of pharmacokinetic of VT-109 in rat lungs.** This table provides a summary of pharmacokinetics of VT-109 in rat lungs.
